# Causal association of circulating inflammatory proteins on neurodegenerative diseases: Insights from a mendelian randomization study

**DOI:** 10.1111/jcmm.70176

**Published:** 2024-10-29

**Authors:** Wenwen Lin, Xuewei Wu, Guanyong Ou

**Affiliations:** ^1^ Department of Pathology, Shenzhen Hospital Southern Medical University Shenzhen Guangdong P. R. China; ^2^ National Clinical Research Center for Infectious Disease, The Third People's Hospital of Shenzhen Second Hospital Affiliated to Southern University of Science and Technology Shenzhen Guangdong P. R. China; ^3^ School of Medicine Southern University of Science and Technology Shenzhen Guangdong P. R. China

**Keywords:** causality, genome‐wide association studies, inflammatory cytokines, mendelian randomization, neurodegenerative diseases

## Abstract

Neuroinflammation is increasingly recognized as a pivotal factor in the development and progression of neurodegenerative disorders. While correlations between inflammatory cytokines and these diseases are documented, the definitive causal dynamics remain to be elucidated. We explored the causal association between 91 circulating inflammatory cytokines and Alzheimer's disease (AD), amyotrophic lateral sclerosis (ALS), multiple sclerosis (MS) and Parkinson's disease (PD) through Mendelian randomization analysis. Leveraging genetic variants from the most comprehensive genome‐wide association studies (GWAS) available for these cytokines, AD, ALS, MS and PD, we sought to uncover the causality. Our study validated a causal influence of genetically determined cytokine levels on the susceptibility to AD, with notable cytokines including C‐X‐C motif chemokine 1 (OR = 0.9993, *p* = 0.0424), Interleukin‐18 (OR = 0.9994, *p* = 0.0186), Leukaemia inhibitory factor receptor (OR = 0.9993, *p* = 0.0122) and Monocyte chemoattractant protein‐1 (OR = 0.9992, *p* = 0.0026) in risk attenuation. Additionally, a positive causal relationship was identified between two cytokines—C‐C motif chemokine 19 (OR = 1.0005, *p* = 0.0478) and Fms‐related tyrosine kinase 3 ligand (OR = 1.0005, *p* = 0.0210)—and AD incidence. Conversely, transforming growth factor‐alpha (OR = 0.8630, *p* = 0.0298), CD40L receptor (OR = 0.7737, *p* = 1.1265E‐09) and Interleukin‐12 subunit beta (OR = 0.8987, *p* = 0.0333) showed inverse associations with ALS, MS and PD, respectively. The consistency observed in various MR analyses, alongside sensitivity analysis, underscored the absence of horizontal pleiotropy, thus supporting our causal findings. This study reveals, for the first time, a genetically anchored causal nexus between levels of circulating inflammatory cytokines and the risk of neurodegenerative diseases.

## 1 INTRODUCTION

Neurodegenerative diseases (NDs) such as Alzheimer's disease (AD), amyotrophic lateral sclerosis (ALS), multiple sclerosis (MS) and Parkinson's disease (PD) represent a complex spectrum of disorders marked by the progressive deterioration and loss of neuronal structure and function.^1^ Despite considerable advancements in understanding the pathophysiology of these conditions, the etiologies remain multifaceted and incompletely understood. One emerging facet central to NDs is neuroinflammation, which has been implicated in their pathogenesis.^2^ Inflammatory cytokines, small signalling proteins released by immune cells, are known to play a dual role in the central nervous system, contributing to both homeostasis and the propagation of inflammatory responses.^3^ It has been reported that neuroinflammation and activated microglia are characteristic features of AD, PD and other NDs, evidenced by increased levels of pro‐inflammatory cytokines in the bloodstream and cerebrospinal fluid.^4^ For example, microglia in the nervous system activate NLRP3 inflammasomes and release cytokines in response to the detection of protein misfolding deposition or amyloid beta (Aβ) aggregation, thereby promoting the onset and progression of neurodegenerative diseases. There is substantial evidence to suggest that NLRP3 inflammasome are promising therapeutic targets for developing pharmacological strategies for clinical NDs management. The pharmacological inhibition of NLRP3 prevented α‐synuclein pathology and dopaminergic neurodegeneration.^5^ Growing evidence showed that inflammation may be a primary factor driving disease pathology, rather than a secondary consequence.

The relationship between inflammatory cytokines and NDs has been a subject of extensive research. Observational studies have consistently reported elevated levels of various cytokines in patients with NDs, suggesting a potential link.^6^ However, whether this association is causal or merely a consequence of the underlying neurodegenerative process is not well established. To address this critical gap in knowledge, our study employs Mendelian randomization (MR), a method that uses genetic variants as instrumental variables to infer causality,^7^, ^8^ to dissect the influence of circulating inflammatory cytokines on the risk of developing NDs. We believe that further understanding of the inflammatory drivers of NDs and the development of key therapeutic targets are essential, which will be instrumental in advancing the development of pharmacological strategies for the effective management of clinical NDs.

MR harnesses genetic variants intrinsically linked to potential exposures as instrumental variables (IVs) to discern the causal relationships between exposures and outcomes.^7^, ^8^ This methodology triumphs over observational studies by obviating the pitfalls of reverse causation and confounding. The advent of genome‐wide association studies (GWAS) in the preceding decade has unearthed an abundance of genetic variants intricately associated with complex traits, thereby illuminating the genetic architecture underlying these traits.^9^, ^10^ The utilization of these associated variants as IVs propels the MR approach forward in unravelling the etiological factors of multifaceted diseases, such as AD,^11^, ^12^ sepsis,^13^, ^14^ lichen simplex chronicus^15^ and psoriasis vulgaris.^16^, ^17^ Collectively, these studies exemplify MR's prowess in inferring causality between genetic predispositions to increased exposure levels and disease risk, all the while mitigating biases innate to conventional observational research.^8^, ^18^

In the present investigation, we pioneer the application of MR to investigate the causal nexus between 91 circulating inflammatory proteins and ND. By employing IVs as proxies for circulating cytokines, we aim to ascertain whether inflammation is a driving force in NDs pathogenesis or merely a secondary phenomenon.

## 2 MATERIAL AND METHOD

### 2.1 Study design

**FIGURE 1:**
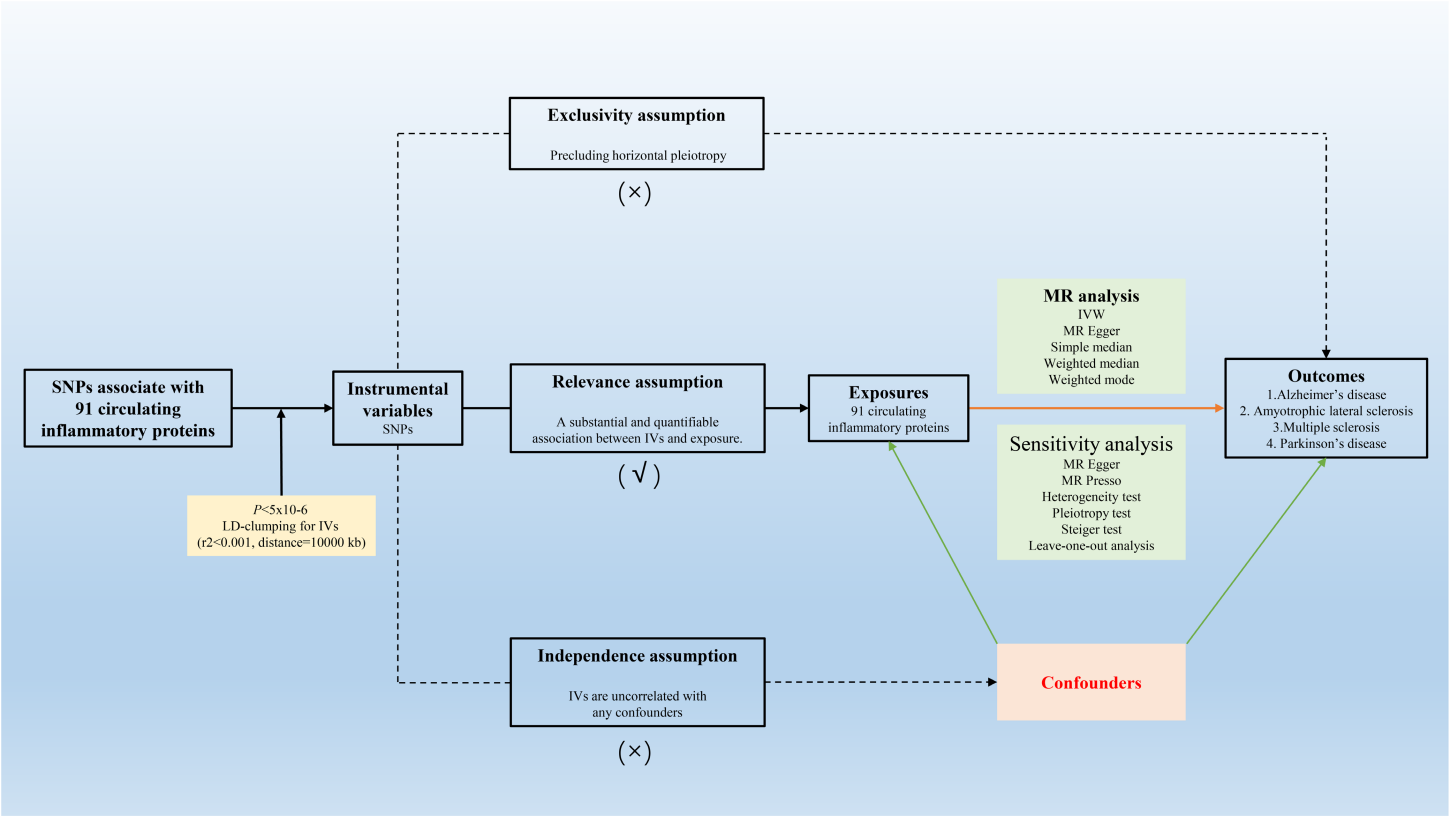
MR analysis framework. SNPs: Single nucleotide polymorphisms. IVs: Instrumental variables. A √ symbol denotes that the criteria were satisfied; a × symbol denotes that the criteria were not satisfied.

In this two‐sample MR study (Figure 1), we have strictly adhered to a set of three core assumptions that are crucial for ensuring the empirical robustness and methodological soundness of our study. To ensure the integrity and validity of our data, single nucleotide polymorphisms (SNPs) were exploited as IVs based on the following foundational assumptions^19^:

1. Relevance assumption: We applied the relevance criterion, which requires a substantial and quantifiable association between the IVs and the exposure variables.

2. Exclusivity assumption: We followed the exclusivity criterion, which states that the IVs must solely affect the outcome through their impact on the exposure variable, excluding any form of horizontal pleiotropy.

3. Independence assumption: The independence criterion mandates that the IVs be uncorrelated with any confounders associated with both the exposure and the outcome.

These assumptions, as delineated by Lawlor et al. (2008), are essential for maintaining the integrity of the MR study design and the credibility of its findings.^20^

### 2.2 Genome‐wide association summary data resource

SNPs on 91 circulating inflammatory proteins and neurodegenerative diseases (including AD, ALS, MS and PD) were retrieved from the largest publicly available GWAS database. In brief, we accessed genome‐wide association summary‐level statistics for 91 circulating inflammatory proteins, AD, ALS, MS and PD. The GWAS data for 91 circulating inflammatory proteins stored in the GWAS Catalogue database (https://www.ebi.ac.uk/gwas/publications/37563310) were extracted from a genome‐wide protein quantitative trait locus (pQTL) study of 91 plasma proteins measured using the Olink Target platform in 14,824 participants.^21^ To investigate the causal impact of exposure to various circulating inflammatory proteins on the risk of different neurodegenerative diseases, we utilized datasets for AD, ALS, MS and PD as the outcome from the summary statistics of the GWAS summary data from the IEU OpenGWAS project database (Table 1). Subsequently, we employed two‐sample MR methods using GWAS summary statistics to infer the causal relationship between inflammatory cytokines and these neurodegenerative diseases. As the samples of circulating inflammatory proteins and neurodegenerative diseases were obtained from different consortiums, there was no overlap and therefore, no ethical approval was required.

**TABLE 1:** Summary of GWAS datasets.

GWAS ID	Year	Trait	Consortium	nCase	nControl	Sample size	nSNPs
ieu‐b‐5067	2022	AD	NA	954	487,331	488,285	12,321,875
ebi‐a‐GCST005647	2018	ALS	NA	20,806	59,804	80,610	39,630,630
ieu‐b‐18	2019	MS	International Multiple Sclerosis Genetics Consortium	47,429	68,374	115,803	6,304,359
ieu‐b‐7	2019	PD	International Parkinson's Disease Genomics Consortium	33,674	449,056	482,730	17,891,936

### 2.3 SNPs selection

To ensure the selection of valid SNPs that meet the stringent criteria of MR analysis, we implemented a thorough filtering strategy. Initially, we chose independent SNPs with a strong association with various circulating inflammatory proteins, setting a significance threshold for SNP‐cytokine associations at a *p*‐value <5 × 10^−6^ to expand potential IVs. We then used a clumping procedure to dismantle any existing linkage disequilibrium (LD) among the SNPs, employing a stringent r^2^ threshold of <0.001 and a clumping distance of 10,000 kb.^22^, ^23^ Any SNPs in LD were carefully removed to maintain the integrity of our causal inferences and prevent bias. We further refined the selection by utilizing the PhenoScanner V2 database (http://www.phenoscanner.medschl.cam.ac.uk/) to access comprehensive phenotypic data for our screened SNPs, ensuring they did not have pleiotropic effects that could confound their role as IVs.^24^ This rigorous harmonization process was crucial in identifying a set of robust genetic IVs for our MR analyses, examining the causal effects linking inflammatory proteins to the risk of ND. Additionally, we calculated the F statistics for the SNPs using the equation: F = R^2^ × (N–2)/(1–R^2^), where R^2^ represents the proportion of variance and N is the sample size.^25^ Weak instruments, defined as IVs with an F statistic of less than 10 (F < 10), were excluded from the analysis.

### 2.4 Mendelian randomization analysis

Following the meticulous selection of SNPs meeting stringent criteria for genetic IVs, we adopted the inverse variance weighted (IVW) method (1, 17) as the foundation of our MR analysis. This method combines causal effect estimates from multiple genetic variants, assuming the validity of each IV without the influence of heterogeneity or pleiotropy. It relies on the collective SNPs to provide an unbiased estimation of the causal relationship and is considered the most precise technique for causal inference, contingent on the validity of all included SNPs. To enhance the robustness of our causal assessment and address potential violations of IV assumptions, we integrated a range of complementary MR methods. These included the Weighted Median,^26^ MR‐Egger,^27^ Weighted Mode^28^ and Simple Mode approaches.^29^ The Weighted Median estimator delivers a reliable causal estimate when a majority (over 50%) of the information is contributed by valid SNPs. The MR‐Egger method, with its intercept term, can yield unbiased causal estimates even in the presence of pleiotropic effects, assuming Instrument Strength Independent of Direct Effect (InSIDE). We rigorously cross‐validated our causal inferences, requiring consistency across these diverse methodologies to bolster the credibility of our findings. Our multifaceted analytical strategy, incorporating an array of MR techniques, was employed to ensure the most comprehensive and reliable elucidation of the causal pathways under investigation.

### 2.5 Test for heterogeneity and pleiotropy

To rigorously scrutinize the foundational assumption of MR, which posits that genetic IVs influence the outcome solely through the exposure, we conducted a comprehensive suite of diagnostic evaluations aimed at detecting heterogeneity and pleiotropy within our IVs. The MR Pleiotropy Residual Sum and Outlier (MR‐PRESSO)^30^ test was used to investigate directional pleiotropy and identify potential outliers, thereby enhancing the accuracy of the MR analysis. To address horizontal pleiotropy, we utilized the MR‐Egger regression intercept term as an estimation tool,^27^ where a statistically significant deviation from zero (*p* < 0.05) indicates the presence of horizontal pleiotropy, requiring careful interpretation of the MR findings. Heterogeneity among the causal effect estimates of the IVs was quantified using Cochran's *Q*‐statistic,^7^ critical for discerning whether the variability in the causal estimates exceeds that expected by chance alone, suggesting the presence of invalid IVs or multiple underlying causal pathways. Further validation of the causal directionality was ascertained using the Steiger test^31^ to confirm that the exposure precedes the outcome, with a significance level of *p* < 0.05 indicating the exposure is indeed upstream of the outcome. Lastly, a leave‐one‐out sensitivity analysis was employed to systematically re‐evaluate the causal estimate by excluding one IV at a time, instrumental in determining the robustness and consistency of our results. Together, these methodological checks provide a rigorous framework for validating the MR assumptions, enhancing the credibility and reliability of our causal conclusions drawn from the MR analysis.

### 2.6 Druggable protein identification

DGIdb^32^ databases was used to further assess whether the identified proteins can serve as potential therapeutic targets by searching the interactions between these proteins and drugs.

### 2.7 Statistical analysis

The analytical procedures were conducted utilizing the ‘TwoSampleMR’ and ‘MRPRESSO’ packages within the R computational environment, version 4.2.1. A *p*‐value threshold of less than 0.05 was established as the criterion for statistical significance.

## 3 RESULTS

### 3.1 Causal effects of circulating inflammatory cytokine levels on risk of neurodegenerative diseases

To elucidate the intricate web of causality linking circulating cytokines with neurodegenerative diseases, we executed an exhaustive MR investigation. Adhering to the rigorous selection mandates depicted in our methodological blueprint (Figure 1), we pinpointed an array of SNPs with quantities spanning from 3 to 31, which served as IVs for the quantitative trait loci scrutiny of 91 circulating cytokines (Table S1—Data S1).

These selected SNPs were then harnessed to dissect the causality. To summarize succinctly, preliminary insights gleaned from the IVW method unravelled the causal effect of 23 cytokines on four distinct neurodegenerative conditions. Within these cytokines, a subset of 10 was found to exert a contributory causation towards the diseases, whereas the remaining 17 demonstrated an inverse association with the susceptibility to these conditions (Table S1—Data S1). Delving into AD, our research illuminated a positive causal influence exerted by three specific cytokines on the likelihood of disease manifestation, inclusive of C‐C motif chemokine 19 (OR = 1.0005, *p* = 0.0478), Fms‐related tyrosine kinase 3 ligand (OR = 1.0005, *p* = 0.0210) and SIR2‐like protein 2 (OR = 1.0010, *p* = 0.0155), alongside the revelation of a protective causal effect attributed to four other cytokines, namely C‐X‐C motif chemokine 1 (OR = 0.9993, *p* = 0.0424), Interleukin‐18 (OR = 0.9994, *p* = 0.0186), Leukaemia inhibitory factor receptor (OR = 0.9993, *p* = 0.0122) and Monocyte chemoattractant protein‐1 (OR = 0.9992, *p* = 0.0026) (Table S1—Data S1). For ALS, the investigation brought to light a positive causal effect of two cytokines—Hepatocyte growth factor (OR = 1.1291, *p* = 0.0304) and Tumour necrosis factor ligand superfamily member 12 (OR = 1.0981, *p* = 0.0163), and a negative causal effect of five others, among them C‐X‐C motif chemokine 10 (OR = 0.8862, *p* = 0.0071), C‐X‐C motif chemokine 11 (OR = 0.9034, *p* = 0.0316), Interleukin‐2 receptor subunit beta (OR = 0.8788, *p* = 0.0486), T‐cell surface glycoprotein CD5 (OR = 0.8885, *p* = 0.0216) and Transforming growth factor‐alpha (OR = 0.8630, *p* = 0.0298). In the context of MS, the results noted a positive causal effect of four cytokines, including C‐X‐C motif chemokine 10 (OR = 1.2726, *p* = 0.0120), Leukaemia inhibitory factor receptor (OR = 1.1819, *p* = 0.0254), Natural killer cell receptor 2B4 (OR = 1.2092, *p* = 0.0192) and T‐cell surface glycoprotein CD6 isoform (OR = 1.1379, *p* = 0.0027), contrasted with a negative causal effect of three cytokines, namely Adenosine Deaminase (OR = 0.7675, *p* = 0.0024), CD40L receptor (OR = 0.7737, *p* = 1.1265E‐09) and Neurturin (OR = 0.7001, *p* = 0.0042) (Table S1—Data S1). Pertaining to PD, our findings indicated a singular cytokine, Interleukin‐17A (OR = 1.2852, *p* = 0.0145), as having a positive causal effect on the risk of developing the disease, with a contrasting negative causal effect observed for five cytokines, which include Fibroblast growth factor 21 (OR = 0.8169, *p* = 0.0387), Interleukin‐12 subunit beta (OR = 0.8987, *p* = 0.0333), Neurturin (OR = 0.7346, *p* = 0.0049), Transforming growth factor‐alpha (OR = 0.8254, *p* = 0.0478) and Tumour necrosis factor receptor superfamily member 9 (OR = 0.8451, *p* = 0.0105) (Table S1—Data S1). The characteristics of the IVs associated with these implicated circulating cytokines are detailed in Table S2—Data S1. Each selected SNP was validated as a robust instrument, as evidenced by F‐statistic values exceeding the threshold of 10, indicative of strong instrument validity.

The MR‐derived estimations delineating the causal dynamics between the 23 circulating cytokines and these four neurodegenerative diseases, ascertained through a variety of analytical approaches such as MR Egger, simple median, weighted median and weighted mode, are meticulously listed in Table S3—Data S1, further reinforcing the credibility of the causal inferences drawn from our study.

### 3.2 Sensitivity analyses

In the refined analysis of heterogeneity and pleiotropy, the results for AD and ALS from both the IVW and MR Egger regression methods indicated an absence of statistically significant heterogeneity across the studied genetic instruments (all *p*‐values >0.05), as detailed in Table 2. However, three cytokines (Natural killer cell receptor 2B4, T‐cell surface glycoprotein CD6 isoform and Leukaemia inhibitory factor receptor) for MS and one cytokine (Fibroblast growth factor 21) for PD, the IVW and MR Egger regression methods indicated a statistically significant heterogeneity, with a *p*‐values <0.05 (Table 2). Notably, for four neurodegenerative diseases, there was no significant horizontal pleiotropy (*p*‐values >0.05) (Table 2).

**TABLE 2:** Heterogeneity and directional horizontal pleiotropy tests of circulating inflammatory cytokines on AD, ALS, MS and PD.

Exposure	Outcome	Heterogeneity test				Directional horizontal pleiotropy test		
		IVW		MR Egger		Egger intercept	SE	*p*‐value
		*Q*	*Q*_ *p*‐value	*Q*	*Q*_ *p*‐value			
C‐C motif chemokine 19 levels	AD	19.4804	0.4908	17.4115	0.5620	8.10E‐05	5.63E‐05	0.1666
C‐X‐C motif chemokine 1 levels	AD	13.8207	0.1813	13.8024	0.1295	−8.24E‐06	7.55E‐05	0.9154
Fms‐related tyrosine kinase 3 ligand levels	AD	24.1239	0.5122	23.7011	0.4788	2.54E‐05	3.90E‐05	0.5217
Interleukin‐18 levels	AD	10.9330	0.8972	10.8018	0.8667	−2.47E‐05	6.82E‐05	0.7216
Leukaemia inhibitory factor receptor levels	AD	11.7142	0.8171	8.7776	0.9223	0.0001	6.17E‐05	0.1059
Monocyte chemoattractant protein−1 levels	AD	17.3253	0.6912	17.2724	0.6352	‐1.32E‐05	5.74E‐05	0.8204
SIR2‐like protein 2 levels	AD	7.4149	0.7646	6.3462	0.7854	−9.57E‐05	9.26E‐05	0.3256
T‐cell surface glycoprotein CD5 levels	ALS	30.7076	0.0787	25.9753	0.1666	0.0263	0.0138	0.0707
C‐X‐C motif chemokine 10 levels	ALS	17.0058	0.7108	15.6951	0.7353	−0.0093	0.0081	0.2658
C‐X‐C motif chemokine 11 levels	ALS	29.6784	0.1265	28.3504	0.1305	−0.0125	0.0126	0.3326
Hepatocyte growth factor levels	ALS	11.7821	0.6954	11.5643	0.6412	−0.0067	0.0143	0.6479
Interleukin‐2 receptor subunit beta levels	ALS	7.9490	0.7891	7.9449	0.7182	0.0009	0.0142	0.9505
Transforming growth factor‐alpha levels	ALS	14.1780	0.2895	14.1687	0.2238	0.0016	0.0189	0.9336
Tumour necrosis factor ligand superfamily member 12 levels	ALS	19.0340	0.7955	16.4377	0.8716	−0.0147	0.0091	0.1202
Adenosine Deaminase levels	MS	2.6574	0.8505	2.3681	0.7962	−0.0100	0.0185	0.6138
Natural killer cell receptor 2B4 levels	MS	30.7325	**0.0037**	30.0087	**0.0028**	0.0095	0.0176	0.6004
CD40L receptor levels	MS	12.7307	0.2391	9.7914	0.3676	0.0146	0.0089	0.1346
T‐cell surface glycoprotein CD6 isoform levels	MS	19.6573	**0.0327**	18.8484	**0.0265**	0.0092	0.0148	0.5497
C‐X‐C motif chemokine 10 levels	MS	17.2355	0.1011	17.0338	0.0736	−0.0066	0.0191	0.7379
Leukaemia inhibitory factor receptor levels	MS	22.8680	**0.0289**	20.1170	**0.0438**	−0.0186	0.0152	0.2456
Neurturin levels	MS	0.5972	0.9881	0.4282	0.9801	0.0143	0.0348	0.7020
Fibroblast growth factor 21 levels	PD	19.8321	0.0703	19.8196	**0.0479**	0.0022	0.0265	0.9352
Interleukin‐12 subunit beta levels	PD	22.1606	0.4503	21.1709	0.4486	−0.0119	0.0120	0.3331
Interleukin‐17A levels	PD	4.3082	0.9600	4.2877	0.9334	0.0034	0.0237	0.8889
Neurturin levels	PD	3.8448	0.9541	3.5504	0.9384	0.0114	0.0210	0.6006
Transforming growth factor alpha levels	PD	9.6369	0.5633	9.2925	0.5046	−0.0144	0.0245	0.5704
Tumour necrosis factor receptor superfamily member 9 levels	PD	18.4875	0.6767	18.3989	0.6236	0.0047	0.0158	0.7690

*Note*: Bold indicates *p*‐value <0.05.

Our investigation delved deeper into the implications of our initial findings through the MR‐PRESSO global test (Table 3). The results, meticulously detailed in Table 3, encompass a range of factors including the causal estimates, the observed residual sum of squares (RSS_obs) and the corresponding *p*‐values. It is of particular note that the *p*‐values from the MR‐PRESSO global test, which assess the influence of circulating cytokines on four distinct neurodegenerative diseases, consistently surpassed the significance threshold of 0.05. An exception to this was observed with the cytokine Natural killer cell receptor 2B4 in its relation to MS, where the *p*‐value registered at 0.0170, thereby undermining the strength of the causative links unearthed.

**TABLE 3:** MR analysis of the causal relationship between circulating inflammatory cytokines and AD, ALS, MS and PD.

Exposure	Outcome	MR‐PRESSO global test				Steiger test
		MR analysis	Causal estimate	RSSobs	*p*‐value	*p*‐value
C‐C motif chemokine 19 levels	AD	Raw	0.0003	27.0010	0.2810	**0.0103**
		Outlier‐corrected	NA	27.0010	0.2810	
C‐X‐C motif chemokine 1 levels	AD	Raw	−0.0007	15.5951	0.3490	**0.0003**
		Outlier‐corrected	NA	15.5951	0.3490	
Fms‐related tyrosine kinase 3 ligand levels	AD	Raw	0.0005	26.2622	0.5850	**0.0013**
		Outlier‐corrected	NA	26.2622	0.5850	
Interleukin‐18 levels	AD	Raw	−0.0006	11.8362	0.9270	**0.0211**
		Outlier‐corrected	NA	11.8362	0.9270	
Leukaemia inhibitory factor receptor levels	AD	Raw	−0.0007	16.7156	0.6880	**0.0045**
		Outlier‐corrected	NA	16.7156	0.6880	
Monocyte chemoattractant protein‐1 levels	AD	Raw	−0.0008	20.1099	0.6830	**0.0226**
		Outlier‐corrected	NA	20.1099	0.6830	
SIR2‐like protein 2 levels	AD	Raw	0.0010	8.6538	0.8010	0.3211
		Outlier‐corrected	NA	8.6538	0.8010	
T‐cell surface glycoprotein CD5 levels	ALS	Raw	−0.1089	37.1059	0.0660	0.1626
		Outlier‐corrected	NA	37.1059	0.0660	
C‐X‐C motif chemokine 10 levels	ALS	Raw	−0.1208	21.0341	0.6130	0.1662
		Outlier‐corrected	NA	21.0341	0.6130	
C‐X‐C motif chemokine 11 levels	ALS	Raw	−0.1016	33.1997	0.0930	0.0872
		Outlier‐corrected	NA	33.1997	0.0930	
Hepatocyte growth factor levels	ALS	Raw	0.1436	16.3208	0.5830	0.3431
		Outlier‐corrected	NA	16.3208	0.5830	
Interleukin‐2 receptor subunit beta levels	ALS	Raw	−0.1143	9.9155	0.8020	0.5187
		Outlier‐corrected	NA	9.9155	0.8020	
Transforming growth factor‐alpha levels	ALS	Raw	−0.1474	17.0956	0.2900	0.5049
		Outlier‐corrected	NA	17.0956	0.2900	
Tumour necrosis factor ligand superfamily member 12 levels	ALS	Raw	0.0662	28.8398	0.4900	**0.0349**
		Outlier‐corrected	NA	28.8398	0.4900	
Adenosine Deaminase levels	MS	Raw	−0.2646	4.8007	0.7790	0.2334
		Outlier‐corrected	NA	4.8007	0.7790	
Natural killer cell receptor 2B4 levels	MS	Raw	0.1899	42.1092	**0.0170**	**0.0227**
		Outlier‐corrected	0.2123	42.1092	**0.0170**	
CD40L receptor levels	MS	Raw	−0.2566	39.5068	0.3420	**5.00E‐16**
		Outlier‐corrected	NA	39.5068	0.3420	
T‐cell surface glycoprotein CD6 isoform levels	MS	Raw	0.1292	36.2046	0.3490	**1.34E‐39**
		Outlier‐corrected	NA	36.2046	0.3490	
C‐X‐C motif chemokine 10 levels	MS	Raw	0.2410	24.5560	0.0870	0.3381
		Outlier‐corrected	NA	24.5560	0.0870	
Leukaemia inhibitory factor receptor levels	MS	Raw	0.1671	25.9150	0.0700	**0.0054**
		Outlier‐corrected	NA	25.9150	0.0700	
Neurturin levels	MS	Raw	−0.3566	0.8597	0.9910	0.6796
		Outlier‐corrected	NA	0.8597	0.9910	
Fibroblast growth factor 21 levels	PD	Raw	−0.2022	28.7062	0.0830	0.2562
		Outlier‐corrected	NA	28.7062	0.0830	
Interleukin‐12 subunit beta levels	PD	Raw	−0.1068	24.4464	0.4110	**0.0004**
		Outlier‐corrected	NA	24.4464	0.4110	
Interleukin‐17A levels	PD	Raw	0.2509	5.1937	0.9630	0.5338
		Outlier‐corrected	NA	5.1937	0.9630	
Neurturin levels	PD	Raw	−0.3084	4.7136	0.9530	0.5772
		Outlier‐corrected	NA	4.7136	0.9530	
Transforming growth factor alpha levels	PD	Raw	−0.1918	11.8116	0.5540	0.4963
		Outlier‐corrected	NA	11.8116	0.5540	
Tumour necrosis factor receptor superfamily member 9 levels	PD	Raw	−0.1683	19.9827	0.7010	0.2389
		Outlier‐corrected	NA	19.9827	0.7010	

*Note*: Bold indicates *p*‐value <0.05.

The implementation of the Steiger test was pivotal in determining the direction of causality between the cytokines and the neurodegenerative diseases. The findings substantiated those six cytokines—namely, C‐C motif chemokine 19, C‐X‐C motif chemokine 1, Fms‐related tyrosine kinase 3 ligand, Interleukin‐18, Leukaemia inhibitory factor receptor and Monocyte chemoattractant protein‐1—were indeed instigating an upstream influence on AD pathogenesis, with *p*‐values falling below the 0.05 threshold as depicted in Table 3. In the case of ALS, Tumour necrosis factor ligand superfamily member 12 stood out as the sole cytokine exerting a similar upstream effect, with a *p*‐value of 0.0349 (Table 3). Regarding MS, four cytokines, including Natural killer cell receptor 2B4 (*p*‐value = 0.0227), CD40L receptor (*p*‐value = 5.00E‐16), T‐cell surface glycoprotein CD6 isoform (1.34E‐39) and Leukaemia inhibitory factor receptor (*p*‐value = 0.0054), were identified as having an upstream impact on its pathogenesis (Table 3). For PD, Interleukin‐12 subunit beta (*p* = 0.0004) was the only cytokine implicated in exerting an upstream effect (Table 3). These insights suggest that the cytokines in question might precede and potentially precipitate the onset of these conditions.

On the contrary, the Steiger test did not reveal a statistically significant directional causality for SIR2‐like protein 2 in relation to AD, nor did it do so for other six cytokines with respect to ALS, three cytokines in the context of MS and five cytokines concerning PD. This lack of statistical significance suggests that the evidence currently available does not substantiate a clear‐cut causal nexus within the pathogenesis of the aforementioned neurodegenerative diseases for these cytokines (All *p* > 0.05, Table 3). Consequently, further investigative efforts are warranted to elucidate their potential roles and impacts.

**FIGURE 2:**
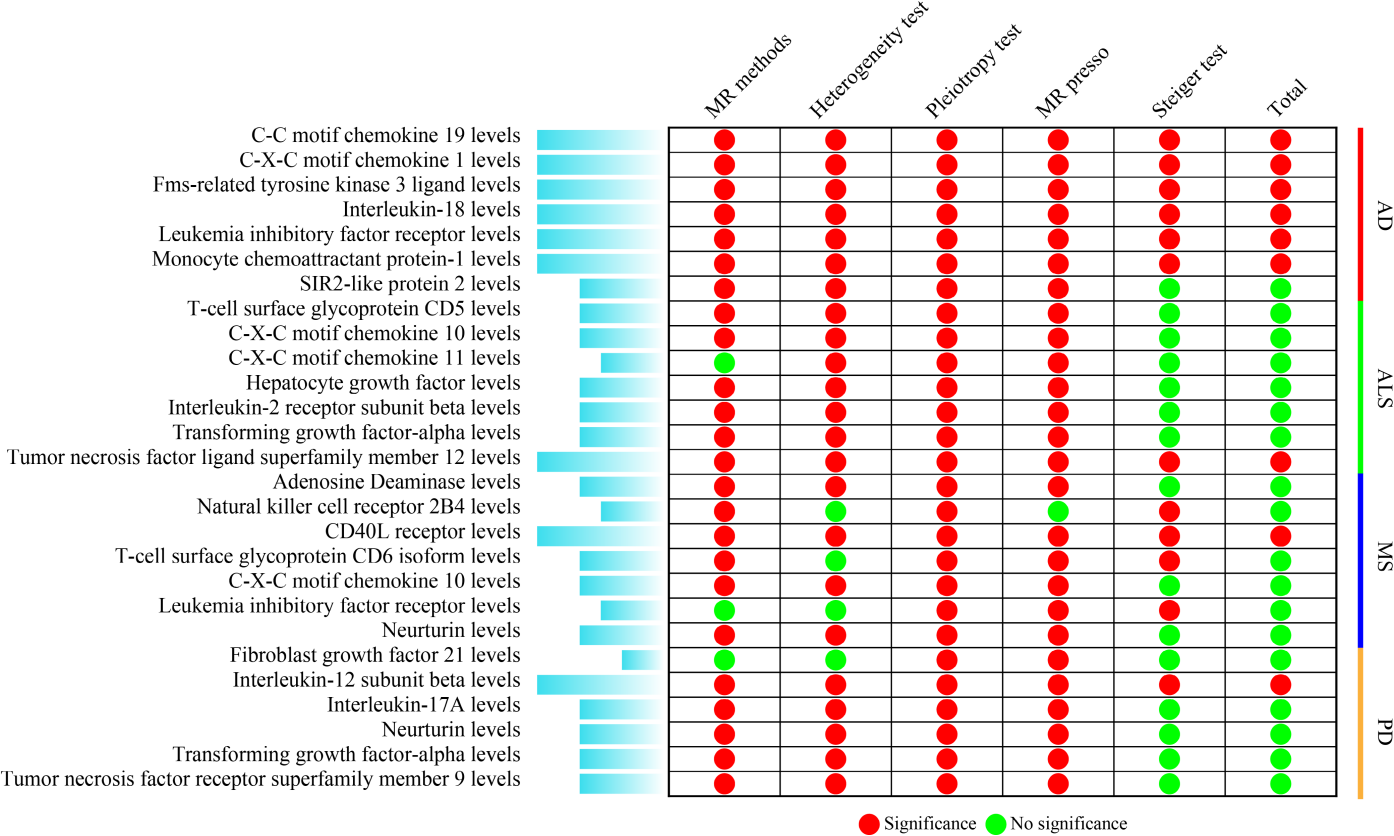
Summary of MR analysis.

Collectively, utilizing a variety of MR methods, we have validated a substantial causality between circulating inflammatory cytokines and four neurodegenerative conditions (Figure 2). This includes the identification of six cytokines (C‐C motif chemokine 19, C‐X‐C motif chemokine 1, Fms‐related tyrosine kinase 3 ligand, Interleukin‐18, Leukaemia inhibitory factor receptor and Monocyte chemoattractant protein‐1) associated with AD, Tumour necrosis factor ligand superfamily member 12 linked to ALS, CD40L receptor influencing MS and Interleukin‐12 subunit beta impacting PD (Figure 2). Additionally, the reliability of the causal assessments for these circulating cytokines in relation to AD, ALS, MS and PD was further confirmed through leave‐one‐out sensitivity analyses, consistently validating the integrity of the observed causal connections (Figure S1—Data S1).

### 3.3 Druggability evaluating the potential of therapeutic targets

In the druggability evaluation, we found that eight identified proteins have been targeted for drug development (Table S4—Data S1). Drugs targeting and inhibiting C‐X‐C motif chemokine 1 have been investigated (WWL123, WWL70, KT‐109 and ABX‐1431). Some drugs targeting Fms‐related tyrosine kinase 3 ligand have been approved as antineoplastic agents (for example, Quizartinib, Sorafenib and Ponatinib), immunosuppressant (Everolimus) and antiinflammatory agent (Ruxolitinib). Drug (Interferon alfa‐2b, colchicine, Peginterferon alfa‐2a, Anhydrous tacrolimus and Mycophenolate) targeting Interleukin‐18 has been approved for the treating hepatitis B, hepatitis C, gout, tumour and immunomodulatory. Drug (Rosiglitazone) targeting CD40L receptor has been approved to treat AD and diabetes. Drug (Briakinumab) targeting Interleukin‐12 subunit beta have been investigated for treatment of MS.

## 4 DISCUSSION

In this present study, we first harnessed the robust analytical capabilities of the two‐sample MR method to dissect the potential causal interplay between the circulating levels of 91 inflammatory proteins and the risk of four neurodegenerative diseases. Our MR analysis provides novel insights into the causal relationships between inflammatory cytokines and the risk of AD, ALS, MS and PD. The findings suggest a complex interplay where certain cytokines appear to modulate disease susceptibility differentially.

The activation of neuroinflammation in reaction to pathological insults acts as a defence mechanism, initially intended to safeguard the brain by eliminating or suppressing pathogens and facilitating tissue repair in order to restore balance.^33^ Previous analyses have pointed out the effects of 34 circulating cytokine concentrations on the risk of AD and three cognitive domains.^34^ In our study, we analysed the causal relationship between 91 circulating inflammatory cytokines and AD to gain a more comprehensive understanding of the impact of neuroinflammation on the risk of AD occurrence. For AD, the attenuation of risk associated with specific cytokines such as C‐X‐C motif chemokine 1, Interleukin‐18, Leukaemia inhibitory factor receptor and Monocyte chemoattractant protein‐1 indicates a potential neuroprotective effect. These cytokines may be involved in processes that mitigate the pathological cascade leading to AD, such as the modulation of amyloid‐beta clearance or the regulation of synaptic plasticity. There is currently evidence to suggest that these inflammatory factors may play a potential role in AD. More specifically, previous studies have shown elevated CXCL1 expression in the brain,^35^ cerebrospinal fluid^36^ and blood monocytes^37^ of individuals with AD. The CXCL1/CXCR2 axis potentially facilitates the migration of monocytes across the endothelium into the brain, where they differentiate into microglia and contribute to the elimination of Aβ plaque deposition, thus participating in the mechanism of inhibiting the progression of AD.^38^ However, the increased expression of CXCL1 in the brain of AD patients may bind to CXCR2 receptors on neurons, leading to Tau hyperphosphorylation and the formation of neurofibrillary tangles.^39^ The activation of CXCR2 is more conducive to further promoting the release of Aβ.^40^ Conversely, the positive causal relationship observed with C‐C motif chemokine 19 and Fms‐related tyrosine kinase 3 ligand suggests that these cytokines might contribute to the inflammatory milieu that exacerbates AD pathology. Previous studies have indicated that the higher level of CCL19 protein was related with worse executive function and language domain factor scores.^41^ We believe that further research is needed to better understand the role of the inflammatory factors in the aetiology of AD.

In the context of ALS, MS and PD, our results point to an inverse association with transforming growth factor‐alpha, CD40L receptor and Interleukin‐12 subunit beta, respectively. These findings may reflect a counter‐regulatory response within the neuroinflammatory environment of these diseases. For instance, the lower levels of transforming growth factor‐alpha might suggest a diminished neuroprotective response in ALS, while the decreased activity of the CD40L receptor and Interleukin‐12 subunit beta could indicate alterations in immune cell signalling and cytokine networks in MS and PD. The absence of horizontal pleiotropy, as evidenced by the consistency across various MR analyses and sensitivity checks, strengthens the validity of our causal inferences. This suggests that the genetic instruments used for the MR analysis are specific to the cytokines of interest and are not influenced by confounding pathways.

Our findings have several potential clinical implications. The protective effects of cytokines such as C‐X‐C motif chemokine 1 and Interleukin‐18 in AD suggest that these could be potential targets for neuroprotective therapies. Conversely, the risk‐increasing effects of cytokines like C‐C motif chemokine 19 in AD indicate that inhibition of these cytokines might be a strategy for disease prevention or progression slowdown. Moreover, the causal relationships uncovered in this study could inform the development of biomarkers for early disease detection and prognosis. For example, monitoring levels of Tumour necrosis factor ligand superfamily member 12 might aid in assessing ALS risk, while CD40L receptor levels could be indicative of MS susceptibility. In the context of personalized medicine, understanding an individual's genetic predisposition to produce certain levels of these cytokines could help in stratifying patients for targeted interventions or in predicting disease risk. This could lead to more tailored preventive strategies and treatment approaches in neurodegenerative diseases.

While our study provides valuable insights, it is important to acknowledge its limitations. Firstly, the MR approach assumes a linear relationship between genetic variants, cytokine levels and disease risk, which may not always hold true in complex biological systems. Secondly, our analysis is based on European populations and the results may not be generalizable to other ethnic groups due to potential differences in genetic architecture and environmental factors. Furthermore, while we identified causal relationships, the exact biological mechanisms underlying these associations require further experimental validation. Lastly, our study does not account for potential gene–environment interactions, which could modify the effects of these cytokines on neurodegenerative diseases. Despite these limitations, our findings provide a robust foundation for future research and highlight the potential of targeting specific inflammatory pathways in the prevention and treatment of neurodegenerative diseases.

In conclusion, our study elucidates the genetically determined influence of inflammatory cytokines on the aetiology of NDs, underscoring the intricate role of the immune system in these conditions. These findings pave the way for future research into targeted therapeutic strategies that modulate cytokine activity, with the potential to alter the course of these debilitating diseases.

## AUTHOR CONTRIBUTIONS

**Wenwen Lin:** Conceptualization (equal); data curation (equal); formal analysis (equal); methodology (equal); writing – original draft (equal); writing – review and editing (equal). **Xuewei Wu:** Data curation (equal); formal analysis (equal); investigation (equal); methodology (equal); writing – original draft (equal); writing – review and editing (equal). **Guanyong Ou:** Conceptualization (equal); formal analysis (equal); investigation (equal); methodology (equal); visualization (equal); writing – original draft (equal); writing – review and editing (equal).

## FUNDING INFORMATION

The present study was supported by the Pathology Department Key Discipline Construction within the institution in Shenzhen Hospital of Southern Medical University [grant number: ZCXM‐2024‐XZ‐0002‐004].

## CONFLICT OF INTEREST STATEMENT

The authors declare no conflicts of interest.

## Supporting information


Data S1.

## Data Availability

All data are available in the main text or the supplementary materials—Data S1.
