# Supplementary material for: Causal association of circulating inflammatory proteins on neurodegenerative diseases: Insights from a mendelian randomization study
Source: J Cell Mol Med. 2024 Oct 29;28(20):e70176. doi: 10.1111/jcmm.70176 (PMC11520441; doi:10.1111/jcmm.70176)
Supplement: Supplementary file 1 — Data S1. [file JCMM-28-e70176-s001.docx]

Supplementary Materials for

**Causal Association of Circulating Inflammatory Cytokine Levels on Neurodegenerative Diseases: Insights from a Mendelian Randomization Study**

Wenen Lin, Xuewei Wu and Guanyong Ou*.*

*Corresponding author. Wenwen Lin, [15wwlin@alumni.stu.edu.cn](mailto:15wwlin@stu.edu.cn); Guanyong Ou, [guanyongou@126.com](mailto:guanyongou@126.com)

**The Supplementary Materials includes:**

**Figure S1.** Leave-one-out plot of circulating cytokines on AD, ALS, MS, and PD.

**Table S1.** Primary results for MR analysis of circulating inflammatory cytokines on AD, ALS, MS, and PD.

**Table S2.** Different MR analysis of circulating inflammatory cytokines on AD, ALS, MS, and PD.

**Table S3.** Characteristics of the genetic instrument variables for selected cytokines at the genome-wide significance level for AD, ALS, MS, and PD (P < 5 × 10^–6^).

**Table S4.** Druggability of proteins potentially causally associated with Neurodegenerative Diseases.

**
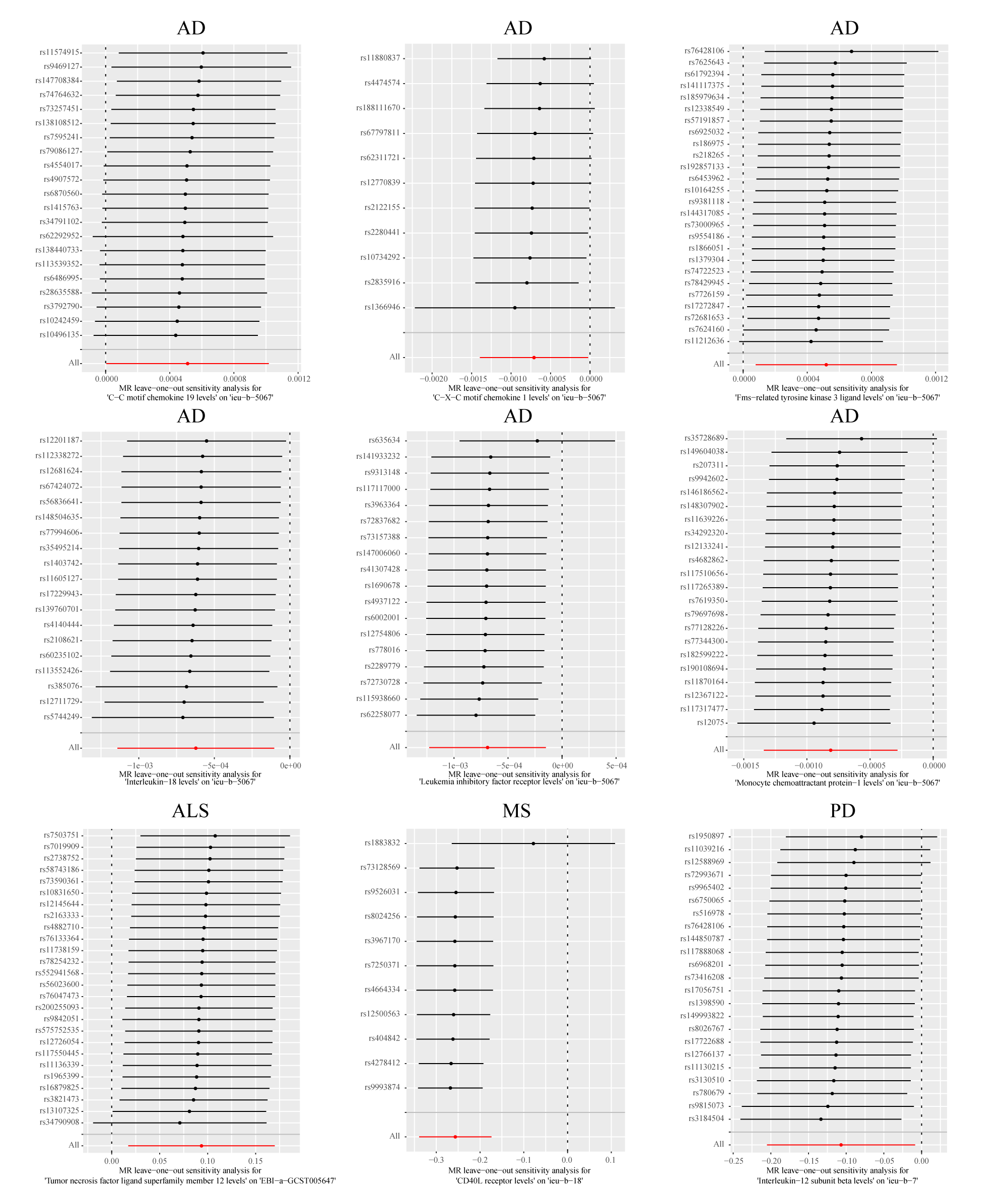
**

**Figure S1.** Primary results for MR analysis of circulating inflammatory cytokines on AD, ALS, MS, and PD.

**Table S1.** Primary results for MR analysis of circulating inflammatory cytokines on AD, ALS, MS, and PD.

| **Exposure** | **Method** | **nSNP** | **SE** | **OR** | **OR_lci95** | **OR_uci95** | ***P*-value** | **Outcome** |
| --- | --- | --- | --- | --- | --- | --- | --- | --- |
| Adenosine Deaminase levels | IVW | 15 | 0.0002 | 0.9999 | 0.9996 | 1.0002 | 0.6050 | AD |
| Artemin levels | IVW | 18 | 0.0003 | 1.0002 | 0.9995 | 1.0009 | 0.5253 | AD |
| Axin-1 levels | IVW | 7 | 0.0006 | 1.0006 | 0.9994 | 1.0018 | 0.3283 | AD |
| beta-nerve growth factor levels | IVW | 20 | 0.0004 | 1.0004 | 0.9997 | 1.0012 | 0.2134 | AD |
| Caspase 8 levels | IVW | 11 | 0.0004 | 1.0003 | 0.9995 | 1.0011 | 0.4506 | AD |
| **C-C motif chemokine 19 levels** | **IVW** | **21** | **0.0003** | **1.0005** | **1.0000** | **1.0010** | **0.0478** | **AD** |
| C-C motif chemokine 20 levels | IVW | 19 | 0.0003 | 0.9994 | 0.9988 | 1.0001 | 0.0781 | AD |
| C-C motif chemokine 23 levels | IVW | 24 | 0.0009 | 1.0002 | 0.9985 | 1.0019 | 0.8120 | AD |
| C-C motif chemokine 25 levels | IVW | 29 | 0.0002 | 1.0000 | 0.9997 | 1.0003 | 0.7524 | AD |
| C-C motif chemokine 28 levels | IVW | 18 | 0.0004 | 0.9994 | 0.9987 | 1.0001 | 0.0889 | AD |
| C-C motif chemokine 4 levels | IVW | 18 | 0.0002 | 1.0001 | 0.9997 | 1.0004 | 0.6843 | AD |
| CD40L receptor levels | IVW | 15 | 0.0002 | 1.0001 | 0.9997 | 1.0005 | 0.6766 | AD |
| CUB domain-containing protein 1 levels | IVW | 23 | 0.0002 | 1.0003 | 0.9998 | 1.0007 | 0.2706 | AD |
| **C-X-C motif chemokine 1 levels** | **IVW** | **11** | **0.0003** | **0.9993** | **0.9986** | **1.0000** | **0.0424** | **AD** |
| C-X-C motif chemokine 10 levels | IVW | 17 | 0.0003 | 1.0000 | 0.9994 | 1.0006 | 0.9750 | AD |
| C-X-C motif chemokine 11 levels | IVW | 19 | 0.0004 | 1.0000 | 0.9993 | 1.0007 | 0.9378 | AD |
| C-X-C motif chemokine 5 levels | IVW | 13 | 0.0002 | 1.0002 | 0.9998 | 1.0006 | 0.3157 | AD |
| C-X-C motif chemokine 6 levels | IVW | 10 | 0.0003 | 1.0003 | 0.9998 | 1.0008 | 0.2857 | AD |
| C-X-C motif chemokine 9 levels | IVW | 18 | 0.0004 | 0.9995 | 0.9987 | 1.0004 | 0.2533 | AD |
| Cystatin D levels | IVW | 31 | 0.0001 | 0.9998 | 0.9995 | 1.0001 | 0.1633 | AD |
| Delta and Notch-like epidermal growth factor-related receptor levels | IVW | 16 | 0.0003 | 1.0001 | 0.9995 | 1.0008 | 0.6328 | AD |
| Eotaxin levels | IVW | 17 | 0.0003 | 0.9997 | 0.9991 | 1.0003 | 0.3807 | AD |
| Eukaryotic translation initiation factor 4E-binding protein 1 levels | IVW | 11 | 0.0004 | 1.0001 | 0.9993 | 1.0009 | 0.8117 | AD |
| Fibroblast growth factor 19 levels | IVW | 20 | 0.0003 | 0.9999 | 0.9994 | 1.0005 | 0.8457 | AD |
| Fibroblast growth factor 21 levels | IVW | 13 | 0.0004 | 1.0001 | 0.9994 | 1.0008 | 0.8433 | AD |
| Fibroblast growth factor 23 levels | IVW | 13 | 0.0005 | 1.0005 | 0.9997 | 1.0014 | 0.2272 | AD |
| Fibroblast growth factor 5 levels | IVW | 23 | 0.0002 | 1.0001 | 0.9997 | 1.0005 | 0.6224 | AD |
| **Fms-related tyrosine kinase 3 ligand levels** | **IVW** | **26** | **0.0002** | **1.0005** | **1.0001** | **1.0010** | **0.0210** | **AD** |
| Fractalkine levels | IVW | 20 | 0.0004 | 1.0000 | 0.9992 | 1.0008 | 0.9701 | AD |
| Glial cell line-derived neurotrophic factor levels | IVW | 14 | 0.0003 | 1.0002 | 0.9997 | 1.0008 | 0.4624 | AD |
| Hepatocyte growth factor levels | IVW | 17 | 0.0004 | 1.0000 | 0.9992 | 1.0008 | 0.9829 | AD |
| Interferon gamma levels | IVW | 9 | 0.0006 | 1.0004 | 0.9992 | 1.0016 | 0.5240 | AD |
| Interleukin-10 levels | IVW | 19 | 0.0003 | 1.0005 | 0.9999 | 1.0011 | 0.1309 | AD |
| Interleukin-10 receptor subunit alpha levels | IVW | 10 | 0.0005 | 1.0001 | 0.9992 | 1.0010 | 0.8326 | AD |
| Interleukin-10 receptor subunit beta levels | IVW | 17 | 0.0002 | 1.0000 | 0.9996 | 1.0004 | 0.9107 | AD |
| Interleukin-12 subunit beta levels | IVW | 25 | 0.0002 | 1.0002 | 0.9998 | 1.0006 | 0.3036 | AD |
| Interleukin-13 levels | IVW | 12 | 0.0005 | 0.9992 | 0.9982 | 1.0002 | 0.1309 | AD |
| Interleukin-15 receptor subunit alpha levels | IVW | 12 | 0.0002 | 1.0000 | 0.9996 | 1.0005 | 0.8775 | AD |
| Interleukin-17A levels | IVW | 14 | 0.0004 | 1.0003 | 0.9995 | 1.0010 | 0.4976 | AD |
| Interleukin-17C levels | IVW | 17 | 0.0003 | 1.0005 | 0.9998 | 1.0012 | 0.1298 | AD |
| **Interleukin-18 levels** | **IVW** | **19** | **0.0003** | **0.9994** | **0.9989** | **0.9999** | **0.0186** | **AD** |
| interleukin-18 receptor 1 levels | IVW | 23 | 0.0001 | 1.0000 | 0.9997 | 1.0002 | 0.7761 | AD |
| Interleukin-1-alpha levels | IVW | 14 | 0.0003 | 0.9998 | 0.9993 | 1.0004 | 0.5305 | AD |
| Interleukin-2 levels | IVW | 16 | 0.0004 | 0.9999 | 0.9992 | 1.0007 | 0.8440 | AD |
| Interleukin-2 receptor subunit beta levels | IVW | 12 | 0.0005 | 0.9994 | 0.9983 | 1.0005 | 0.2645 | AD |
| Interleukin-20 levels | IVW | 10 | 0.0005 | 0.9995 | 0.9986 | 1.0004 | 0.2965 | AD |
| Interleukin-20 receptor subunit alpha levels | IVW | 8 | 0.0005 | 0.9995 | 0.9986 | 1.0005 | 0.3417 | AD |
| Interleukin-22 receptor subunit alpha-1 levels | IVW | 9 | 0.0006 | 0.9994 | 0.9982 | 1.0005 | 0.2814 | AD |
| Interleukin-24 levels | IVW | 9 | 0.0005 | 1.0000 | 0.9991 | 1.0009 | 0.9345 | AD |
| Interleukin-33 levels | IVW | 9 | 0.0005 | 0.9998 | 0.9988 | 1.0007 | 0.6480 | AD |
| Interleukin-4 levels | IVW | 11 | 0.0004 | 1.0006 | 0.9998 | 1.0014 | 0.1211 | AD |
| Interleukin-5 levels | IVW | 9 | 0.0006 | 1.0004 | 0.9992 | 1.0015 | 0.5218 | AD |
| Interleukin-6 levels | IVW | 8 | 0.0006 | 1.0003 | 0.9992 | 1.0014 | 0.6367 | AD |
| Interleukin-7 levels | IVW | 9 | 0.0005 | 1.0000 | 0.9989 | 1.0010 | 0.9865 | AD |
| Interleukin-8 levels | IVW | 16 | 0.0004 | 0.9997 | 0.9990 | 1.0005 | 0.4834 | AD |
| Latency-associated peptide transforming growth factor beta 1 levels | IVW | 19 | 0.0003 | 1.0000 | 0.9993 | 1.0006 | 0.9141 | AD |
| Leukemia inhibitory factor levels | IVW | 11 | 0.0005 | 1.0000 | 0.9990 | 1.0010 | 0.9541 | AD |
| **Leukemia inhibitory factor receptor levels** | **IVW** | **18** | **0.0003** | **0.9993** | **0.9988** | **0.9999** | **0.0122** | **AD** |
| Macrophage colony-stimulating factor 1 levels | IVW | 17 | 0.0005 | 1.0004 | 0.9995 | 1.0013 | 0.3806 | AD |
| Macrophage inflammatory protein 1a levels | IVW | 13 | 0.0002 | 1.0000 | 0.9995 | 1.0004 | 0.9394 | AD |
| Matrix metalloproteinase-1 levels | IVW | 15 | 0.0003 | 0.9998 | 0.9992 | 1.0005 | 0.5851 | AD |
| Matrix metalloproteinase-10 levels | IVW | 16 | 0.0002 | 1.0000 | 0.9995 | 1.0004 | 0.8696 | AD |
| Monocyte chemoattractant protein 2 levels | IVW | 22 | 0.0001 | 0.9999 | 0.9997 | 1.0002 | 0.5898 | AD |
| **Monocyte chemoattractant protein-1 levels** | **IVW** | **22** | **0.0003** | **0.9992** | **0.9987** | **0.9997** | **0.0026** | **AD** |
| Monocyte chemoattractant protein-3 levels | IVW | 17 | 0.0003 | 0.9998 | 0.9993 | 1.0004 | 0.6085 | AD |
| Monocyte chemoattractant protein-4 levels | IVW | 20 | 0.0002 | 1.0000 | 0.9996 | 1.0004 | 0.9765 | AD |
| Natural killer cell receptor 2B4 levels | IVW | 20 | 0.0003 | 1.0002 | 0.9996 | 1.0008 | 0.5519 | AD |
| Neurotrophin-3 levels | IVW | 17 | 0.0004 | 0.9995 | 0.9987 | 1.0003 | 0.1903 | AD |
| Neurturin levels | IVW | 13 | 0.0004 | 1.0005 | 0.9998 | 1.0013 | 0.1608 | AD |
| Oncostatin-M levels | IVW | 14 | 0.0004 | 0.9996 | 0.9988 | 1.0005 | 0.3921 | AD |
| Osteoprotegerin levels | IVW | 19 | 0.0003 | 1.0002 | 0.9996 | 1.0008 | 0.4880 | AD |
| Programmed cell death 1 ligand 1 levels | IVW | 16 | 0.0004 | 1.0005 | 0.9998 | 1.0012 | 0.1475 | AD |
| Protein S100-A12 levels | IVW | 14 | 0.0004 | 0.9997 | 0.9989 | 1.0005 | 0.4391 | AD |
| Signaling lymphocytic activation molecule levels | IVW | 23 | 0.0003 | 1.0004 | 0.9998 | 1.0010 | 0.1652 | AD |
| **SIR2-like protein 2 levels** | **IVW** | **12** | **0.0004** | **1.0010** | **1.0002** | **1.0018** | **0.0155** | **AD** |
| STAM binding protein levels | IVW | 11 | 0.0005 | 1.0001 | 0.9991 | 1.0010 | 0.8512 | AD |
| Stem cell factor levels | IVW | 30 | 0.0002 | 0.9999 | 0.9994 | 1.0003 | 0.5398 | AD |
| Sulfotransferase 1A1 levels | IVW | 21 | 0.0003 | 1.0001 | 0.9995 | 1.0008 | 0.7056 | AD |
| T-cell surface glycoprotein CD5 levels | IVW | 22 | 0.0003 | 1.0003 | 0.9998 | 1.0009 | 0.2275 | AD |
| T-cell surface glycoprotein CD6 isoform levels | IVW | 12 | 0.0002 | 1.0000 | 0.9997 | 1.0004 | 0.7677 | AD |
| Thymic stromal lymphopoietin levels | IVW | 13 | 0.0004 | 1.0000 | 0.9992 | 1.0008 | 0.9268 | AD |
| TNF-beta levels | IVW | 23 | 0.0001 | 1.0000 | 0.9998 | 1.0002 | 0.9949 | AD |
| TNF-related activation-induced cytokine levels | IVW | 26 | 0.0003 | 0.9997 | 0.9992 | 1.0002 | 0.2517 | AD |
| TNF-related apoptosis-inducing ligand levels | IVW | 25 | 0.0002 | 0.9998 | 0.9994 | 1.0002 | 0.3755 | AD |
| Transforming growth factor-alpha levels | IVW | 13 | 0.0006 | 1.0001 | 0.9990 | 1.0012 | 0.8494 | AD |
| Tumor necrosis factor levels | IVW | 17 | 0.0003 | 1.0004 | 0.9997 | 1.0010 | 0.2882 | AD |
| Tumor necrosis factor ligand superfamily member 12 levels | IVW | 26 | 0.0003 | 0.9995 | 0.9989 | 1.0000 | 0.0698 | AD |
| Tumor necrosis factor ligand superfamily member 14 levels | IVW | 25 | 0.0002 | 1.0000 | 0.9995 | 1.0004 | 0.9112 | AD |
| Tumor necrosis factor receptor superfamily member 9 levels | IVW | 26 | 0.0003 | 0.9998 | 0.9993 | 1.0003 | 0.4871 | AD |
| Urokinase-type plasminogen activator levels | IVW | 21 | 0.0003 | 0.9996 | 0.9990 | 1.0001 | 0.1360 | AD |
| Vascular endothelial growth factor A levels | IVW | 20 | 0.0003 | 0.9998 | 0.9992 | 1.0004 | 0.5423 | AD |
| Adenosine Deaminase levels | IVW | 13 | 0.0310 | 1.0401 | 0.9788 | 1.1052 | 0.2045 | ALS |
| Artemin levels | IVW | 18 | 0.0636 | 0.9822 | 0.8670 | 1.1126 | 0.7773 | ALS |
| Axin-1 levels | IVW | 8 | 0.0762 | 0.9858 | 0.8491 | 1.1446 | 0.8515 | ALS |
| beta-nerve growth factor levels | IVW | 22 | 0.0529 | 1.0867 | 0.9797 | 1.2055 | 0.1159 | ALS |
| Caspase 8 levels | IVW | 12 | 0.0601 | 1.0009 | 0.8896 | 1.1261 | 0.9882 | ALS |
| C-C motif chemokine 19 levels | IVW | 20 | 0.0507 | 1.0391 | 0.9408 | 1.1477 | 0.4490 | ALS |
| C-C motif chemokine 20 levels | IVW | 22 | 0.0573 | 1.0445 | 0.9335 | 1.1688 | 0.4473 | ALS |
| C-C motif chemokine 23 levels | IVW | 22 | 0.0523 | 1.0022 | 0.9045 | 1.1104 | 0.9670 | ALS |
| C-C motif chemokine 25 levels | IVW | 24 | 0.0212 | 0.9902 | 0.9499 | 1.0322 | 0.6415 | ALS |
| C-C motif chemokine 28 levels | IVW | 23 | 0.0547 | 1.0431 | 0.9370 | 1.1612 | 0.4410 | ALS |
| C-C motif chemokine 4 levels | IVW | 19 | 0.0332 | 0.9998 | 0.9368 | 1.0669 | 0.9942 | ALS |
| CD40L receptor levels | IVW | 18 | 0.0299 | 0.9886 | 0.9323 | 1.0483 | 0.7018 | ALS |
| CUB domain-containing protein 1 levels | IVW | 24 | 0.0371 | 0.9926 | 0.9230 | 1.0674 | 0.8410 | ALS |
| C-X-C motif chemokine 1 levels | IVW | 11 | 0.0482 | 0.9676 | 0.8804 | 1.0636 | 0.4952 | ALS |
| **C-X-C motif chemokine 10 levels** | **IVW** | **22** | **0.0448** | **0.8862** | **0.8116** | **0.9676** | **0.0071** | **ALS** |
| **C-X-C motif chemokine 11 levels** | **IVW** | **23** | **0.0473** | **0.9034** | **0.8234** | **0.9911** | **0.0316** | **ALS** |
| C-X-C motif chemokine 5 levels | IVW | 15 | 0.0415 | 1.0548 | 0.9723 | 1.1443 | 0.1991 | ALS |
| C-X-C motif chemokine 6 levels | IVW | 14 | 0.0391 | 1.0157 | 0.9409 | 1.0965 | 0.6894 | ALS |
| C-X-C motif chemokine 9 levels | IVW | 23 | 0.0478 | 0.9175 | 0.8354 | 1.0077 | 0.0719 | ALS |
| Cystatin D levels | IVW | 31 | 0.0232 | 1.0055 | 0.9608 | 1.0522 | 0.8139 | ALS |
| Delta and Notch-like epidermal growth factor-related receptor levels | IVW | 16 | 0.0581 | 1.0437 | 0.9314 | 1.1695 | 0.4616 | ALS |
| Eotaxin levels | IVW | 20 | 0.0479 | 1.0239 | 0.9321 | 1.1247 | 0.6220 | ALS |
| Eukaryotic translation initiation factor 4E-binding protein 1 levels | IVW | 12 | 0.0742 | 0.9676 | 0.8366 | 1.1191 | 0.6576 | ALS |
| Fibroblast growth factor 19 levels | IVW | 20 | 0.0447 | 0.9921 | 0.9089 | 1.0830 | 0.8596 | ALS |
| Fibroblast growth factor 21 levels | IVW | 13 | 0.0484 | 0.9562 | 0.8695 | 1.0514 | 0.3549 | ALS |
| Fibroblast growth factor 23 levels | IVW | 16 | 0.0575 | 1.0157 | 0.9074 | 1.1368 | 0.7871 | ALS |
| Fibroblast growth factor 5 levels | IVW | 23 | 0.0252 | 1.0019 | 0.9536 | 1.0526 | 0.9399 | ALS |
| Fms-related tyrosine kinase 3 ligand levels | IVW | 29 | 0.0347 | 1.0081 | 0.9417 | 1.0791 | 0.8168 | ALS |
| Fractalkine levels | IVW | 21 | 0.0588 | 0.9678 | 0.8624 | 1.0861 | 0.5778 | ALS |
| Glial cell line-derived neurotrophic factor levels | IVW | 14 | 0.0691 | 1.0931 | 0.9546 | 1.2516 | 0.1977 | ALS |
| **Hepatocyte growth factor levels** | **IVW** | **16** | **0.0561** | **1.1291** | **1.0116** | **1.2604** | **0.0304** | **ALS** |
| Interferon gamma levels | IVW | 10 | 0.0862 | 1.0512 | 0.8879 | 1.2446 | 0.5621 | ALS |
| Interleukin-10 levels | IVW | 19 | 0.0516 | 0.9693 | 0.8760 | 1.0726 | 0.5464 | ALS |
| Interleukin-10 receptor subunit alpha levels | IVW | 8 | 0.0860 | 1.1824 | 0.9989 | 1.3995 | 0.0515 | ALS |
| Interleukin-10 receptor subunit beta levels | IVW | 18 | 0.0338 | 1.0026 | 0.9383 | 1.0714 | 0.9384 | ALS |
| Interleukin-12 subunit beta levels | IVW | 28 | 0.0254 | 0.9719 | 0.9246 | 1.0216 | 0.2624 | ALS |
| Interleukin-13 levels | IVW | 13 | 0.0636 | 1.0530 | 0.9296 | 1.1928 | 0.4167 | ALS |
| Interleukin-15 receptor subunit alpha levels | IVW | 10 | 0.0699 | 1.0036 | 0.8751 | 1.1510 | 0.9588 | ALS |
| Interleukin-17A levels | IVW | 13 | 0.0636 | 1.0163 | 0.8972 | 1.1512 | 0.7993 | ALS |
| Interleukin-17C levels | IVW | 21 | 0.0475 | 0.9882 | 0.9003 | 1.0846 | 0.8022 | ALS |
| Interleukin-18 levels | IVW | 24 | 0.0398 | 1.0295 | 0.9523 | 1.1129 | 0.4653 | ALS |
| interleukin-18 receptor 1 levels | IVW | 24 | 0.0253 | 0.9918 | 0.9438 | 1.0423 | 0.7456 | ALS |
| Interleukin-1-alpha levels | IVW | 13 | 0.0532 | 0.9522 | 0.8580 | 1.0568 | 0.3571 | ALS |
| Interleukin-2 levels | IVW | 16 | 0.0600 | 1.0167 | 0.9039 | 1.1436 | 0.7826 | ALS |
| **Interleukin-2 receptor subunit beta levels** | **IVW** | **13** | **0.0655** | **0.8788** | **0.7728** | **0.9992** | **0.0486** | **ALS** |
| Interleukin-20 levels | IVW | 12 | 0.0689 | 0.9362 | 0.8180 | 1.0716 | 0.3388 | ALS |
| Interleukin-20 receptor subunit alpha levels | IVW | 11 | 0.0786 | 0.9595 | 0.8225 | 1.1194 | 0.5990 | ALS |
| Interleukin-22 receptor subunit alpha-1 levels | IVW | 11 | 0.0854 | 0.9965 | 0.8428 | 1.1781 | 0.9670 | ALS |
| Interleukin-24 levels | IVW | 12 | 0.0890 | 1.0945 | 0.9194 | 1.3030 | 0.3100 | ALS |
| Interleukin-33 levels | IVW | 11 | 0.0925 | 1.0460 | 0.8725 | 1.2539 | 0.6270 | ALS |
| Interleukin-4 levels | IVW | 13 | 0.0743 | 0.9600 | 0.8300 | 1.1104 | 0.5826 | ALS |
| Interleukin-5 levels | IVW | 13 | 0.0755 | 1.0420 | 0.8986 | 1.2082 | 0.5862 | ALS |
| Interleukin-6 levels | IVW | 8 | 0.0752 | 1.1091 | 0.9572 | 1.2852 | 0.1683 | ALS |
| Interleukin-7 levels | IVW | 16 | 0.0650 | 0.9308 | 0.8195 | 1.0573 | 0.2700 | ALS |
| Interleukin-8 levels | IVW | 15 | 0.0643 | 1.0223 | 0.9012 | 1.1597 | 0.7317 | ALS |
| Latency-associated peptide transforming growth factor beta 1 levels | IVW | 18 | 0.0580 | 0.9959 | 0.8889 | 1.1158 | 0.9435 | ALS |
| Leukemia inhibitory factor levels | IVW | 12 | 0.0682 | 1.0005 | 0.8752 | 1.1437 | 0.9942 | ALS |
| Leukemia inhibitory factor receptor levels | IVW | 17 | 0.0555 | 1.0037 | 0.9002 | 1.1191 | 0.9471 | ALS |
| Macrophage colony-stimulating factor 1 levels | IVW | 18 | 0.0608 | 1.0579 | 0.9391 | 1.1916 | 0.3545 | ALS |
| Macrophage inflammatory protein 1a levels | IVW | 17 | 0.0399 | 0.9581 | 0.8860 | 1.0361 | 0.2836 | ALS |
| Matrix metalloproteinase-1 levels | IVW | 16 | 0.0567 | 1.0597 | 0.9482 | 1.1844 | 0.3067 | ALS |
| Matrix metalloproteinase-10 levels | IVW | 17 | 0.0323 | 0.9543 | 0.8958 | 1.0166 | 0.1473 | ALS |
| Monocyte chemoattractant protein 2 levels | IVW | 21 | 0.0183 | 1.0114 | 0.9758 | 1.0483 | 0.5343 | ALS |
| Monocyte chemoattractant protein-1 levels | IVW | 20 | 0.0408 | 0.9982 | 0.9215 | 1.0812 | 0.9640 | ALS |
| Monocyte chemoattractant protein-3 levels | IVW | 20 | 0.0421 | 1.0083 | 0.9285 | 1.0949 | 0.8441 | ALS |
| Monocyte chemoattractant protein-4 levels | IVW | 18 | 0.0347 | 0.9410 | 0.8792 | 1.0071 | 0.0793 | ALS |
| Natural killer cell receptor 2B4 levels | IVW | 20 | 0.0517 | 0.9901 | 0.8946 | 1.0958 | 0.8480 | ALS |
| Neurotrophin-3 levels | IVW | 17 | 0.0615 | 0.9875 | 0.8754 | 1.1140 | 0.8381 | ALS |
| Neurturin levels | IVW | 12 | 0.0654 | 0.8849 | 0.7784 | 1.0059 | 0.0616 | ALS |
| Oncostatin-M levels | IVW | 17 | 0.0735 | 0.9647 | 0.8353 | 1.1141 | 0.6244 | ALS |
| Osteoprotegerin levels | IVW | 20 | 0.0460 | 0.9743 | 0.8904 | 1.0661 | 0.5708 | ALS |
| Programmed cell death 1 ligand 1 levels | IVW | 18 | 0.0730 | 0.9615 | 0.8334 | 1.1093 | 0.5904 | ALS |
| Protein S100-A12 levels | IVW | 15 | 0.0604 | 0.9236 | 0.8205 | 1.0398 | 0.1887 | ALS |
| Signaling lymphocytic activation molecule levels | IVW | 27 | 0.0489 | 0.9797 | 0.8902 | 1.0782 | 0.6746 | ALS |
| SIR2-like protein 2 levels | IVW | 11 | 0.0786 | 0.9376 | 0.8038 | 1.0937 | 0.4120 | ALS |
| STAM binding protein levels | IVW | 14 | 0.0699 | 0.9264 | 0.8078 | 1.0625 | 0.2743 | ALS |
| Stem cell factor levels | IVW | 31 | 0.0325 | 0.9594 | 0.9002 | 1.0225 | 0.2021 | ALS |
| Sulfotransferase 1A1 levels | IVW | 23 | 0.0369 | 1.0421 | 0.9694 | 1.1203 | 0.2632 | ALS |
| **T-cell surface glycoprotein CD5 levels** | **IVW** | **22** | **0.0514** | **0.8885** | **0.8033** | **0.9828** | **0.0216** | **ALS** |
| T-cell surface glycoprotein CD6 isoform levels | IVW | 17 | 0.0323 | 1.0150 | 0.9528 | 1.0814 | 0.6440 | ALS |
| Thymic stromal lymphopoietin levels | IVW | 15 | 0.0692 | 0.9403 | 0.8211 | 1.0769 | 0.3739 | ALS |
| TNF-beta levels | IVW | 23 | 0.0179 | 0.9686 | 0.9351 | 1.0032 | 0.0747 | ALS |
| TNF-related activation-induced cytokine levels | IVW | 30 | 0.0345 | 1.0124 | 0.9462 | 1.0833 | 0.7204 | ALS |
| TNF-related apoptosis-inducing ligand levels | IVW | 25 | 0.0306 | 1.0312 | 0.9712 | 1.0950 | 0.3146 | ALS |
| **Transforming growth factor-alpha levels** | **IVW** | **13** | **0.0678** | **0.8630** | **0.7556** | **0.9857** | **0.0298** | **ALS** |
| Tumor necrosis factor levels | IVW | 17 | 0.0577 | 0.9316 | 0.8320 | 1.0432 | 0.2197 | ALS |
| **Tumor necrosis factor ligand superfamily member 12 levels** | **IVW** | **26** | **0.0389** | **1.0981** | **1.0174** | **1.1851** | **0.0163** | **ALS** |
| Tumor necrosis factor ligand superfamily member 14 levels | IVW | 25 | 0.0363 | 0.9494 | 0.8842 | 1.0195 | 0.1529 | ALS |
| Tumor necrosis factor receptor superfamily member 9 levels | IVW | 25 | 0.0550 | 0.9726 | 0.8731 | 1.0833 | 0.6130 | ALS |
| Urokinase-type plasminogen activator levels | IVW | 24 | 0.0588 | 1.0254 | 0.9137 | 1.1507 | 0.6703 | ALS |
| Vascular endothelial growth factor A levels | IVW | 25 | 0.0267 | 0.9796 | 0.9297 | 1.0321 | 0.4390 | ALS |
| **Adenosine Deaminase levels** | **IVW** | **7** | **0.0871** | **0.7675** | **0.6471** | **0.9104** | **0.0024** | **MS** |
| Artemin levels | IVW | 10 | 0.1239 | 1.2172 | 0.9547 | 1.5519 | 0.1127 | MS |
| Axin-1 levels | IVW | 4 | 0.1472 | 1.2274 | 0.9197 | 1.6380 | 0.1640 | MS |
| beta-nerve growth factor levels | IVW | 16 | 0.1303 | 0.9297 | 0.7201 | 1.2003 | 0.5759 | MS |
| Caspase 8 levels | IVW | 7 | 0.1570 | 1.0477 | 0.7701 | 1.4254 | 0.7665 | MS |
| C-C motif chemokine 19 levels | IVW | 14 | 0.0794 | 1.0975 | 0.9393 | 1.2823 | 0.2416 | MS |
| C-C motif chemokine 20 levels | IVW | 12 | 0.5756 | 1.2795 | 0.4141 | 3.9538 | 0.6685 | MS |
| C-C motif chemokine 23 levels | IVW | 13 | 0.0425 | 0.9568 | 0.8803 | 1.0400 | 0.2992 | MS |
| C-C motif chemokine 25 levels | IVW | 17 | 0.0299 | 1.0155 | 0.9577 | 1.0768 | 0.6073 | MS |
| C-C motif chemokine 28 levels | IVW | 7 | 0.1520 | 1.1351 | 0.8426 | 1.5292 | 0.4045 | MS |
| C-C motif chemokine 4 levels | IVW | 8 | 0.0651 | 1.1319 | 0.9962 | 1.2860 | 0.0572 | MS |
| **CD40L receptor levels** | **IVW** | **11** | **0.0421** | **0.7737** | **0.7124** | **0.8403** | **0.0000** | **MS** |
| CUB domain-containing protein 1 levels | IVW | 19 | 0.3274 | 1.1894 | 0.6261 | 2.2595 | 0.5962 | MS |
| C-X-C motif chemokine 1 levels | IVW | 9 | 0.0497 | 0.9975 | 0.9049 | 1.0996 | 0.9596 | MS |
| **C-X-C motif chemokine 10 levels** | **IVW** | **12** | **0.0959** | **1.2726** | **1.0545** | **1.5357** | **0.0120** | **MS** |
| C-X-C motif chemokine 11 levels | IVW | 13 | 0.1065 | 1.2141 | 0.9853 | 1.4959 | 0.0686 | MS |
| C-X-C motif chemokine 5 levels | IVW | 11 | 0.0521 | 0.9699 | 0.8758 | 1.0741 | 0.5573 | MS |
| C-X-C motif chemokine 6 levels | IVW | 6 | 0.0912 | 0.9953 | 0.8324 | 1.1900 | 0.9584 | MS |
| C-X-C motif chemokine 9 levels | IVW | 12 | 0.2654 | 1.4357 | 0.8535 | 2.4153 | 0.1729 | MS |
| Cystatin D levels | IVW | 23 | 0.0270 | 0.9803 | 0.9297 | 1.0336 | 0.4609 | MS |
| Delta and Notch-like epidermal growth factor-related receptor levels | IVW | 13 | 0.1060 | 1.0527 | 0.8552 | 1.2957 | 0.6282 | MS |
| Eotaxin levels | IVW | 12 | 0.0867 | 0.9545 | 0.8053 | 1.1313 | 0.5912 | MS |
| Eukaryotic translation initiation factor 4E-binding protein 1 levels | IVW | 8 | 0.0801 | 0.9351 | 0.7993 | 1.0940 | 0.4023 | MS |
| Fibroblast growth factor 19 levels | IVW | 12 | 0.0853 | 1.1105 | 0.9396 | 1.3126 | 0.2190 | MS |
| Fibroblast growth factor 21 levels | IVW | 9 | 0.0746 | 0.9428 | 0.8146 | 1.0912 | 0.4298 | MS |
| Fibroblast growth factor 23 levels | IVW | 8 | 0.1423 | 0.8545 | 0.6466 | 1.1293 | 0.2692 | MS |
| Fibroblast growth factor 5 levels | IVW | 12 | 0.0322 | 0.9643 | 0.9054 | 1.0271 | 0.2589 | MS |
| Fms-related tyrosine kinase 3 ligand levels | IVW | 17 | 0.1519 | 1.1591 | 0.8606 | 1.5610 | 0.3311 | MS |
| Fractalkine levels | IVW | 14 | 0.7390 | 2.2283 | 0.5235 | 9.4843 | 0.2783 | MS |
| Glial cell line-derived neurotrophic factor levels | IVW | 9 | 0.0804 | 1.0434 | 0.8913 | 1.2215 | 0.5970 | MS |
| Hepatocyte growth factor levels | IVW | 14 | 0.0973 | 0.9780 | 0.8082 | 1.1834 | 0.8188 | MS |
| Interferon gamma levels | IVW | 4 | 0.1383 | 0.9698 | 0.7395 | 1.2718 | 0.8246 | MS |
| Interleukin-10 levels | IVW | 11 | 0.2378 | 0.8832 | 0.5542 | 1.4076 | 0.6016 | MS |
| Interleukin-10 receptor subunit alpha levels | IVW | 6 | 0.1750 | 1.0172 | 0.7218 | 1.4334 | 0.9226 | MS |
| Interleukin-10 receptor subunit beta levels | IVW | 7 | 0.0773 | 0.9805 | 0.8427 | 1.1408 | 0.7986 | MS |
| Interleukin-12 subunit beta levels | IVW | 19 | 0.0916 | 1.0233 | 0.8551 | 1.2244 | 0.8017 | MS |
| Interleukin-13 levels | IVW | 8 | 0.0932 | 1.0236 | 0.8526 | 1.2289 | 0.8024 | MS |
| Interleukin-15 receptor subunit alpha levels | IVW | 8 | 0.5152 | 0.8454 | 0.3079 | 2.3206 | 0.7444 | MS |
| Interleukin-17A levels | IVW | 8 | 0.3427 | 0.9946 | 0.5081 | 1.9471 | 0.9874 | MS |
| Interleukin-17C levels | IVW | 11 | 0.0997 | 1.1136 | 0.9159 | 1.3540 | 0.2805 | MS |
| Interleukin-18 levels | IVW | 13 | 0.0549 | 1.0034 | 0.9011 | 1.1173 | 0.9510 | MS |
| interleukin-18 receptor 1 levels | IVW | 11 | 0.0380 | 0.9495 | 0.8813 | 1.0230 | 0.1733 | MS |
| Interleukin-1-alpha levels | IVW | 10 | 0.3289 | 1.5177 | 0.7965 | 2.8917 | 0.2047 | MS |
| Interleukin-2 levels | IVW | 7 | 0.1091 | 1.1666 | 0.9420 | 1.4447 | 0.1579 | MS |
| Interleukin-2 receptor subunit beta levels | IVW | 8 | 0.1225 | 0.9930 | 0.7811 | 1.2625 | 0.9544 | MS |
| Interleukin-20 levels | IVW | 4 | 0.1601 | 0.7926 | 0.5791 | 1.0848 | 0.1466 | MS |
| Interleukin-20 receptor subunit alpha levels | IVW | 3 | 0.2069 | 1.1287 | 0.7524 | 1.6931 | 0.5584 | MS |
| Interleukin-22 receptor subunit alpha-1 levels | IVW | 5 | 0.1488 | 0.9554 | 0.7137 | 1.2790 | 0.7591 | MS |
| Interleukin-24 levels | IVW | 4 | 0.3346 | 1.0456 | 0.5427 | 2.0146 | 0.8939 | MS |
| Interleukin-33 levels | IVW | 7 | 0.1752 | 1.2758 | 0.9050 | 1.7984 | 0.1644 | MS |
| Interleukin-4 levels | IVW | 7 | 0.1259 | 1.2104 | 0.9457 | 1.5492 | 0.1294 | MS |
| Interleukin-5 levels | IVW | 7 | 0.1037 | 0.8372 | 0.6832 | 1.0260 | 0.0868 | MS |
| Interleukin-6 levels | IVW | 5 | 0.0879 | 0.8751 | 0.7365 | 1.0397 | 0.1291 | MS |
| Interleukin-7 levels | IVW | 6 | 0.1946 | 1.4026 | 0.9578 | 2.0540 | 0.0821 | MS |
| Interleukin-8 levels | IVW | 12 | 0.1097 | 0.8983 | 0.7246 | 1.1137 | 0.3282 | MS |
| Latency-associated peptide transforming growth factor beta 1 levels | IVW | 14 | 0.1103 | 1.0291 | 0.8291 | 1.2773 | 0.7948 | MS |
| Leukemia inhibitory factor levels | IVW | 5 | 0.2096 | 1.0024 | 0.6648 | 1.5116 | 0.9908 | MS |
| **Leukemia inhibitory factor receptor levels** | **IVW** | **13** | **0.0748** | **1.1819** | **1.0208** | **1.3685** | **0.0254** | **MS** |
| Macrophage colony-stimulating factor 1 levels | IVW | 9 | 0.1136 | 1.2179 | 0.9747 | 1.5218 | 0.0828 | MS |
| Macrophage inflammatory protein 1a levels | IVW | 9 | 0.1383 | 0.9847 | 0.7509 | 1.2913 | 0.9112 | MS |
| Matrix metalloproteinase-1 levels | IVW | 11 | 0.0730 | 0.9372 | 0.8122 | 1.0813 | 0.3740 | MS |
| Matrix metalloproteinase-10 levels | IVW | 10 | 0.0645 | 0.9043 | 0.7969 | 1.0262 | 0.1190 | MS |
| Monocyte chemoattractant protein 2 levels | IVW | 11 | 0.0310 | 1.0154 | 0.9555 | 1.0791 | 0.6219 | MS |
| Monocyte chemoattractant protein-1 levels | IVW | 11 | 0.0846 | 0.9784 | 0.8289 | 1.1549 | 0.7968 | MS |
| Monocyte chemoattractant protein-3 levels | IVW | 11 | 0.0746 | 1.0995 | 0.9500 | 1.2726 | 0.2032 | MS |
| Monocyte chemoattractant protein-4 levels | IVW | 15 | 0.0604 | 0.9317 | 0.8276 | 1.0488 | 0.2412 | MS |
| **Natural killer cell receptor 2B4 levels** | **IVW** | **14** | **0.0811** | **1.2092** | **1.0314** | **1.4176** | **0.0192** | **MS** |
| Neurotrophin-3 levels | IVW | 12 | 0.0880 | 1.0784 | 0.9076 | 1.2813 | 0.3908 | MS |
| **Neurturin levels** | **IVW** | **6** | **0.1244** | **0.7001** | **0.5486** | **0.8934** | **0.0042** | **MS** |
| Oncostatin-M levels | IVW | 10 | 0.1477 | 0.9893 | 0.7406 | 1.3216 | 0.9419 | MS |
| Osteoprotegerin levels | IVW | 10 | 0.1220 | 1.0602 | 0.8346 | 1.3467 | 0.6321 | MS |
| Programmed cell death 1 ligand 1 levels | IVW | 11 | 0.1116 | 0.9719 | 0.7809 | 1.2096 | 0.7985 | MS |
| Protein S100-A12 levels | IVW | 9 | 0.0740 | 1.0330 | 0.8935 | 1.1942 | 0.6612 | MS |
| Signaling lymphocytic activation molecule levels | IVW | 15 | 0.1354 | 1.0297 | 0.7896 | 1.3427 | 0.8290 | MS |
| SIR2-like protein 2 levels | IVW | 5 | 0.1444 | 0.9548 | 0.7195 | 1.2671 | 0.7488 | MS |
| STAM binding protein levels | IVW | 4 | 0.1895 | 1.2504 | 0.8625 | 1.8126 | 0.2383 | MS |
| Stem cell factor levels | IVW | 23 | 0.0728 | 0.9442 | 0.8186 | 1.0890 | 0.4302 | MS |
| Sulfotransferase 1A1 levels | IVW | 16 | 0.0683 | 0.9817 | 0.8588 | 1.1222 | 0.7865 | MS |
| T-cell surface glycoprotein CD5 levels | IVW | 16 | 0.1683 | 0.9609 | 0.6908 | 1.3364 | 0.8125 | MS |
| **T-cell surface glycoprotein CD6 isoform levels** | **IVW** | **11** | **0.0431** | **1.1379** | **1.0457** | **1.2384** | **0.0027** | **MS** |
| Thymic stromal lymphopoietin levels | IVW | 5 | 0.1303 | 1.0388 | 0.8047 | 1.3410 | 0.7700 | MS |
| TNF-beta levels | IVW | 12 | 0.2253 | 0.9840 | 0.6328 | 1.5303 | 0.9431 | MS |
| TNF-related activation-induced cytokine levels | IVW | 16 | 0.0679 | 0.9679 | 0.8474 | 1.1056 | 0.6305 | MS |
| TNF-related apoptosis-inducing ligand levels | IVW | 15 | 0.0455 | 1.0607 | 0.9703 | 1.1596 | 0.1948 | MS |
| Transforming growth factor-alpha levels | IVW | 10 | 0.2696 | 0.8186 | 0.4826 | 1.3885 | 0.4578 | MS |
| Tumor necrosis factor levels | IVW | 12 | 0.0831 | 1.1497 | 0.9768 | 1.3531 | 0.0934 | MS |
| Tumor necrosis factor ligand superfamily member 12 levels | IVW | 21 | 0.0766 | 0.9952 | 0.8564 | 1.1565 | 0.9501 | MS |
| Tumor necrosis factor ligand superfamily member 14 levels | IVW | 14 | 0.0831 | 1.0699 | 0.9091 | 1.2592 | 0.4161 | MS |
| Tumor necrosis factor receptor superfamily member 9 levels | IVW | 17 | 0.1570 | 0.9675 | 0.7113 | 1.3160 | 0.8334 | MS |
| Urokinase-type plasminogen activator levels | IVW | 17 | 0.0932 | 1.0626 | 0.8851 | 1.2756 | 0.5150 | MS |
| Vascular endothelial growth factor A levels | IVW | 13 | 0.0790 | 0.9460 | 0.8104 | 1.1044 | 0.4824 | MS |
| Adenosine Deaminase levels | IVW | 14 | 0.0416 | 0.9262 | 0.8537 | 1.0049 | 0.0656 | PD |
| Artemin levels | IVW | 17 | 0.1102 | 0.8693 | 0.7005 | 1.0788 | 0.2035 | PD |
| Axin-1 levels | IVW | 7 | 0.1237 | 0.8975 | 0.7043 | 1.1438 | 0.3822 | PD |
| beta-nerve growth factor levels | IVW | 20 | 0.1062 | 1.0171 | 0.8260 | 1.2524 | 0.8731 | PD |
| Caspase 8 levels | IVW | 12 | 0.0854 | 0.9075 | 0.7676 | 1.0729 | 0.2559 | PD |
| C-C motif chemokine 19 levels | IVW | 19 | 0.0646 | 0.9954 | 0.8770 | 1.1298 | 0.9432 | PD |
| C-C motif chemokine 20 levels | IVW | 16 | 0.0794 | 0.9848 | 0.8429 | 1.1507 | 0.8474 | PD |
| C-C motif chemokine 23 levels | IVW | 23 | 0.0763 | 0.9769 | 0.8411 | 1.1345 | 0.7593 | PD |
| C-C motif chemokine 25 levels | IVW | 25 | 0.0278 | 1.0240 | 0.9697 | 1.0814 | 0.3934 | PD |
| C-C motif chemokine 28 levels | IVW | 15 | 0.0909 | 1.0311 | 0.8628 | 1.2322 | 0.7362 | PD |
| C-C motif chemokine 4 levels | IVW | 17 | 0.0409 | 1.0524 | 0.9713 | 1.1402 | 0.2119 | PD |
| CD40L receptor levels | IVW | 13 | 0.0443 | 0.9866 | 0.9046 | 1.0760 | 0.7602 | PD |
| CUB domain-containing protein 1 levels | IVW | 22 | 0.0713 | 0.9746 | 0.8475 | 1.1209 | 0.7187 | PD |
| C-X-C motif chemokine 1 levels | IVW | 9 | 0.0748 | 0.9302 | 0.8033 | 1.0771 | 0.3334 | PD |
| C-X-C motif chemokine 10 levels | IVW | 17 | 0.0634 | 0.9755 | 0.8615 | 1.1046 | 0.6956 | PD |
| C-X-C motif chemokine 11 levels | IVW | 18 | 0.0699 | 1.0472 | 0.9130 | 1.2011 | 0.5096 | PD |
| C-X-C motif chemokine 5 levels | IVW | 12 | 0.0539 | 1.0054 | 0.9046 | 1.1175 | 0.9204 | PD |
| C-X-C motif chemokine 6 levels | IVW | 10 | 0.0481 | 0.9647 | 0.8779 | 1.0600 | 0.4548 | PD |
| C-X-C motif chemokine 9 levels | IVW | 18 | 0.0845 | 0.9515 | 0.8063 | 1.1228 | 0.5562 | PD |
| Cystatin D levels | IVW | 31 | 0.0285 | 0.9471 | 0.8956 | 1.0016 | 0.0570 | PD |
| Delta and Notch-like epidermal growth factor-related receptor levels | IVW | 14 | 0.0782 | 1.0078 | 0.8646 | 1.1747 | 0.9207 | PD |
| Eotaxin levels | IVW | 17 | 0.0632 | 0.9597 | 0.8480 | 1.0862 | 0.5152 | PD |
| Eukaryotic translation initiation factor 4E-binding protein 1 levels | IVW | 10 | 0.0889 | 1.0629 | 0.8929 | 1.2653 | 0.4925 | PD |
| Fibroblast growth factor 19 levels | IVW | 19 | 0.0624 | 1.0220 | 0.9043 | 1.1549 | 0.7277 | PD |
| **Fibroblast growth factor 21 levels** | **IVW** | **13** | **0.0978** | **0.8169** | **0.6744** | **0.9896** | **0.0387** | **PD** |
| Fibroblast growth factor 23 levels | IVW | 9 | 0.1120 | 1.1927 | 0.9577 | 1.4854 | 0.1155 | PD |
| Fibroblast growth factor 5 levels | IVW | 22 | 0.0345 | 1.0376 | 0.9698 | 1.1102 | 0.2842 | PD |
| Fms-related tyrosine kinase 3 ligand levels | IVW | 25 | 0.0526 | 0.9384 | 0.8465 | 1.0402 | 0.2263 | PD |
| Fractalkine levels | IVW | 18 | 0.0946 | 1.1158 | 0.9270 | 1.3431 | 0.2467 | PD |
| Glial cell line-derived neurotrophic factor levels | IVW | 13 | 0.0845 | 0.9494 | 0.8045 | 1.1205 | 0.5393 | PD |
| Hepatocyte growth factor levels | IVW | 17 | 0.0823 | 1.0302 | 0.8767 | 1.2106 | 0.7177 | PD |
| Interferon gamma levels | IVW | 8 | 0.1263 | 0.9779 | 0.7636 | 1.2525 | 0.8598 | PD |
| Interleukin-10 levels | IVW | 17 | 0.0885 | 1.0682 | 0.8981 | 1.2704 | 0.4560 | PD |
| Interleukin-10 receptor subunit alpha levels | IVW | 10 | 0.1358 | 1.0042 | 0.7696 | 1.3103 | 0.9756 | PD |
| Interleukin-10 receptor subunit beta levels | IVW | 16 | 0.0752 | 0.9877 | 0.8523 | 1.1446 | 0.8693 | PD |
| **Interleukin-12 subunit beta levels** | **IVW** | **23** | **0.0502** | **0.8987** | **0.8145** | **0.9916** | **0.0333** | **PD** |
| Interleukin-13 levels | IVW | 10 | 0.1213 | 1.0310 | 0.8128 | 1.3077 | 0.8013 | PD |
| Interleukin-15 receptor subunit alpha levels | IVW | 10 | 0.0514 | 0.9679 | 0.8751 | 1.0705 | 0.5252 | PD |
| **Interleukin-17A levels** | **IVW** | **12** | **0.1026** | **1.2852** | **1.0511** | **1.5714** | **0.0145** | **PD** |
| Interleukin-17C levels | IVW | 15 | 0.0775 | 0.9865 | 0.8475 | 1.1484 | 0.8611 | PD |
| Interleukin-18 levels | IVW | 19 | 0.0628 | 1.1224 | 0.9924 | 1.2696 | 0.0661 | PD |
| interleukin-18 receptor 1 levels | IVW | 22 | 0.0314 | 0.9986 | 0.9391 | 1.0620 | 0.9655 | PD |
| Interleukin-1-alpha levels | IVW | 12 | 0.0782 | 0.9562 | 0.8203 | 1.1145 | 0.5664 | PD |
| Interleukin-2 levels | IVW | 15 | 0.0935 | 0.9513 | 0.7919 | 1.1427 | 0.5932 | PD |
| Interleukin-2 receptor subunit beta levels | IVW | 11 | 0.1109 | 1.0077 | 0.8108 | 1.2524 | 0.9448 | PD |
| Interleukin-20 levels | IVW | 10 | 0.1161 | 1.1183 | 0.8906 | 1.4042 | 0.3358 | PD |
| Interleukin-20 receptor subunit alpha levels | IVW | 6 | 0.1475 | 0.8332 | 0.6240 | 1.1125 | 0.2160 | PD |
| Interleukin-22 receptor subunit alpha-1 levels | IVW | 9 | 0.1172 | 1.0231 | 0.8131 | 1.2874 | 0.8453 | PD |
| Interleukin-24 levels | IVW | 8 | 0.1600 | 0.9775 | 0.7144 | 1.3376 | 0.8871 | PD |
| Interleukin-33 levels | IVW | 9 | 0.1250 | 0.9109 | 0.7130 | 1.1638 | 0.4555 | PD |
| Interleukin-4 levels | IVW | 9 | 0.1122 | 0.9242 | 0.7417 | 1.1516 | 0.4823 | PD |
| Interleukin-5 levels | IVW | 9 | 0.1145 | 0.9815 | 0.7842 | 1.2283 | 0.8702 | PD |
| Interleukin-6 levels | IVW | 7 | 0.1085 | 1.0112 | 0.8175 | 1.2508 | 0.9183 | PD |
| Interleukin-7 levels | IVW | 8 | 0.1199 | 1.0045 | 0.7941 | 1.2707 | 0.9700 | PD |
| Interleukin-8 levels | IVW | 15 | 0.0925 | 1.0453 | 0.8720 | 1.2530 | 0.6321 | PD |
| Latency-associated peptide transforming growth factor beta 1 levels | IVW | 16 | 0.0802 | 0.9564 | 0.8172 | 1.1192 | 0.5782 | PD |
| Leukemia inhibitory factor levels | IVW | 10 | 0.1235 | 1.1659 | 0.9153 | 1.4851 | 0.2139 | PD |
| Leukemia inhibitory factor receptor levels | IVW | 15 | 0.1045 | 1.1191 | 0.9118 | 1.3735 | 0.2816 | PD |
| Macrophage colony-stimulating factor 1 levels | IVW | 14 | 0.0990 | 1.1262 | 0.9275 | 1.3674 | 0.2300 | PD |
| Macrophage inflammatory protein 1a levels | IVW | 11 | 0.0540 | 0.9920 | 0.8923 | 1.1029 | 0.8825 | PD |
| Matrix metalloproteinase-1 levels | IVW | 15 | 0.0633 | 0.9827 | 0.8680 | 1.1125 | 0.7824 | PD |
| Matrix metalloproteinase-10 levels | IVW | 14 | 0.0418 | 1.0120 | 0.9323 | 1.0984 | 0.7762 | PD |
| Monocyte chemoattractant protein 2 levels | IVW | 22 | 0.0296 | 1.0084 | 0.9515 | 1.0688 | 0.7767 | PD |
| Monocyte chemoattractant protein-1 levels | IVW | 22 | 0.0563 | 0.9963 | 0.8923 | 1.1124 | 0.9474 | PD |
| Monocyte chemoattractant protein-3 levels | IVW | 15 | 0.0623 | 0.9591 | 0.8489 | 1.0836 | 0.5022 | PD |
| Monocyte chemoattractant protein-4 levels | IVW | 16 | 0.0550 | 0.9848 | 0.8843 | 1.0968 | 0.7808 | PD |
| Natural killer cell receptor 2B4 levels | IVW | 20 | 0.0580 | 0.9956 | 0.8886 | 1.1155 | 0.9394 | PD |
| Neurotrophin-3 levels | IVW | 15 | 0.1140 | 1.0555 | 0.8442 | 1.3199 | 0.6354 | PD |
| **Neurturin levels** | **IVW** | **11** | **0.1097** | **0.7346** | **0.5925** | **0.9109** | **0.0049** | **PD** |
| Oncostatin-M levels | IVW | 13 | 0.0796 | 0.8892 | 0.7607 | 1.0393 | 0.1401 | PD |
| Osteoprotegerin levels | IVW | 17 | 0.0661 | 0.9695 | 0.8516 | 1.1036 | 0.6393 | PD |
| Programmed cell death 1 ligand 1 levels | IVW | 15 | 0.0889 | 1.0581 | 0.8889 | 1.2596 | 0.5250 | PD |
| Protein S100-A12 levels | IVW | 12 | 0.0879 | 1.1061 | 0.9310 | 1.3141 | 0.2514 | PD |
| Signaling lymphocytic activation molecule levels | IVW | 20 | 0.0717 | 0.9481 | 0.8239 | 1.0910 | 0.4570 | PD |
| SIR2-like protein 2 levels | IVW | 11 | 0.1026 | 1.0964 | 0.8967 | 1.3407 | 0.3696 | PD |
| STAM binding protein levels | IVW | 9 | 0.1231 | 0.9838 | 0.7730 | 1.2522 | 0.8946 | PD |
| Stem cell factor levels | IVW | 28 | 0.0446 | 1.0868 | 0.9958 | 1.1861 | 0.0620 | PD |
| Sulfotransferase 1A1 levels | IVW | 18 | 0.0814 | 1.1431 | 0.9745 | 1.3409 | 0.1005 | PD |
| T-cell surface glycoprotein CD5 levels | IVW | 19 | 0.0846 | 1.0045 | 0.8511 | 1.1857 | 0.9575 | PD |
| T-cell surface glycoprotein CD6 isoform levels | IVW | 10 | 0.0390 | 1.0350 | 0.9588 | 1.1172 | 0.3786 | PD |
| Thymic stromal lymphopoietin levels | IVW | 13 | 0.1063 | 1.1733 | 0.9527 | 1.4450 | 0.1326 | PD |
| TNF-beta levels | IVW | 19 | 0.0280 | 1.0390 | 0.9836 | 1.0976 | 0.1711 | PD |
| TNF-related activation-induced cytokine levels | IVW | 25 | 0.0503 | 1.0349 | 0.9377 | 1.1421 | 0.4954 | PD |
| TNF-related apoptosis-inducing ligand levels | IVW | 24 | 0.0521 | 0.9714 | 0.8772 | 1.0759 | 0.5780 | PD |
| **Transforming growth factor-alpha levels** | **IVW** | **12** | **0.0969** | **0.8254** | **0.6826** | **0.9982** | **0.0478** | **PD** |
| Tumor necrosis factor levels | IVW | 15 | 0.0841 | 0.9448 | 0.8012 | 1.1142 | 0.5000 | PD |
| Tumor necrosis factor ligand superfamily member 12 levels | IVW | 22 | 0.0698 | 0.9486 | 0.8273 | 1.0877 | 0.4496 | PD |
| Tumor necrosis factor ligand superfamily member 14 levels | IVW | 24 | 0.0503 | 1.0491 | 0.9505 | 1.1579 | 0.3414 | PD |
| **Tumor necrosis factor receptor superfamily member 9 levels** | **IVW** | **23** | **0.0658** | **0.8451** | **0.7429** | **0.9613** | **0.0105** | **PD** |
| Urokinase-type plasminogen activator levels | IVW | 20 | 0.0652 | 1.0085 | 0.8876 | 1.1460 | 0.8961 | PD |
| Vascular endothelial growth factor A levels | IVW | 20 | 0.0892 | 0.9632 | 0.8087 | 1.1473 | 0.6747 | PD |

**Table S2.** Different MR analysis of circulating inflammatory cytokines on AD, ALS, MS, and PD.

| **Exposure** | **Outcome** | **Method** | **nSNP** | **Beta** | **SE** | **OR** | **OR_lci95** | **OR_uci95** | **P-value** |
| --- | --- | --- | --- | --- | --- | --- | --- | --- | --- |
| C-C motif chemokine 19 levels | AD | IVW | 21 | 0.0005 | 0.0003 | 1.0005 | 1.0000 | 1.0010 | 0.0478 |
|  |  | MR Egger | 21 | 0.0005 | 0.0004 | 1.0010 | 1.0001 | 1.0019 | 0.9744 |
|  |  | Simple median | 21 | 0.0007 | 0.0004 | 1.0007 | 1.0000 | 1.0015 | 0.0551 |
|  |  | Weighted median | 21 | 0.0007 | 0.0004 | 1.0007 | 1.0000 | 1.0014 | 0.0673 |
|  |  | Weighted mode | 21 | 0.0006 | 0.0004 | 1.0006 | 0.9998 | 1.0014 | 0.1523 |
| C-X-C motif chemokine 1 levels | AD | IVW | 11 | -0.0007 | 0.0003 | 0.9993 | 0.9986 | 1.0000 | 0.0424 |
|  |  | MR Egger | 11 | -0.0007 | 0.0006 | 0.9993 | 0.9982 | 1.0005 | 0.2799 |
|  |  | Simple median | 11 | -0.0006 | 0.0006 | 0.9994 | 0.9982 | 1.0006 | 0.3214 |
|  |  | Weighted median | 11 | -0.0006 | 0.0003 | 0.9994 | 0.9987 | 1.0001 | 0.0881 |
|  |  | Weighted mode | 11 | -0.0006 | 0.0004 | 0.9994 | 0.9987 | 1.0002 | 0.1626 |
| Fms-related tyrosine kinase 3 ligand levels | AD | IVW | 26 | 0.0005 | 0.0002 | 1.0005 | 1.0001 | 1.0010 | 0.0210 |
|  |  | MR Egger | 26 | 0.0003 | 0.0004 | 1.0003 | 0.9996 | 1.0010 | 0.3726 |
|  |  | Simple median | 26 | 0.0006 | 0.0004 | 1.0006 | 0.9998 | 1.0013 | 0.1309 |
|  |  | Weighted median | 26 | 0.0003 | 0.0003 | 1.0003 | 0.9996 | 1.0009 | 0.4204 |
|  |  | Weighted mode | 26 | 0.0004 | 0.0004 | 1.0004 | 0.9997 | 1.0011 | 0.2706 |
| Interleukin-18 levels | AD | IVW | 19 | -0.0006 | 0.0003 | 0.9994 | 0.9989 | 0.9999 | 0.0186 |
|  |  | MR Egger | 19 | -0.0004 | 0.0005 | 0.9996 | 0.9985 | 1.0006 | 0.4217 |
|  |  | Simple median | 19 | -0.0011 | 0.0004 | 0.9989 | 0.9981 | 0.9997 | 0.0099 |
|  |  | Weighted median | 19 | -0.0004 | 0.0004 | 0.9996 | 0.9989 | 1.0003 | 0.2172 |
|  |  | Weighted mode | 19 | -0.0004 | 0.0004 | 0.9996 | 0.9989 | 1.0003 | 0.2357 |
| Leukemia inhibitory factor receptor levels | AD | IVW | 18 | -0.0007 | 0.0003 | 0.9993 | 0.9988 | 0.9999 | 0.0122 |
|  |  | MR Egger | 18 | -0.0014 | 0.0005 | 0.9986 | 0.9976 | 0.9996 | 0.0125 |
|  |  | Simple median | 18 | -0.0004 | 0.0004 | 0.9996 | 0.9987 | 1.0004 | 0.3419 |
|  |  | Weighted median | 18 | -0.0010 | 0.0004 | 0.9990 | 0.9983 | 0.9998 | 0.0088 |
|  |  | Weighted mode | 18 | -0.0013 | 0.0004 | 0.9987 | 0.9979 | 0.9995 | 0.0069 |
| Monocyte chemoattractant protein-1 levels | AD | IVW | 22 | -0.0008 | 0.0003 | 0.9992 | 0.9987 | 0.9997 | 0.0026 |
|  |  | MR Egger | 22 | -0.0007 | 0.0005 | 0.9993 | 0.9983 | 1.0003 | 0.1674 |
|  |  | Simple median | 22 | -0.0007 | 0.0004 | 0.9993 | 0.9985 | 1.0001 | 0.0930 |
|  |  | Weighted median | 22 | -0.0006 | 0.0004 | 0.9994 | 0.9986 | 1.0002 | 0.1504 |
|  |  | Weighted mode | 22 | -0.0013 | 0.0006 | 0.9987 | 0.9976 | 0.9998 | 0.0307 |
| SIR2-like protein 2 levels | AD | IVW | 12 | 0.0010 | 0.0004 | 1.0010 | 1.0002 | 1.0018 | 0.0155 |
|  |  | MR Egger | 12 | 0.0017 | 0.0008 | 1.0017 | 1.0002 | 1.0032 | 0.0560 |
|  |  | Simple median | 12 | 0.0011 | 0.0006 | 1.0011 | 1.0000 | 1.0022 | 0.0527 |
|  |  | Weighted median | 12 | 0.0013 | 0.0006 | 1.0013 | 1.0002 | 1.0024 | 0.0188 |
|  |  | Weighted mode | 12 | 0.0014 | 0.0007 | 1.0014 | 1.0001 | 1.0028 | 0.0605 |
| C-X-C motif chemokine 10 levels | ALS | IVW | 22 | -0.1208 | 0.0448 | 0.8862 | 0.8116 | 0.9676 | 0.0071 |
|  |  | MR Egger | 22 | -0.0299 | 0.0912 | 0.9705 | 0.8117 | 1.1603 | 0.7459 |
|  |  | Simple median | 22 | -0.1323 | 0.0694 | 0.8761 | 0.7646 | 1.0038 | 0.0568 |
|  |  | Weighted median | 22 | -0.0767 | 0.0671 | 0.9262 | 0.8120 | 1.0563 | 0.2530 |
|  |  | Weighted mode | 22 | -0.0799 | 0.1026 | 0.9304 | 0.8426 | 1.2600 | 0.7734 |
| C-X-C motif chemokine 11 levels | ALS | IVW | 23 | -0.1016 | 0.0473 | 0.9034 | 0.8234 | 0.9911 | 0.0316 |
|  |  | MR Egger | 23 | 0.0075 | 0.1198 | 1.0076 | 0.7967 | 1.2743 | 0.9504 |
|  |  | Simple median | 23 | -0.0690 | 0.0690 | 0.9333 | 0.8152 | 1.0685 | 0.3172 |
|  |  | Weighted median | 23 | -0.0786 | 0.0665 | 0.9244 | 0.8114 | 1.0531 | 0.2373 |
|  |  | Weighted mode | 23 | -0.0940 | 0.0841 | 0.9103 | 0.7720 | 1.0734 | 0.2758 |
| Hepatocyte growth factor levels | ALS | IVW | 16 | 0.1215 | 0.0561 | 1.1291 | 1.0116 | 1.2604 | 0.0304 |
|  |  | MR Egger | 16 | 0.1821 | 0.1414 | 1.1997 | 0.9093 | 1.5829 | 0.2189 |
|  |  | Simple median | 16 | 0.1532 | 0.0753 | 1.1656 | 1.0057 | 1.3510 | 0.0418 |
|  |  | Weighted median | 16 | 0.0770 | 0.0759 | 1.0800 | 0.9307 | 1.2533 | 0.3105 |
|  |  | Weighted mode | 16 | 0.0585 | 0.1030 | 1.0602 | 0.8664 | 1.2974 | 0.5786 |
| Interleukin-2 receptor subunit beta levels | ALS | IVW | 13 | -0.1292 | 0.0655 | 0.8788 | 0.7728 | 0.9992 | 0.0486 |
|  |  | MR Egger | 13 | -0.1366 | 0.1329 | 0.8723 | 0.6723 | 1.1319 | 0.3262 |
|  |  | Simple median | 13 | -0.1210 | 0.0946 | 0.8860 | 0.7360 | 1.0666 | 0.2009 |
|  |  | Weighted median | 13 | -0.1254 | 0.0927 | 0.8821 | 0.7356 | 1.0579 | 0.1762 |
|  |  | Weighted mode | 13 | -0.1549 | 0.1489 | 0.8565 | 0.6397 | 1.1468 | 0.3187 |
| T-cell surface glycoprotein CD5 levels | ALS | IVW | 22 | -0.1182 | 0.0514 | 0.8885 | 0.8033 | 0.9828 | 0.0216 |
|  |  | MR Egger | 22 | -0.3641 | 0.1377 | 0.6948 | 0.5305 | 0.9100 | 0.0155 |
|  |  | Simple median | 22 | -0.1355 | 0.0728 | 0.8733 | 0.7571 | 1.0073 | 0.0629 |
|  |  | Weighted median | 22 | -0.1631 | 0.0653 | 0.8495 | 0.7474 | 0.9656 | 0.0126 |
|  |  | Weighted mode | 22 | -0.2348 | 0.0762 | 0.7907 | 0.6810 | 0.9181 | 0.0057 |
| Transforming growth factor-alpha levels | ALS | IVW | 13 | -0.1474 | 0.0678 | 0.8630 | 0.7556 | 0.9857 | 0.0298 |
|  |  | MR Egger | 13 | -0.1632 | 0.1987 | 0.8494 | 0.5754 | 1.2540 | 0.4291 |
|  |  | Simple median | 13 | -0.1241 | 0.0930 | 0.8833 | 0.7362 | 1.0598 | 0.1819 |
|  |  | Weighted median | 13 | -0.1241 | 0.0869 | 0.8833 | 0.7449 | 1.0473 | 0.1533 |
|  |  | Weighted mode | 13 | -0.1114 | 0.1259 | 0.8946 | 0.6989 | 1.1450 | 0.3936 |
| Tumor necrosis factor ligand superfamily member 12 levels | ALS | IVW | 26 | 0.0935 | 0.0389 | 1.0981 | 1.0174 | 1.1851 | 0.0163 |
|  |  | MR Egger | 26 | 0.2175 | 0.0862 | 1.2429 | 1.0497 | 1.4716 | 0.0187 |
|  |  | Simple median | 26 | 0.0846 | 0.0603 | 1.0883 | 0.9671 | 1.2248 | 0.1603 |
|  |  | Weighted median | 26 | 0.1349 | 0.0576 | 1.1444 | 1.0222 | 1.2811 | 0.0192 |
|  |  | Weighted mode | 26 | 0.1508 | 0.0621 | 1.1627 | 1.0294 | 1.3133 | 0.0228 |
| Adenosine Deaminase levels | MS | IVW | 7 | -0.2646 | 0.0871 | 0.7675 | 0.6471 | 0.9104 | 0.0024 |
|  |  | MR Egger | 7 | -0.1714 | 0.1939 | 0.8425 | 0.5761 | 1.2319 | 0.4171 |
|  |  | Simple median | 7 | -0.2947 | 0.1365 | 0.7447 | 0.5699 | 0.9731 | 0.0308 |
|  |  | Weighted median | 7 | -0.2036 | 0.1060 | 0.8158 | 0.6628 | 1.0042 | 0.0547 |
|  |  | Weighted mode | 7 | -0.1972 | 0.1182 | 0.8210 | 0.6512 | 1.0351 | 0.1463 |
| CD40L receptor levels | MS | IVW | 11 | -0.2566 | 0.0421 | 0.7737 | 0.7124 | 0.8403 | 0.0000 |
|  |  | MR Egger | 11 | -0.3127 | 0.0518 | 0.7314 | 0.6608 | 0.8096 | 0.0002 |
|  |  | Simple median | 11 | -0.1762 | 0.1214 | 0.8384 | 0.6609 | 1.0636 | 0.1465 |
|  |  | Weighted median | 11 | -0.2857 | 0.0406 | 0.7515 | 0.6940 | 0.8137 | 0.0000 |
|  |  | Weighted mode | 11 | -0.2871 | 0.0418 | 0.7504 | 0.6914 | 0.8145 | 0.0000 |
| C-X-C motif chemokine 10 levels | MS | IVW | 12 | 0.2410 | 0.0959 | 1.2726 | 1.0545 | 1.5357 | 0.0120 |
|  |  | MR Egger | 12 | 0.3166 | 0.2412 | 1.3724 | 0.8554 | 2.2019 | 0.2187 |
|  |  | Simple median | 12 | 0.0726 | 0.1274 | 1.0753 | 0.8377 | 1.3804 | 0.5687 |
|  |  | Weighted median | 12 | 0.1982 | 0.1211 | 1.2192 | 0.9617 | 1.5457 | 0.1016 |
|  |  | Weighted mode | 12 | 0.1125 | 0.2219 | 1.1191 | 0.7244 | 1.7288 | 0.6222 |
| Leukemia inhibitory factor receptor levels | MS | IVW | 13 | 0.1671 | 0.0748 | 1.1819 | 1.0208 | 1.3685 | 0.0254 |
|  |  | MR Egger | 13 | 0.2918 | 0.1253 | 1.3389 | 1.0473 | 1.7117 | 0.0400 |
|  |  | Simple median | 13 | -0.0318 | 0.1254 | 0.9687 | 0.7577 | 1.2386 | 0.8000 |
|  |  | Weighted median | 13 | 0.2022 | 0.0661 | 1.2240 | 1.0753 | 1.3934 | 0.0022 |
|  |  | Weighted mode | 13 | 0.1946 | 0.0677 | 1.2149 | 1.0640 | 1.3871 | 0.0139 |
| Natural killer cell receptor 2B4 levels | MS | IVW | 14 | 0.1899 | 0.0811 | 1.2092 | 1.0314 | 1.4176 | 0.0192 |
|  |  | MR Egger | 14 | 0.0995 | 0.1877 | 1.1046 | 0.7647 | 1.5957 | 0.6057 |
|  |  | Simple median | 14 | 0.1597 | 0.1048 | 1.1732 | 0.9553 | 1.4408 | 0.1276 |
|  |  | Weighted median | 14 | 0.0394 | 0.0773 | 1.0402 | 0.8939 | 1.2104 | 0.6106 |
|  |  | Weighted mode | 14 | 0.0402 | 0.0766 | 1.0410 | 0.8959 | 1.2097 | 0.6085 |
| Neurturin levels | MS | IVW | 6 | -0.3566 | 0.1244 | 0.7001 | 0.5486 | 0.8934 | 0.0042 |
|  |  | MR Egger | 6 | -0.5376 | 0.4576 | 0.5841 | 0.2382 | 1.4324 | 0.3052 |
|  |  | Simple median | 6 | -0.3698 | 0.1518 | 0.6909 | 0.5131 | 0.9303 | 0.0148 |
|  |  | Weighted median | 6 | -0.3490 | 0.1511 | 0.7054 | 0.5246 | 0.9486 | 0.0209 |
|  |  | Weighted mode | 6 | -0.3557 | 0.2030 | 0.7007 | 0.4707 | 1.0431 | 0.1401 |
| T-cell surface glycoprotein CD6 isoform levels | MS | IVW | 11 | 0.1292 | 0.0431 | 1.1379 | 1.0457 | 1.2384 | 0.0027 |
|  |  | MR Egger | 11 | 0.1021 | 0.0624 | 1.1074 | 0.9799 | 1.2515 | 0.1364 |
|  |  | Simple median | 11 | 0.0189 | 0.1092 | 1.0191 | 0.8227 | 1.2623 | 0.8626 |
|  |  | Weighted median | 11 | 0.1126 | 0.0315 | 1.1192 | 1.0522 | 1.1904 | 0.0004 |
|  |  | Weighted mode | 11 | 0.1096 | 0.0338 | 1.1158 | 1.0444 | 1.1922 | 0.0088 |
| Fibroblast growth factor 21 levels | PD | IVW | 13 | -0.2022 | 0.0978 | 0.8169 | 0.6744 | 0.9896 | 0.0387 |
|  |  | MR Egger | 13 | -0.2210 | 0.2475 | 0.8017 | 0.4935 | 1.3024 | 0.3912 |
|  |  | Simple median | 13 | 0.0863 | 0.1367 | 1.0901 | 0.8338 | 1.4251 | 0.5281 |
|  |  | Weighted median | 13 | -0.1273 | 0.1254 | 0.8804 | 0.6885 | 1.1258 | 0.3101 |
|  |  | Weighted mode | 13 | -0.4846 | 0.1625 | 0.6159 | 0.4480 | 0.8469 | 0.0114 |
| Interleukin-12 subunit beta levels | PD | IVW | 23 | -0.1068 | 0.0502 | 0.8987 | 0.8145 | 0.9916 | 0.0333 |
|  |  | MR Egger | 23 | -0.0100 | 0.1098 | 0.9901 | 0.7983 | 1.2279 | 0.9283 |
|  |  | Simple median | 23 | -0.1212 | 0.0813 | 0.8859 | 0.7554 | 1.0388 | 0.1359 |
|  |  | Weighted median | 23 | -0.0305 | 0.0774 | 0.9699 | 0.8333 | 1.1289 | 0.6936 |
|  |  | Weighted mode | 23 | -0.0130 | 0.0787 | 0.9870 | 0.8460 | 1.1516 | 0.8698 |
| Interleukin-17A levels | PD | IVW | 12 | 0.2509 | 0.1026 | 1.2852 | 1.0511 | 1.5714 | 0.0145 |
|  |  | MR Egger | 12 | 0.2192 | 0.2440 | 1.2450 | 0.7717 | 2.0086 | 0.3902 |
|  |  | Simple median | 12 | 0.2082 | 0.1315 | 1.2314 | 0.9516 | 1.5936 | 0.1135 |
|  |  | Weighted median | 12 | 0.2080 | 0.1353 | 1.2312 | 0.9444 | 1.6051 | 0.1242 |
|  |  | Weighted mode | 12 | 0.2147 | 0.2165 | 1.2394 | 0.8108 | 1.8947 | 0.3428 |
| Neurturin levels | PD | IVW | 11 | -0.3084 | 0.1097 | 0.7346 | 0.5925 | 0.9109 | 0.0049 |
|  |  | MR Egger | 11 | -0.4031 | 0.2063 | 0.6682 | 0.4460 | 1.0011 | 0.0824 |
|  |  | Simple median | 11 | -0.3300 | 0.1564 | 0.7189 | 0.5291 | 0.9769 | 0.0349 |
|  |  | Weighted median | 11 | -0.2969 | 0.1414 | 0.7431 | 0.5632 | 0.9805 | 0.0358 |
|  |  | Weighted mode | 11 | -0.3144 | 0.2124 | 0.7303 | 0.4816 | 1.1072 | 0.1696 |
| Transforming growth factor-alpha levels | PD | IVW | 12 | -0.1918 | 0.0969 | 0.8254 | 0.6826 | 0.9982 | 0.0478 |
|  |  | MR Egger | 12 | -0.0570 | 0.2493 | 0.9446 | 0.5794 | 1.5398 | 0.8237 |
|  |  | Simple median | 12 | -0.1713 | 0.1450 | 0.8426 | 0.6342 | 1.1195 | 0.2373 |
|  |  | Weighted median | 12 | -0.0929 | 0.1402 | 0.9113 | 0.6924 | 1.1993 | 0.5073 |
|  |  | Weighted mode | 12 | -0.0435 | 0.1943 | 0.9575 | 0.6542 | 1.4013 | 0.8270 |
| Tumor necrosis factor receptor superfamily member 9 levels | PD | IVW | 23 | -0.1683 | 0.0658 | 0.8451 | 0.7429 | 0.9613 | 0.0105 |
|  |  | MR Egger | 23 | -0.2115 | 0.1592 | 0.8094 | 0.5924 | 1.1059 | 0.1984 |
|  |  | Simple median | 23 | -0.2128 | 0.0900 | 0.8083 | 0.6776 | 0.9642 | 0.0180 |
|  |  | Weighted median | 23 | -0.1795 | 0.0959 | 0.8357 | 0.6925 | 1.0086 | 0.0614 |
|  |  | Weighted mode | 23 | -0.1660 | 0.1190 | 0.8470 | 0.6708 | 1.0695 | 0.1768 |

**Table S3.** Characteristics of the genetic instrument variables for selected cytokines at the genome-wide significance level for AD, ALS, MS, and PD (P < 5 × 10^–6^).

| **Exposure** | **Outcome** | **SNP ID** | **chr** | **pos** | **Effect allele** | **Other allele** | **BETA** | **SE** | **EAF** | ***P*-value** | ***F*** |
| --- | --- | --- | --- | --- | --- | --- | --- | --- | --- | --- | --- |
| C-C motif chemokine 19 levels | AD | rs7595241 | 2 | 7425475 | C | G | 0.0615 | 0.013 | 0.6941 | 2.24E-06 | 22.38018 |
|  |  | rs10496135 | 2 | 66640363 | C | G | 0.1856 | 0.0384 | 0.9689 | 1.34E-06 | 23.36111 |
|  |  | rs13010492 | 2 | 204776176 | C | G | -0.0803 | 0.0123 | 0.4754 | 6.65E-11 | 42.62073 |
|  |  | rs113539352 | 3 | 58516854 | C | G | 0.4485 | 0.0922 | 0.992 | 1.15E-06 | 23.66263 |
|  |  | rs62292952 | 3 | 132200719 | T | G | 0.2346 | 0.0185 | 0.8822 | 7.53E-37 | 160.8098 |
|  |  | rs79086127 | 4 | 123839263 | T | C | -0.2096 | 0.045 | 0.0195 | 3.20E-06 | 21.69489 |
|  |  | rs4554017 | 4 | 145124024 | T | C | 0.0596 | 0.0122 | 0.3904 | 1.03E-06 | 23.86563 |
|  |  | rs3792790 | 5 | 150442171 | A | C | 0.0551 | 0.012 | 0.4736 | 4.40E-06 | 21.0834 |
|  |  | rs6870560 | 5 | 174187919 | T | C | 0.0863 | 0.0169 | 0.8559 | 3.28E-07 | 26.07643 |
|  |  | rs9469127 | 6 | 32444093 | C | G | -0.4884 | 0.0277 | 0.9365 | 1.41E-69 | 310.8793 |
|  |  | rs28635588 | 6 | 32519041 | A | G | -0.1775 | 0.0206 | 0.8408 | 6.90E-18 | 74.24416 |
|  |  | rs1415763 | 6 | 125027776 | C | G | 0.0757 | 0.0157 | 0.1687 | 1.42E-06 | 23.24837 |
|  |  | rs10242459 | 7 | 3911280 | A | G | 0.0729 | 0.0154 | 0.8219 | 2.20E-06 | 22.40854 |
|  |  | rs73257451 | 8 | 61418130 | T | C | -0.3645 | 0.0756 | 0.989 | 1.43E-06 | 23.24617 |
|  |  | rs11574915 | 9 | 34710084 | A | C | -0.1464 | 0.017 | 0.8639 | 7.19E-18 | 74.16249 |
|  |  | rs34791102 | 9 | 35612978 | A | G | 0.1238 | 0.0265 | 0.0534 | 2.99E-06 | 21.82476 |
|  |  | rs6486995 | 12 | 19813981 | A | T | -0.0697 | 0.0135 | 0.2452 | 2.43E-07 | 26.65619 |
|  |  | rs4766578 | 12 | 111904371 | A | T | -0.0801 | 0.0132 | 0.5095 | 1.29E-09 | 36.82283 |
|  |  | rs4907572 | 13 | 113647321 | A | G | -0.0671 | 0.0137 | 0.2653 | 9.69E-07 | 23.98854 |
|  |  | rs138108512 | 14 | 76855291 | A | G | -0.2939 | 0.0616 | 0.0119 | 1.83E-06 | 22.76343 |
|  |  | rs74764632 | 16 | 57300587 | A | T | 0.1349 | 0.0294 | 0.9557 | 4.47E-06 | 21.05374 |
|  |  | rs147708384 | 17 | 14918425 | T | C | -0.2707 | 0.0574 | 0.0151 | 2.40E-06 | 22.24092 |
|  |  | rs138440733 | 22 | 17731243 | A | G | -0.1724 | 0.036 | 0.0585 | 1.68E-06 | 22.93346 |
| C-X-C motif chemokine 1 levels | AD | rs863004 | 1 | 159176831 | T | C | 0.0661 | 0.0119 | 0.3809 | 2.78E-08 | 30.85382 |
|  |  | rs2122155 | 4 | 59689961 | A | G | -0.0538 | 0.0117 | 0.5637 | 4.26E-06 | 21.14428 |
|  |  | rs565208061 | 4 | 74717663 | T | C | 0.5003 | 0.0824 | 0.0138 | 1.27E-09 | 36.86435 |
|  |  | rs1366946 | 4 | 74738469 | T | C | -0.3134 | 0.0134 | 0.7638 | 5.65E-121 | 547.0013 |
|  |  | rs62311721 | 4 | 84005961 | T | C | -0.186 | 0.0407 | 0.9588 | 4.88E-06 | 20.88512 |
|  |  | rs77914035 | 6 | 54641812 | A | G | -0.1047 | 0.0206 | 0.8675 | 3.72E-07 | 25.83205 |
|  |  | rs67797811 | 9 | 4396102 | C | G | 0.0822 | 0.0179 | 0.1691 | 4.39E-06 | 21.08811 |
|  |  | rs12770839 | 10 | 65297720 | A | C | 0.0648 | 0.0126 | 0.321 | 2.71E-07 | 26.44898 |
|  |  | rs10734292 | 11 | 20486401 | A | G | 0.123 | 0.0267 | 0.067 | 4.09E-06 | 21.22207 |
|  |  | rs4474574 | 13 | 37854253 | T | C | -0.365 | 0.0779 | 0.0146 | 2.79E-06 | 21.95386 |
|  |  | rs2280441 | 15 | 81240669 | A | G | -0.1443 | 0.0312 | 0.0405 | 3.75E-06 | 21.39063 |
|  |  | rs11880837 | 19 | 14808453 | A | G | 0.0583 | 0.0127 | 0.3161 | 4.42E-06 | 21.07316 |
|  |  | rs2835916 | 21 | 39102421 | C | G | -0.0564 | 0.012 | 0.3876 | 2.60E-06 | 22.09 |
|  |  | rs188111670 | 22 | 16564036 | A | G | 0.0976 | 0.0208 | 0.3429 | 2.70E-06 | 22.01775 |
| Fms-related tyrosine kinase 3 ligand levels | AD | rs55633025 | 1 | 159890969 | A | G | 0.0725 | 0.0129 | 0.4738 | 1.91E-08 | 31.58614 |
|  |  | rs186975 | 2 | 8442736 | C | G | 0.0554 | 0.0119 | 0.382 | 3.23E-06 | 21.67333 |
|  |  | rs1866051 | 2 | 65602149 | T | C | -0.0746 | 0.0115 | 0.4771 | 8.76E-11 | 42.0806 |
|  |  | rs7624160 | 3 | 128381886 | T | G | -0.1061 | 0.0119 | 0.6093 | 4.84E-19 | 79.49446 |
|  |  | rs7625643 | 3 | 141150026 | A | G | 0.0611 | 0.0129 | 0.5605 | 2.18E-06 | 22.43381 |
|  |  | rs61792394 | 3 | 170851052 | A | G | -0.1569 | 0.0338 | 0.9624 | 3.45E-06 | 21.54827 |
|  |  | rs575588389 | 4 | 20519864 | A | G | -0.0904 | 0.0192 | 0.8501 | 2.50E-06 | 22.1684 |
|  |  | rs218265 | 4 | 55408999 | T | C | 0.0833 | 0.016 | 0.8361 | 1.93E-07 | 27.10504 |
|  |  | rs1277312 | 4 | 57902945 | A | T | 0.0539 | 0.0117 | 0.4587 | 4.09E-06 | 21.22295 |
|  |  | rs144317085 | 4 | 105806108 | A | T | 0.2206 | 0.0324 | 0.9647 | 9.85E-12 | 46.35761 |
|  |  | rs7726159 | 5 | 1282319 | A | C | -0.1191 | 0.0128 | 0.3326 | 1.34E-20 | 86.57721 |
|  |  | rs1379304 | 5 | 6289394 | T | C | 0.0603 | 0.0127 | 0.3741 | 2.05E-06 | 22.5438 |
|  |  | rs139721800 | 6 | 18758144 | T | C | 0.0959 | 0.0202 | 0.9089 | 2.06E-06 | 22.53899 |
|  |  | rs9381118 | 6 | 41985436 | T | C | 0.0643 | 0.0136 | 0.2577 | 2.27E-06 | 22.35343 |
|  |  | rs6453962 | 6 | 77907355 | T | C | -0.0559 | 0.0117 | 0.4699 | 1.77E-06 | 22.82716 |
|  |  | rs6925032 | 6 | 91008027 | A | C | 0.0676 | 0.0128 | 0.326 | 1.28E-07 | 27.8916 |
|  |  | rs28533776 | 7 | 3914756 | A | G | -0.0749 | 0.0147 | 0.1924 | 3.48E-07 | 25.96145 |
|  |  | rs192857133 | 9 | 243950 | T | C | 0.1812 | 0.0386 | 0.026 | 2.68E-06 | 22.03646 |
|  |  | rs57191857 | 9 | 10640144 | A | G | -0.1429 | 0.0299 | 0.9571 | 1.76E-06 | 22.84137 |
|  |  | rs12338549 | 9 | 94348687 | T | C | -0.0851 | 0.0186 | 0.1096 | 4.76E-06 | 20.93308 |
|  |  | rs185979634 | 10 | 101403323 | T | C | 0.1483 | 0.0306 | 0.9513 | 1.26E-06 | 23.48764 |
|  |  | rs74722523 | 10 | 105351013 | T | C | 0.3529 | 0.076 | 0.9914 | 3.43E-06 | 21.56136 |
|  |  | rs141117375 | 11 | 62294309 | T | C | -0.1519 | 0.0331 | 0.0364 | 4.45E-06 | 21.06006 |
|  |  | rs11212636 | 11 | 108311965 | A | G | -0.0772 | 0.0117 | 0.4171 | 4.16E-11 | 43.53744 |
|  |  | rs73000965 | 11 | 113982321 | A | T | 0.0588 | 0.0125 | 0.3111 | 2.55E-06 | 22.12762 |
|  |  | rs9554186 | 13 | 28455266 | A | T | 0.0821 | 0.0128 | 0.3602 | 1.42E-10 | 41.1402 |
|  |  | rs76428106 | 13 | 28604007 | T | C | -1.1019 | 0.0524 | 0.9832 | 3.59E-98 | 442.2031 |
|  |  | rs72681653 | 14 | 51156734 | A | G | 0.1302 | 0.0266 | 0.9447 | 9.84E-07 | 23.95845 |
|  |  | rs10164255 | 18 | 70532840 | T | G | 0.0556 | 0.0121 | 0.408 | 4.33E-06 | 21.1144 |
|  |  | rs17272847 | 19 | 49979398 | A | G | 0.1179 | 0.022 | 0.0907 | 8.36E-08 | 28.71986 |
|  |  | rs142870956 | 20 | 4261712 | T | C | -0.4307 | 0.0883 | 0.0075 | 1.07E-06 | 23.79186 |
|  |  | rs78429945 | 20 | 30439298 | T | G | 0.0884 | 0.016 | 0.1818 | 3.29E-08 | 30.52563 |
| Interleukin-18 levels | AD | rs385076 | 2 | 32489851 | T | C | -0.1857 | 0.0123 | 0.3734 | 1.68E-51 | 227.9363 |
|  |  | rs12711729 | 2 | 126751021 | T | C | -0.0702 | 0.0153 | 0.7336 | 4.47E-06 | 21.0519 |
|  |  | rs139760701 | 3 | 115001900 | A | T | -0.3049 | 0.0604 | 0.989 | 4.46E-07 | 25.48244 |
|  |  | rs12648080 | 4 | 121893301 | A | G | 0.072 | 0.0146 | 0.8077 | 8.16E-07 | 24.31976 |
|  |  | rs17229943 | 5 | 68682536 | A | C | -0.16 | 0.0267 | 0.9351 | 2.07E-09 | 35.91017 |
|  |  | rs567093451 | 5 | 70375337 | T | G | 0.1154 | 0.0252 | 0.8692 | 4.66E-06 | 20.97058 |
|  |  | rs4140444 | 6 | 38300402 | A | G | 0.2157 | 0.0461 | 0.0204 | 2.88E-06 | 21.89266 |
|  |  | rs7755771 | 6 | 158786921 | T | C | -0.0861 | 0.0181 | 0.2688 | 1.97E-06 | 22.62816 |
|  |  | rs12201187 | 6 | 162785078 | A | T | 0.0676 | 0.0138 | 0.2767 | 9.65E-07 | 23.9958 |
|  |  | rs67424072 | 7 | 11104167 | T | G | -0.082 | 0.0165 | 0.8341 | 6.71E-07 | 24.69789 |
|  |  | rs56836641 | 7 | 155637974 | T | C | -0.163 | 0.0301 | 0.0477 | 6.12E-08 | 29.32528 |
|  |  | rs2954016 | 8 | 126402485 | T | C | -0.0759 | 0.0156 | 0.3451 | 1.14E-06 | 23.67197 |
|  |  | rs12681624 | 8 | 144606172 | C | G | -0.0935 | 0.0157 | 0.8179 | 2.59E-09 | 35.46696 |
|  |  | rs112338272 | 9 | 20069041 | A | C | -0.1954 | 0.0425 | 0.0236 | 4.27E-06 | 21.13836 |
|  |  | rs11605127 | 11 | 268430 | C | G | -0.0629 | 0.0137 | 0.7543 | 4.41E-06 | 21.07949 |
|  |  | rs57954386 | 11 | 86686181 | C | G | -0.1248 | 0.0259 | 0.0665 | 1.45E-06 | 23.21826 |
|  |  | rs60235102 | 11 | 105060888 | T | C | 0.1154 | 0.0225 | 0.9237 | 2.91E-07 | 26.3055 |
|  |  | rs5744249 | 11 | 112025306 | A | C | 0.2039 | 0.0135 | 0.7618 | 1.53E-51 | 228.1219 |
|  |  | rs35495214 | 11 | 112433719 | T | C | 0.1374 | 0.0291 | 0.0465 | 2.34E-06 | 22.29397 |
|  |  | rs148504635 | 16 | 54161464 | A | C | 0.2532 | 0.0525 | 0.9857 | 1.42E-06 | 23.25995 |
|  |  | rs113552426 | 16 | 84730435 | C | G | -0.278 | 0.0581 | 0.9792 | 1.71E-06 | 22.89482 |
|  |  | rs77994606 | 18 | 28763724 | A | G | -0.2314 | 0.0472 | 0.9814 | 9.46E-07 | 24.03492 |
|  |  | rs2108621 | 19 | 5344989 | T | C | 0.0581 | 0.0126 | 0.3565 | 4.01E-06 | 21.26235 |
|  |  | rs1403742 | 20 | 51289816 | A | T | -0.0567 | 0.0124 | 0.371 | 4.82E-06 | 20.90849 |
| Leukemia inhibitory factor receptor levels | AD | rs12754806 | 1 | 238821453 | A | G | 0.0563 | 0.0122 | 0.4817 | 3.94E-06 | 21.29596 |
|  |  | rs3963364 | 3 | 12385357 | A | C | -0.0951 | 0.0203 | 0.1784 | 2.80E-06 | 21.94669 |
|  |  | rs62258077 | 3 | 58363371 | T | C | -0.063 | 0.013 | 0.6603 | 1.26E-06 | 23.48521 |
|  |  | rs9313148 | 5 | 6378192 | C | G | -0.0824 | 0.0173 | 0.1414 | 1.91E-06 | 22.68622 |
|  |  | rs72730728 | 5 | 36424241 | A | T | 0.1414 | 0.0302 | 0.9403 | 2.84E-06 | 21.92224 |
|  |  | rs2289779 | 5 | 38527228 | T | G | 0.0788 | 0.0134 | 0.5419 | 4.09E-09 | 34.58142 |
|  |  | rs115938660 | 5 | 110784241 | T | C | -0.2181 | 0.0467 | 0.0215 | 3.01E-06 | 21.8111 |
|  |  | rs778016 | 6 | 68918580 | A | G | -0.0681 | 0.0145 | 0.2875 | 2.65E-06 | 22.0576 |
|  |  | rs73157388 | 7 | 79349730 | T | C | -0.1306 | 0.0285 | 0.9229 | 4.60E-06 | 20.9989 |
|  |  | rs117117000 | 8 | 119917062 | A | G | 0.1586 | 0.0333 | 0.961 | 1.91E-06 | 22.68391 |
|  |  | rs635634 | 9 | 136155000 | T | C | -0.2885 | 0.0153 | 0.1918 | 2.61E-79 | 355.5566 |
|  |  | rs41307428 | 9 | 136336804 | T | C | 0.2027 | 0.0299 | 0.067 | 1.21E-11 | 45.95842 |
|  |  | rs1690678 | 10 | 27930818 | A | C | 0.0904 | 0.0192 | 0.1109 | 2.50E-06 | 22.1684 |
|  |  | rs4937122 | 11 | 126228659 | T | G | 0.1262 | 0.0248 | 0.9129 | 3.61E-07 | 25.89497 |
|  |  | rs72837682 | 17 | 7063650 | T | C | -0.08 | 0.0172 | 0.7807 | 3.30E-06 | 21.63332 |
|  |  | rs141933232 | 17 | 36964094 | A | G | -0.2724 | 0.0546 | 0.0169 | 6.07E-07 | 24.89023 |
|  |  | rs62654645 | 21 | 34695981 | T | G | -0.0812 | 0.0172 | 0.5221 | 2.35E-06 | 22.28718 |
|  |  | rs147006060 | 21 | 43780807 | A | G | -0.2602 | 0.0566 | 0.0179 | 4.28E-06 | 21.134 |
|  |  | rs6002001 | 22 | 41054967 | A | G | 0.6489 | 0.1417 | 0.0043 | 4.66E-06 | 20.97084 |
| Monocyte chemoattractant protein-1 levels | AD | rs12133241 | 1 | 14260220 | T | G | 0.0728 | 0.0157 | 0.8345 | 3.54E-06 | 21.50124 |
|  |  | rs12075 | 1 | 159175354 | A | G | 0.1694 | 0.0113 | 0.5552 | 8.39E-51 | 224.7346 |
|  |  | rs4682862 | 3 | 42877413 | A | G | 0.0576 | 0.0122 | 0.6154 | 2.34E-06 | 22.29078 |
|  |  | rs1846616 | 3 | 46032441 | A | T | 0.0856 | 0.0119 | 0.362 | 6.33E-13 | 51.74324 |
|  |  | rs35728689 | 3 | 46390228 | A | G | 0.3004 | 0.0198 | 0.0959 | 5.44E-52 | 230.181 |
|  |  | rs181998213 | 3 | 46519788 | T | C | 0.3723 | 0.0695 | 0.9872 | 8.47E-08 | 28.69568 |
|  |  | rs7619350 | 3 | 132269435 | A | G | 0.0986 | 0.0201 | 0.118 | 9.32E-07 | 24.06366 |
|  |  | rs149604038 | 3 | 185463545 | C | G | -0.2813 | 0.0556 | 0.9851 | 4.21E-07 | 25.59705 |
|  |  | rs207311 | 4 | 19075878 | T | C | 0.0579 | 0.0126 | 0.6697 | 4.32E-06 | 21.11621 |
|  |  | rs148307902 | 5 | 2885014 | A | G | 0.2553 | 0.0538 | 0.0243 | 2.08E-06 | 22.51838 |
|  |  | rs146186562 | 6 | 20408729 | T | G | -0.2293 | 0.0497 | 0.9789 | 3.96E-06 | 21.28606 |
|  |  | rs9942602 | 7 | 17977054 | T | C | 0.0618 | 0.0117 | 0.4922 | 1.28E-07 | 27.90007 |
|  |  | rs182599222 | 8 | 115072929 | A | T | -0.4028 | 0.0864 | 0.991 | 3.13E-06 | 21.73459 |
|  |  | rs77344300 | 9 | 78838039 | T | C | -0.1381 | 0.0293 | 0.0491 | 2.44E-06 | 22.2153 |
|  |  | rs117510656 | 11 | 87277670 | T | C | -0.2587 | 0.0562 | 0.9806 | 4.16E-06 | 21.18948 |
|  |  | rs12367122 | 12 | 30998369 | A | G | 0.0739 | 0.0159 | 0.1756 | 3.35E-06 | 21.60203 |
|  |  | rs34292320 | 13 | 43553825 | T | C | -0.0684 | 0.0134 | 0.2742 | 3.32E-07 | 26.05569 |
|  |  | rs11639226 | 15 | 91387220 | A | C | 0.0615 | 0.0133 | 0.4782 | 3.76E-06 | 21.38193 |
|  |  | rs117265389 | 16 | 4938452 | C | G | 0.1734 | 0.0377 | 0.0256 | 4.24E-06 | 21.15512 |
|  |  | rs11870164 | 17 | 32567679 | T | C | 0.0751 | 0.013 | 0.2772 | 7.61E-09 | 33.37284 |
|  |  | rs190108694 | 18 | 75133468 | A | G | -0.2084 | 0.0455 | 0.0286 | 4.64E-06 | 20.97841 |
|  |  | rs79697698 | 20 | 9086080 | T | C | -0.348 | 0.0653 | 0.0136 | 9.86E-08 | 28.4009 |
|  |  | rs77128226 | 20 | 15213518 | T | C | -0.1063 | 0.0218 | 0.0875 | 1.08E-06 | 23.77681 |
|  |  | rs117317477 | 22 | 21921920 | T | G | -0.3227 | 0.0704 | 0.0125 | 4.57E-06 | 21.01128 |
| SIR2-like protein 2 levels | AD | rs138897138 | 2 | 122731427 | A | G | 0.104 | 0.0218 | 0.9138 | 1.84E-06 | 22.75903 |
|  |  | rs116198125 | 3 | 14613224 | T | C | -0.1521 | 0.0329 | 0.9617 | 3.78E-06 | 21.37306 |
|  |  | rs60459634 | 4 | 160629137 | A | G | -0.1063 | 0.0229 | 0.0886 | 3.45E-06 | 21.54743 |
|  |  | rs115455827 | 5 | 78787853 | A | G | 0.1277 | 0.0277 | 0.9506 | 4.02E-06 | 21.2531 |
|  |  | rs7718478 | 5 | 177560339 | A | T | 0.0639 | 0.0138 | 0.7496 | 3.65E-06 | 21.44093 |
|  |  | rs72883370 | 6 | 63413043 | A | G | -0.3428 | 0.0737 | 0.989 | 3.30E-06 | 21.63449 |
|  |  | rs1034059 | 6 | 158193698 | A | G | 0.076 | 0.0145 | 0.6981 | 1.59E-07 | 27.47206 |
|  |  | rs12531843 | 7 | 14143486 | C | G | -0.1286 | 0.0243 | 0.9177 | 1.21E-07 | 28.00718 |
|  |  | rs147383134 | 8 | 89590527 | T | C | 0.2573 | 0.0551 | 0.9858 | 3.02E-06 | 21.80602 |
|  |  | rs11115639 | 12 | 83600525 | A | C | -0.0841 | 0.0184 | 0.8864 | 4.86E-06 | 20.89086 |
|  |  | rs77381020 | 13 | 37413465 | T | C | 0.0862 | 0.0184 | 0.8546 | 2.80E-06 | 21.94719 |
|  |  | rs144373891 | 19 | 39379770 | T | C | -0.5739 | 0.0515 | 0.0161 | 7.69E-29 | 124.1818 |
|  |  | rs12152184 | 22 | 45002809 | A | G | 0.0965 | 0.0208 | 0.885 | 3.49E-06 | 21.52425 |
| T-cell surface glycoprotein CD5 levels | ALS | rs61768480 | 1 | 1103150 | A | G | 0.0695 | 0.0147 | 0.2776 | 2.27E-06 | 22.35295 |
|  |  | rs61747728 | 1 | 179526214 | T | C | 0.1841 | 0.0313 | 0.0399 | 4.06E-09 | 34.59544 |
|  |  | rs13010492 | 2 | 204776176 | C | G | -0.0589 | 0.0122 | 0.4754 | 1.38E-06 | 23.30832 |
|  |  | rs563284470 | 3 | 47239457 | A | G | 0.1833 | 0.0391 | 0.9694 | 2.76E-06 | 21.97715 |
|  |  | rs138861398 | 4 | 77211819 | A | C | -0.0803 | 0.0151 | 0.2321 | 1.05E-07 | 28.27986 |
|  |  | rs74874989 | 4 | 164762615 | A | G | 0.141 | 0.0292 | 0.1033 | 1.37E-06 | 23.31699 |
|  |  | rs4073745 | 5 | 176733341 | A | G | -0.0683 | 0.0131 | 0.7003 | 1.85E-07 | 27.18309 |
|  |  | rs72821405 | 6 | 4714797 | T | C | -0.1014 | 0.0217 | 0.0833 | 2.97E-06 | 21.83516 |
|  |  | rs527506034 | 6 | 32020238 | A | G | 0.0832 | 0.016 | 0.8464 | 1.99E-07 | 27.04 |
|  |  | rs3104373 | 6 | 32600375 | T | C | 0.0973 | 0.0165 | 0.1469 | 3.70E-09 | 34.77425 |
|  |  | rs2237450 | 7 | 50716017 | T | C | 0.0623 | 0.0124 | 0.6389 | 5.06E-07 | 25.24252 |
|  |  | rs17345759 | 7 | 97499410 | A | C | -0.1134 | 0.0245 | 0.9301 | 3.68E-06 | 21.42367 |
|  |  | rs17769062 | 9 | 27496604 | T | C | 0.1638 | 0.0334 | 0.9589 | 9.38E-07 | 24.0511 |
|  |  | rs192582121 | 9 | 105614669 | A | T | -0.4169 | 0.0806 | 0.9916 | 2.31E-07 | 26.75431 |
|  |  | rs117276991 | 10 | 6251691 | A | G | -0.1261 | 0.0256 | 0.0751 | 8.40E-07 | 24.26332 |
|  |  | rs12416520 | 10 | 71211945 | A | G | -0.0629 | 0.0132 | 0.7492 | 1.89E-06 | 22.70667 |
|  |  | rs60562286 | 10 | 114454187 | A | T | -0.1681 | 0.0352 | 0.0327 | 1.79E-06 | 22.80605 |
|  |  | rs4910962 | 11 | 4281522 | A | C | 0.1465 | 0.0312 | 0.9341 | 2.66E-06 | 22.04784 |
|  |  | rs4939490 | 11 | 60793651 | C | G | 0.1083 | 0.012 | 0.6328 | 1.80E-19 | 81.45063 |
|  |  | rs674379 | 11 | 60922561 | C | G | 0.1193 | 0.0126 | 0.6395 | 2.85E-21 | 89.64783 |
|  |  | rs1259355 | 12 | 31688535 | A | G | 0.0569 | 0.0119 | 0.4361 | 1.74E-06 | 22.86286 |
|  |  | rs3184504 | 12 | 111884608 | T | C | 0.1427 | 0.0128 | 0.4764 | 7.29E-29 | 124.2877 |
|  |  | rs117516652 | 13 | 39003323 | A | G | 0.2374 | 0.0476 | 0.9826 | 6.12E-07 | 24.87411 |
|  |  | rs11622090 | 14 | 93329692 | T | C | -0.0588 | 0.0128 | 0.3206 | 4.35E-06 | 21.10254 |
|  |  | rs7242462 | 18 | 45545720 | A | G | -0.1199 | 0.0187 | 0.8853 | 1.44E-10 | 41.11073 |
|  |  | rs4893 | 19 | 48833608 | A | G | 0.1471 | 0.0313 | 0.9627 | 2.61E-06 | 22.087 |
| C-X-C motif chemokine 10 levels | ALS | rs112202639 | 1 | 44566391 | A | G | -0.1609 | 0.0336 | 0.9671 | 1.68E-06 | 22.93156 |
|  |  | rs1563171 | 2 | 3891773 | A | G | 0.0592 | 0.0127 | 0.7042 | 3.14E-06 | 21.72881 |
|  |  | rs12476448 | 2 | 14400474 | T | C | 0.0536 | 0.0115 | 0.4864 | 3.15E-06 | 21.72371 |
|  |  | rs12629593 | 3 | 54104178 | T | G | -0.0879 | 0.0182 | 0.1209 | 1.37E-06 | 23.32572 |
|  |  | rs72651343 | 4 | 76808216 | T | C | -0.6096 | 0.0619 | 0.9892 | 6.98E-23 | 96.9859 |
|  |  | rs115140093 | 4 | 76910673 | T | C | -0.5186 | 0.042 | 0.9732 | 5.02E-35 | 152.4637 |
|  |  | rs12646113 | 4 | 77175245 | T | C | 0.0977 | 0.0136 | 0.246 | 6.78E-13 | 51.60732 |
|  |  | rs72682331 | 4 | 112904535 | A | G | 0.1089 | 0.0209 | 0.0979 | 1.88E-07 | 27.14958 |
|  |  | rs2523495 | 6 | 31377978 | T | C | 0.1095 | 0.0196 | 0.1048 | 2.31E-08 | 31.2116 |
|  |  | rs71534595 | 6 | 32516855 | T | C | -0.2991 | 0.0606 | 0.9813 | 7.99E-07 | 24.36058 |
|  |  | rs12174864 | 6 | 88557407 | A | G | -0.0582 | 0.0121 | 0.6254 | 1.51E-06 | 23.1353 |
|  |  | rs12155428 | 7 | 3425301 | T | C | -0.1765 | 0.0375 | 0.0284 | 2.52E-06 | 22.15271 |
|  |  | rs2349689 | 8 | 62003730 | A | G | -0.0789 | 0.0164 | 0.2555 | 1.50E-06 | 23.14549 |
|  |  | rs143796249 | 9 | 4150061 | C | G | -0.2858 | 0.062 | 0.012 | 4.03E-06 | 21.24913 |
|  |  | rs10481754 | 9 | 84196490 | T | C | -0.0548 | 0.0119 | 0.4567 | 4.12E-06 | 21.20641 |
|  |  | rs4367871 | 10 | 1845059 | T | C | -0.0567 | 0.0119 | 0.5587 | 1.89E-06 | 22.70242 |
|  |  | rs12779465 | 10 | 78705575 | A | C | -0.0698 | 0.015 | 0.2969 | 3.27E-06 | 21.65351 |
|  |  | rs55732300 | 11 | 73731422 | A | G | 0.1175 | 0.025 | 0.929 | 2.60E-06 | 22.09 |
|  |  | rs741344 | 12 | 68597086 | A | G | -0.06 | 0.013 | 0.7036 | 3.92E-06 | 21.30178 |
|  |  | rs3184504 | 12 | 111884608 | T | C | 0.1138 | 0.0131 | 0.4756 | 3.72E-18 | 75.46437 |
|  |  | rs1077965 | 15 | 80065337 | A | G | -0.0546 | 0.0118 | 0.5648 | 3.71E-06 | 21.41023 |
|  |  | rs74956615 | 19 | 10427721 | A | T | -0.1358 | 0.0294 | 0.0495 | 3.86E-06 | 21.3356 |
|  |  | rs4804148 | 19 | 11270867 | T | C | -0.0629 | 0.013 | 0.7227 | 1.31E-06 | 23.41071 |
|  |  | rs150519056 | 22 | 50774821 | T | C | -0.1841 | 0.0376 | 0.9688 | 9.77E-07 | 23.97352 |
| C-X-C motif chemokine 11 levels | ALS | rs60141220 | 1 | 26395741 | A | G | -0.0777 | 0.0165 | 0.1703 | 2.49E-06 | 22.17554 |
|  |  | rs62291421 | 3 | 188015332 | C | G | 0.2674 | 0.0518 | 0.9763 | 2.44E-07 | 26.64792 |
|  |  | rs6827617 | 4 | 76916146 | A | G | -0.1724 | 0.0118 | 0.6041 | 2.42E-48 | 213.4571 |
|  |  | rs59148364 | 4 | 77130802 | A | G | 0.1821 | 0.0378 | 0.0278 | 1.45E-06 | 23.20792 |
|  |  | rs116470923 | 4 | 77374393 | T | C | -0.1975 | 0.0427 | 0.0216 | 3.74E-06 | 21.39333 |
|  |  | rs112884138 | 4 | 134362360 | T | C | -0.0976 | 0.0209 | 0.0892 | 3.01E-06 | 21.80756 |
|  |  | rs72747175 | 5 | 18009856 | A | G | 0.1535 | 0.0326 | 0.9608 | 2.49E-06 | 22.17081 |
|  |  | rs2894233 | 6 | 32011678 | A | G | -0.2236 | 0.047 | 0.9724 | 1.96E-06 | 22.6333 |
|  |  | rs72925566 | 6 | 89498971 | A | G | -0.0973 | 0.0185 | 0.1651 | 1.44E-07 | 27.66191 |
|  |  | rs7750544 | 6 | 154177484 | T | C | -0.0773 | 0.0164 | 0.165 | 2.44E-06 | 22.21628 |
|  |  | rs75612655 | 7 | 101594247 | T | C | 0.5352 | 0.0705 | 0.0084 | 3.16E-14 | 57.63071 |
|  |  | rs111787182 | 7 | 157911633 | A | C | -0.1445 | 0.0314 | 0.951 | 4.19E-06 | 21.17758 |
|  |  | rs78632565 | 8 | 70982905 | T | C | 0.1082 | 0.0214 | 0.9152 | 4.28E-07 | 25.56389 |
|  |  | rs7009937 | 8 | 96176796 | T | C | 0.1175 | 0.025 | 0.9321 | 2.60E-06 | 22.09 |
|  |  | rs4748380 | 10 | 17590681 | T | C | 0.0901 | 0.019 | 0.1188 | 2.12E-06 | 22.48756 |
|  |  | rs10733789 | 10 | 64948684 | T | C | -0.0815 | 0.0127 | 0.6875 | 1.39E-10 | 41.18203 |
|  |  | rs7475335 | 10 | 104343428 | A | C | -0.0646 | 0.0124 | 0.3172 | 1.89E-07 | 27.14074 |
|  |  | rs111848253 | 11 | 22351553 | A | G | -0.1162 | 0.025 | 0.9044 | 3.35E-06 | 21.6039 |
|  |  | rs10898158 | 11 | 83554112 | A | G | 0.0609 | 0.0121 | 0.6028 | 4.83E-07 | 25.33167 |
|  |  | rs3184504 | 12 | 111884608 | T | C | 0.1236 | 0.0132 | 0.4764 | 7.70E-21 | 87.67769 |
|  |  | rs529862022 | 14 | 30090297 | A | C | 0.1254 | 0.0274 | 0.1092 | 4.72E-06 | 20.94566 |
|  |  | rs12433317 | 14 | 50775476 | A | G | 0.0571 | 0.012 | 0.4282 | 1.95E-06 | 22.64174 |
|  |  | rs7222922 | 17 | 34335694 | T | C | -0.0882 | 0.0182 | 0.1205 | 1.26E-06 | 23.48521 |
|  |  | rs2245786 | 20 | 4795382 | A | G | -0.0599 | 0.0131 | 0.6525 | 4.82E-06 | 20.90793 |
|  |  | rs191536201 | 20 | 17839484 | A | G | -0.3323 | 0.0684 | 0.0117 | 1.18E-06 | 23.60198 |
| Hepatocyte growth factor levels | ALS | rs34219417 | 1 | 38949303 | T | C | 0.0992 | 0.0217 | 0.8959 | 4.84E-06 | 20.89796 |
|  |  | rs138848271 | 1 | 83864840 | T | C | -0.405 | 0.0832 | 0.9907 | 1.13E-06 | 23.69536 |
|  |  | rs6709159 | 2 | 108946178 | A | G | 0.0564 | 0.0114 | 0.4434 | 7.52E-07 | 24.47645 |
|  |  | rs2737756 | 3 | 128064791 | A | G | 0.2047 | 0.0391 | 0.0276 | 1.65E-07 | 27.4083 |
|  |  | rs7620568 | 3 | 171934440 | A | G | -0.0856 | 0.0179 | 0.1397 | 1.73E-06 | 22.8687 |
|  |  | rs59950280 | 4 | 3452345 | A | G | 0.1219 | 0.0131 | 0.328 | 1.34E-20 | 86.58942 |
|  |  | rs183596950 | 4 | 31819358 | A | G | -0.0699 | 0.0152 | 0.6438 | 4.25E-06 | 21.1479 |
|  |  | rs115009171 | 6 | 622484 | A | G | 0.4297 | 0.0934 | 0.0078 | 4.21E-06 | 21.16591 |
|  |  | rs201840950 | 6 | 32487091 | T | G | 0.0993 | 0.0215 | 0.7957 | 3.86E-06 | 21.33151 |
|  |  | rs5745692 | 7 | 81358266 | C | G | -0.1545 | 0.0332 | 0.964 | 3.26E-06 | 21.65613 |
|  |  | rs5745687 | 7 | 81359051 | T | C | -0.1943 | 0.0242 | 0.0608 | 9.83E-16 | 64.46365 |
|  |  | rs113903858 | 8 | 72362254 | A | G | 0.3666 | 0.0747 | 0.014 | 9.22E-07 | 24.08484 |
|  |  | rs5007364 | 9 | 107570130 | T | C | 0.0769 | 0.0164 | 0.7969 | 2.75E-06 | 21.98695 |
|  |  | rs74130123 | 10 | 30042437 | T | C | -0.2429 | 0.0489 | 0.9809 | 6.79E-07 | 24.67387 |
|  |  | rs57193877 | 14 | 65654346 | T | C | 0.1572 | 0.0311 | 0.9611 | 4.31E-07 | 25.54961 |
|  |  | rs11631440 | 15 | 101649383 | A | T | -0.0638 | 0.012 | 0.6021 | 1.06E-07 | 28.26694 |
|  |  | rs4290523 | 17 | 7014969 | T | C | -0.0992 | 0.0217 | 0.0942 | 4.84E-06 | 20.89796 |
|  |  | rs1986719 | 17 | 45959996 | T | C | -0.1954 | 0.039 | 0.9572 | 5.44E-07 | 25.10267 |
|  |  | rs150498795 | 18 | 76506666 | A | T | 0.1566 | 0.0327 | 0.9642 | 1.68E-06 | 22.93443 |
|  |  | rs851612 | 19 | 42769693 | A | G | -0.1182 | 0.0257 | 0.9405 | 4.24E-06 | 21.15284 |
|  |  | rs8106345 | 19 | 58888317 | A | G | 0.0592 | 0.0127 | 0.5179 | 3.14E-06 | 21.72881 |
|  |  | rs2281532 | 20 | 6103587 | T | C | -0.0698 | 0.0151 | 0.7983 | 3.79E-06 | 21.36766 |
| Interleukin-2 receptor subunit beta levels | ALS | rs13001788 | 2 | 31896839 | T | C | 0.2769 | 0.0557 | 0.9745 | 6.65E-07 | 24.71357 |
|  |  | rs115890206 | 2 | 171243489 | A | G | 0.232 | 0.0502 | 0.9786 | 3.81E-06 | 21.35839 |
|  |  | rs17549820 | 4 | 108118301 | T | C | 0.0631 | 0.0129 | 0.5622 | 1.00E-06 | 23.92651 |
|  |  | rs116007066 | 4 | 136755243 | A | G | -0.2101 | 0.0441 | 0.025 | 1.90E-06 | 22.69734 |
|  |  | rs9321271 | 6 | 131393771 | A | C | -0.157 | 0.0325 | 0.9549 | 1.36E-06 | 23.33633 |
|  |  | rs1472270 | 7 | 98121843 | C | G | 0.0704 | 0.0144 | 0.346 | 1.01E-06 | 23.90123 |
|  |  | rs34246740 | 11 | 36198233 | T | C | -0.0721 | 0.0156 | 0.2619 | 3.80E-06 | 21.36099 |
|  |  | rs141460846 | 11 | 40470032 | T | C | -0.2471 | 0.0535 | 0.9783 | 3.86E-06 | 21.33231 |
|  |  | rs56353341 | 12 | 13099229 | T | C | -0.0984 | 0.021 | 0.7599 | 2.79E-06 | 21.95592 |
|  |  | rs2157876 | 12 | 111592380 | T | G | -0.1133 | 0.0239 | 0.0879 | 2.13E-06 | 22.47315 |
|  |  | rs559991994 | 13 | 106109408 | A | T | 0.3764 | 0.0809 | 0.0166 | 3.28E-06 | 21.64722 |
|  |  | rs11630708 | 15 | 84665328 | T | C | -0.2102 | 0.0443 | 0.9766 | 2.09E-06 | 22.51428 |
|  |  | rs9921181 | 16 | 80775714 | A | G | -0.0606 | 0.0128 | 0.4465 | 2.20E-06 | 22.41431 |
|  |  | rs117538197 | 17 | 4507431 | T | C | 0.1213 | 0.0261 | 0.0695 | 3.36E-06 | 21.59935 |
| Transforming growth factor-alpha levels | ALS | rs78912149 | 1 | 166175871 | C | G | -0.2692 | 0.056 | 0.9835 | 1.53E-06 | 23.10862 |
|  |  | rs72912115 | 2 | 70774295 | A | T | 0.137 | 0.0184 | 0.114 | 9.65E-14 | 55.43774 |
|  |  | rs34007466 | 4 | 16165036 | A | G | -0.2684 | 0.05 | 0.0153 | 7.96E-08 | 28.81542 |
|  |  | rs11242125 | 5 | 132025905 | A | C | -0.0761 | 0.0166 | 0.7246 | 4.55E-06 | 21.01615 |
|  |  | rs9262670 | 6 | 31034990 | T | C | 0.0717 | 0.0146 | 0.2343 | 9.06E-07 | 24.11752 |
|  |  | rs1963491 | 8 | 2654153 | C | G | 0.1081 | 0.023 | 0.905 | 2.60E-06 | 22.09 |
|  |  | rs34630685 | 8 | 13724808 | C | G | 0.1678 | 0.0361 | 0.9681 | 3.35E-06 | 21.60576 |
|  |  | rs74130123 | 10 | 30042437 | T | C | -0.2601 | 0.0508 | 0.9817 | 3.05E-07 | 26.21521 |
|  |  | rs4934441 | 10 | 90836825 | A | G | -0.095 | 0.0198 | 0.1322 | 1.60E-06 | 23.02061 |
|  |  | rs12270510 | 11 | 119506018 | T | C | 0.0959 | 0.019 | 0.1107 | 4.48E-07 | 25.47593 |
|  |  | rs653178 | 12 | 112007756 | T | C | -0.0708 | 0.0131 | 0.5228 | 6.50E-08 | 29.20949 |
|  |  | rs3859189 | 17 | 38137033 | A | G | 0.0655 | 0.0116 | 0.5369 | 1.64E-08 | 31.88355 |
|  |  | rs111613293 | 17 | 52902972 | A | T | -0.0581 | 0.0122 | 0.4071 | 1.91E-06 | 22.67945 |
|  |  | rs78464136 | 19 | 15885492 | A | T | 0.143 | 0.0307 | 0.9552 | 3.19E-06 | 21.69678 |
|  |  | rs149146289 | 19 | 58857883 | A | G | 0.2856 | 0.0569 | 0.9866 | 5.19E-07 | 25.1937 |
| Tumor necrosis factor ligand superfamily member 12 levels | ALS | rs587680479 | 1 | 146403678 | A | C | 0.2346 | 0.0505 | 0.9586 | 3.39E-06 | 21.58108 |
|  |  | rs12145644 | 1 | 162517994 | A | T | -0.0727 | 0.0141 | 0.2484 | 2.52E-07 | 26.58463 |
|  |  | rs2738752 | 1 | 221052765 | A | G | -0.0591 | 0.0129 | 0.6653 | 4.62E-06 | 20.98918 |
|  |  | rs12726054 | 1 | 233623860 | A | G | 0.109 | 0.0228 | 0.0734 | 1.75E-06 | 22.85511 |
|  |  | rs2163333 | 2 | 53077754 | A | G | 0.1404 | 0.0273 | 0.9522 | 2.71E-07 | 26.44898 |
|  |  | rs200255093 | 2 | 99371603 | A | T | -0.0849 | 0.0168 | 0.8184 | 4.34E-07 | 25.53858 |
|  |  | rs826688 | 2 | 109239869 | A | T | -0.0592 | 0.0125 | 0.3965 | 2.18E-06 | 22.4297 |
|  |  | rs3821473 | 3 | 59625877 | A | G | -0.0622 | 0.0136 | 0.2327 | 4.80E-06 | 20.91717 |
|  |  | rs73133996 | 3 | 98429219 | C | G | 0.0812 | 0.012 | 0.4558 | 1.32E-11 | 45.78778 |
|  |  | rs1965399 | 3 | 142705695 | A | G | -0.1312 | 0.028 | 0.9544 | 2.79E-06 | 21.95592 |
|  |  | rs9842051 | 3 | 143021856 | C | G | 0.1192 | 0.014 | 0.748 | 1.68E-17 | 72.49306 |
|  |  | rs78254232 | 3 | 174384187 | T | C | -0.2108 | 0.0456 | 0.9813 | 3.79E-06 | 21.37034 |
|  |  | rs13107325 | 4 | 103188709 | T | C | 0.2027 | 0.0236 | 0.0696 | 8.77E-18 | 73.77063 |
|  |  | rs11738159 | 5 | 167311493 | T | C | 0.0577 | 0.0119 | 0.4339 | 1.24E-06 | 23.51027 |
|  |  | rs575752535 | 6 | 70682566 | T | C | -0.2262 | 0.0471 | 0.025 | 1.57E-06 | 23.06447 |
|  |  | rs16879825 | 6 | 88516736 | A | G | -0.2027 | 0.0438 | 0.9577 | 3.69E-06 | 21.41703 |
|  |  | rs11136339 | 8 | 145013345 | T | C | 0.0613 | 0.0122 | 0.388 | 5.05E-07 | 25.24651 |
|  |  | rs56023600 | 9 | 5636355 | T | C | -0.0856 | 0.0186 | 0.8764 | 4.18E-06 | 21.17979 |
|  |  | rs7019909 | 9 | 33113322 | T | C | -0.0897 | 0.0193 | 0.1102 | 3.36E-06 | 21.60082 |
|  |  | rs579459 | 9 | 136154168 | T | C | 0.1098 | 0.0157 | 0.7888 | 2.68E-12 | 48.91087 |
|  |  | rs201375822 | 10 | 17895807 | T | C | 0.1082 | 0.0193 | 0.2616 | 2.07E-08 | 31.42968 |
|  |  | rs111641307 | 10 | 51775971 | C | G | -0.401 | 0.0871 | 0.9859 | 4.15E-06 | 21.19593 |
|  |  | rs76047473 | 10 | 132815955 | T | C | 0.1318 | 0.0286 | 0.0482 | 4.06E-06 | 21.23727 |
|  |  | rs10831650 | 11 | 11644391 | A | G | 0.0692 | 0.0125 | 0.3558 | 3.09E-08 | 30.6473 |
|  |  | rs58743186 | 11 | 130975896 | A | G | -0.0717 | 0.0154 | 0.1931 | 3.23E-06 | 21.67688 |
|  |  | rs73590361 | 12 | 592399 | T | C | -0.0744 | 0.0161 | 0.1517 | 3.82E-06 | 21.35473 |
|  |  | rs117550445 | 12 | 82487578 | C | G | -0.1855 | 0.0377 | 0.9706 | 8.64E-07 | 24.21058 |
|  |  | rs4882710 | 12 | 129101566 | T | C | 0.0666 | 0.0131 | 0.3457 | 3.70E-07 | 25.84675 |
|  |  | rs552941568 | 14 | 55351508 | A | C | 0.1015 | 0.0218 | 0.1166 | 3.22E-06 | 21.67799 |
|  |  | rs34790908 | 17 | 7451110 | T | C | 0.211 | 0.0129 | 0.2563 | 3.90E-60 | 267.538 |
|  |  | rs7503751 | 17 | 7789542 | A | G | -0.1386 | 0.0238 | 0.0779 | 5.76E-09 | 33.91349 |
|  |  | rs76133364 | 19 | 19706214 | A | C | 0.1896 | 0.0369 | 0.0315 | 2.77E-07 | 26.40122 |
| Adenosine Deaminase levels | MS | rs115657851 | 1 | 57353848 | A | G | 0.139 | 0.0293 | 0.0462 | 2.10E-06 | 22.5058 |
|  |  | rs75492374 | 1 | 150902178 | A | G | 0.3552 | 0.077 | 0.9905 | 3.97E-06 | 21.27965 |
|  |  | rs541944924 | 1 | 204667577 | T | C | 0.1673 | 0.0358 | 0.0316 | 2.97E-06 | 21.83865 |
|  |  | rs111478357 | 2 | 144650434 | T | C | 0.2044 | 0.044 | 0.0243 | 3.39E-06 | 21.58025 |
|  |  | rs10024178 | 4 | 5801734 | T | C | -0.0925 | 0.0199 | 0.905 | 3.35E-06 | 21.60615 |
|  |  | rs189104742 | 4 | 75665562 | A | T | 0.2754 | 0.0584 | 0.9857 | 2.41E-06 | 22.23833 |
|  |  | rs62319741 | 4 | 124571895 | T | C | 0.1281 | 0.0258 | 0.9456 | 6.87E-07 | 24.65238 |
|  |  | rs1608554 | 7 | 22807634 | A | G | 0.0598 | 0.0122 | 0.656 | 9.50E-07 | 24.02607 |
|  |  | rs1636592 | 7 | 80681949 | A | T | -0.0562 | 0.0122 | 0.4024 | 4.09E-06 | 21.22037 |
|  |  | rs4749761 | 10 | 8502517 | A | T | 0.5548 | 0.1204 | 0.0104 | 4.07E-06 | 21.23342 |
|  |  | rs2620728 | 12 | 65118323 | C | G | -0.0551 | 0.0119 | 0.5267 | 3.65E-06 | 21.43923 |
|  |  | rs117645472 | 18 | 917376 | A | T | 0.3494 | 0.0754 | 0.0125 | 3.59E-06 | 21.47351 |
|  |  | rs74673238 | 20 | 43245735 | T | G | -0.9358 | 0.022 | 0.9326 | 0 | 1809.342 |
|  |  | rs2007720 | 20 | 43276688 | A | G | -0.1698 | 0.0136 | 0.7345 | 8.98E-36 | 155.8826 |
|  |  | rs74174913 | 20 | 43300819 | A | G | -0.1326 | 0.017 | 0.2254 | 6.19E-15 | 60.84 |
|  |  | rs7292320 | 22 | 37921425 | A | G | -0.0599 | 0.0131 | 0.2529 | 4.82E-06 | 20.90793 |
| Natural killer cell receptor 2B4 levels | MS | rs10903115 | 1 | 25261696 | A | G | -0.0559 | 0.0122 | 0.4429 | 4.61E-06 | 20.99442 |
|  |  | rs3828139 | 1 | 44253015 | T | C | 0.0744 | 0.0119 | 0.4677 | 4.05E-10 | 39.08876 |
|  |  | rs147033439 | 1 | 88677329 | A | G | 0.2053 | 0.0411 | 0.0249 | 5.88E-07 | 24.95136 |
|  |  | rs11265488 | 1 | 160773957 | A | G | -0.0624 | 0.0118 | 0.4675 | 1.24E-07 | 27.96438 |
|  |  | rs11265493 | 1 | 160803802 | A | G | 0.2122 | 0.0119 | 0.4066 | 3.99E-71 | 317.9778 |
|  |  | rs4672486 | 2 | 62495209 | A | G | 0.0661 | 0.0117 | 0.4329 | 1.61E-08 | 31.91767 |
|  |  | rs1434219 | 2 | 79554556 | T | C | -0.0589 | 0.0123 | 0.4388 | 1.68E-06 | 22.93086 |
|  |  | rs56272634 | 2 | 111846068 | T | C | 0.0704 | 0.0126 | 0.6994 | 2.31E-08 | 31.21794 |
|  |  | rs1574847 | 4 | 27611759 | C | G | -0.0637 | 0.0139 | 0.2366 | 4.59E-06 | 21.00145 |
|  |  | rs114758826 | 4 | 174530615 | A | G | -0.1804 | 0.0337 | 0.0393 | 8.65E-08 | 28.65585 |
|  |  | rs78142752 | 5 | 96078542 | T | C | 0.2288 | 0.0497 | 0.0184 | 4.15E-06 | 21.19333 |
|  |  | rs6928352 | 6 | 614064 | A | G | -0.1074 | 0.0215 | 0.0794 | 5.87E-07 | 24.95351 |
|  |  | rs2554697 | 8 | 3921805 | A | G | 0.0627 | 0.0134 | 0.6347 | 2.88E-06 | 21.89402 |
|  |  | rs34826779 | 8 | 106570964 | T | G | 0.063 | 0.0138 | 0.248 | 4.99E-06 | 20.84121 |
|  |  | rs10992054 | 9 | 92856642 | T | C | -0.3705 | 0.0763 | 0.9873 | 1.20E-06 | 23.57911 |
|  |  | rs11020961 | 11 | 94575608 | A | G | 0.0768 | 0.0161 | 0.8167 | 1.84E-06 | 22.75468 |
|  |  | rs11220506 | 11 | 126335329 | A | G | -0.0702 | 0.0147 | 0.1876 | 1.79E-06 | 22.8055 |
|  |  | rs3184504 | 12 | 111884608 | T | C | 0.116 | 0.0128 | 0.4764 | 1.27E-19 | 82.12891 |
|  |  | rs149458216 | 16 | 48902293 | A | C | -0.2146 | 0.047 | 0.0182 | 4.97E-06 | 20.84797 |
|  |  | rs72837690 | 17 | 7088423 | T | C | -0.0776 | 0.0125 | 0.6405 | 5.37E-10 | 38.53926 |
|  |  | rs139924614 | 22 | 17214005 | A | G | 0.1618 | 0.0348 | 0.9666 | 3.33E-06 | 21.61716 |
| CD40L receptor levels | MS | rs4278412 | 1 | 211450157 | C | G | 0.1978 | 0.0413 | 0.0203 | 1.67E-06 | 22.93784 |
|  |  | rs138897138 | 2 | 122731427 | A | G | 0.109 | 0.0216 | 0.914 | 4.50E-07 | 25.46511 |
|  |  | rs4664334 | 2 | 161368905 | T | C | 0.0541 | 0.0118 | 0.5003 | 4.55E-06 | 21.01989 |
|  |  | rs3967170 | 3 | 3773438 | A | G | 0.0789 | 0.017 | 0.1353 | 3.46E-06 | 21.54052 |
|  |  | rs12500563 | 4 | 7304426 | T | G | 0.3143 | 0.0687 | 0.9875 | 4.76E-06 | 20.93029 |
|  |  | rs9993874 | 4 | 55046724 | A | G | -0.0582 | 0.0125 | 0.4883 | 3.22E-06 | 21.67834 |
|  |  | rs141401004 | 5 | 22137911 | C | G | -0.0662 | 0.0129 | 0.4415 | 2.87E-07 | 26.3352 |
|  |  | rs551896428 | 6 | 141740384 | T | C | -0.3275 | 0.0705 | 0.9887 | 3.39E-06 | 21.57965 |
|  |  | rs200824954 | 7 | 29807677 | T | G | 0.1568 | 0.0335 | 0.0642 | 2.86E-06 | 21.90799 |
|  |  | rs75626299 | 9 | 29426599 | C | G | 0.1458 | 0.0316 | 0.9638 | 3.95E-06 | 21.2883 |
|  |  | rs9526031 | 13 | 45595067 | A | C | 0.064 | 0.012 | 0.4882 | 9.64E-08 | 28.44444 |
|  |  | rs8024256 | 15 | 26829521 | A | G | -0.0562 | 0.0121 | 0.3092 | 3.41E-06 | 21.57257 |
|  |  | rs404842 | 16 | 84304113 | A | G | -0.0599 | 0.0123 | 0.6433 | 1.12E-06 | 23.71611 |
|  |  | rs7250371 | 19 | 57861085 | T | C | 0.0686 | 0.0116 | 0.3648 | 3.34E-09 | 34.97295 |
|  |  | rs117191103 | 20 | 44464356 | T | C | 0.3553 | 0.0437 | 0.0208 | 4.28E-16 | 66.10397 |
|  |  | rs73128569 | 20 | 44606446 | A | T | -0.1788 | 0.0268 | 0.9406 | 2.53E-11 | 44.5108 |
|  |  | rs1883832 | 20 | 44746982 | T | C | -0.4519 | 0.0126 | 0.2575 | 1.07E-281 | 1286.304 |
|  |  | rs138739860 | 21 | 18103398 | T | G | 0.1632 | 0.0351 | 0.9648 | 3.33E-06 | 21.61853 |
|  |  | rs12152184 | 22 | 45002809 | A | G | 0.1085 | 0.0205 | 0.8849 | 1.21E-07 | 28.01249 |
| T-cell surface glycoprotein CD6 isoform levels | MS | rs143374617 | 1 | 4327524 | T | C | 0.1766 | 0.0329 | 0.0397 | 7.97E-08 | 28.81307 |
|  |  | rs841332 | 1 | 95171460 | A | G | -0.0721 | 0.0154 | 0.8286 | 2.84E-06 | 21.91942 |
|  |  | rs12074680 | 1 | 194299592 | A | G | -0.1855 | 0.0392 | 0.0301 | 2.22E-06 | 22.39318 |
|  |  | rs35086654 | 2 | 173467755 | T | C | -0.2416 | 0.0517 | 0.017 | 2.97E-06 | 21.838 |
|  |  | rs72969761 | 2 | 227938476 | A | T | -0.1796 | 0.0376 | 0.97 | 1.78E-06 | 22.81587 |
|  |  | rs74886615 | 4 | 83099601 | A | T | 0.0688 | 0.0129 | 0.6149 | 9.64E-08 | 28.44444 |
|  |  | rs574143589 | 7 | 115590077 | T | C | -0.3481 | 0.0733 | 0.9886 | 2.04E-06 | 22.55278 |
|  |  | rs9691968 | 7 | 153635380 | A | C | 0.0646 | 0.0134 | 0.2505 | 1.43E-06 | 23.24103 |
|  |  | rs115830154 | 8 | 79672562 | A | G | -0.0831 | 0.0169 | 0.8277 | 8.78E-07 | 24.17846 |
|  |  | rs60988380 | 9 | 33117954 | T | C | 0.0955 | 0.0193 | 0.1058 | 7.49E-07 | 24.48455 |
|  |  | rs7951625 | 11 | 60319883 | T | C | 0.0674 | 0.0134 | 0.2912 | 4.91E-07 | 25.2994 |
|  |  | rs4939464 | 11 | 60594256 | T | G | 0.1195 | 0.0175 | 0.1471 | 8.58E-12 | 46.62939 |
|  |  | rs4939488 | 11 | 60786474 | T | C | 0.5235 | 0.0112 | 0.5982 | 0 | 2184.728 |
|  |  | rs10792322 | 11 | 61761552 | T | C | -0.0662 | 0.0126 | 0.3002 | 1.49E-07 | 27.60418 |
|  |  | rs534268736 | 11 | 134282292 | T | C | 0.2909 | 0.0584 | 0.0166 | 6.32E-07 | 24.812 |
|  |  | rs597808 | 12 | 111973358 | A | G | 0.116 | 0.0134 | 0.4779 | 4.86E-18 | 74.93874 |
|  |  | rs8051133 | 16 | 86486037 | A | G | -0.1079 | 0.0236 | 0.0691 | 4.83E-06 | 20.90349 |
|  |  | rs35634144 | 22 | 37638221 | C | G | 0.1752 | 0.0376 | 0.9711 | 3.17E-06 | 21.71163 |
| C-X-C motif chemokine 10 levels | MS | rs112202639 | 1 | 44566391 | A | G | -0.1609 | 0.0336 | 0.9671 | 1.68E-06 | 22.93156 |
|  |  | rs1563171 | 2 | 3891773 | A | G | 0.0592 | 0.0127 | 0.7042 | 3.14E-06 | 21.72881 |
|  |  | rs12476448 | 2 | 14400474 | T | C | 0.0536 | 0.0115 | 0.4864 | 3.15E-06 | 21.72371 |
|  |  | rs12629593 | 3 | 54104178 | T | G | -0.0879 | 0.0182 | 0.1209 | 1.37E-06 | 23.32572 |
|  |  | rs72651343 | 4 | 76808216 | T | C | -0.6096 | 0.0619 | 0.9892 | 6.98E-23 | 96.9859 |
|  |  | rs115140093 | 4 | 76910673 | T | C | -0.5186 | 0.042 | 0.9732 | 5.02E-35 | 152.4637 |
|  |  | rs12646113 | 4 | 77175245 | T | C | 0.0977 | 0.0136 | 0.246 | 6.78E-13 | 51.60732 |
|  |  | rs72682331 | 4 | 112904535 | A | G | 0.1089 | 0.0209 | 0.0979 | 1.88E-07 | 27.14958 |
|  |  | rs2523495 | 6 | 31377978 | T | C | 0.1095 | 0.0196 | 0.1048 | 2.31E-08 | 31.2116 |
|  |  | rs71534595 | 6 | 32516855 | T | C | -0.2991 | 0.0606 | 0.9813 | 7.99E-07 | 24.36058 |
|  |  | rs12174864 | 6 | 88557407 | A | G | -0.0582 | 0.0121 | 0.6254 | 1.51E-06 | 23.1353 |
|  |  | rs12155428 | 7 | 3425301 | T | C | -0.1765 | 0.0375 | 0.0284 | 2.52E-06 | 22.15271 |
|  |  | rs2349689 | 8 | 62003730 | A | G | -0.0789 | 0.0164 | 0.2555 | 1.50E-06 | 23.14549 |
|  |  | rs143796249 | 9 | 4150061 | C | G | -0.2858 | 0.062 | 0.012 | 4.03E-06 | 21.24913 |
|  |  | rs10481754 | 9 | 84196490 | T | C | -0.0548 | 0.0119 | 0.4567 | 4.12E-06 | 21.20641 |
|  |  | rs4367871 | 10 | 1845059 | T | C | -0.0567 | 0.0119 | 0.5587 | 1.89E-06 | 22.70242 |
|  |  | rs12779465 | 10 | 78705575 | A | C | -0.0698 | 0.015 | 0.2969 | 3.27E-06 | 21.65351 |
|  |  | rs55732300 | 11 | 73731422 | A | G | 0.1175 | 0.025 | 0.929 | 2.60E-06 | 22.09 |
|  |  | rs741344 | 12 | 68597086 | A | G | -0.06 | 0.013 | 0.7036 | 3.92E-06 | 21.30178 |
|  |  | rs3184504 | 12 | 111884608 | T | C | 0.1138 | 0.0131 | 0.4756 | 3.72E-18 | 75.46437 |
|  |  | rs1077965 | 15 | 80065337 | A | G | -0.0546 | 0.0118 | 0.5648 | 3.71E-06 | 21.41023 |
|  |  | rs74956615 | 19 | 10427721 | A | T | -0.1358 | 0.0294 | 0.0495 | 3.86E-06 | 21.3356 |
|  |  | rs4804148 | 19 | 11270867 | T | C | -0.0629 | 0.013 | 0.7227 | 1.31E-06 | 23.41071 |
|  |  | rs150519056 | 22 | 50774821 | T | C | -0.1841 | 0.0376 | 0.9688 | 9.77E-07 | 23.97352 |
| Leukemia inhibitory factor receptor levels | MS | rs12754806 | 1 | 238821453 | A | G | 0.0563 | 0.0122 | 0.4817 | 3.94E-06 | 21.29596 |
|  |  | rs3963364 | 3 | 12385357 | A | C | -0.0951 | 0.0203 | 0.1784 | 2.80E-06 | 21.94669 |
|  |  | rs62258077 | 3 | 58363371 | T | C | -0.063 | 0.013 | 0.6603 | 1.26E-06 | 23.48521 |
|  |  | rs9313148 | 5 | 6378192 | C | G | -0.0824 | 0.0173 | 0.1414 | 1.91E-06 | 22.68622 |
|  |  | rs72730728 | 5 | 36424241 | A | T | 0.1414 | 0.0302 | 0.9403 | 2.84E-06 | 21.92224 |
|  |  | rs2289779 | 5 | 38527228 | T | G | 0.0788 | 0.0134 | 0.5419 | 4.09E-09 | 34.58142 |
|  |  | rs115938660 | 5 | 110784241 | T | C | -0.2181 | 0.0467 | 0.0215 | 3.01E-06 | 21.8111 |
|  |  | rs778016 | 6 | 68918580 | A | G | -0.0681 | 0.0145 | 0.2875 | 2.65E-06 | 22.0576 |
|  |  | rs73157388 | 7 | 79349730 | T | C | -0.1306 | 0.0285 | 0.9229 | 4.60E-06 | 20.9989 |
|  |  | rs117117000 | 8 | 119917062 | A | G | 0.1586 | 0.0333 | 0.961 | 1.91E-06 | 22.68391 |
|  |  | rs635634 | 9 | 136155000 | T | C | -0.2885 | 0.0153 | 0.1918 | 2.61E-79 | 355.5566 |
|  |  | rs41307428 | 9 | 136336804 | T | C | 0.2027 | 0.0299 | 0.067 | 1.21E-11 | 45.95842 |
|  |  | rs1690678 | 10 | 27930818 | A | C | 0.0904 | 0.0192 | 0.1109 | 2.50E-06 | 22.1684 |
|  |  | rs4937122 | 11 | 126228659 | T | G | 0.1262 | 0.0248 | 0.9129 | 3.61E-07 | 25.89497 |
|  |  | rs72837682 | 17 | 7063650 | T | C | -0.08 | 0.0172 | 0.7807 | 3.30E-06 | 21.63332 |
|  |  | rs141933232 | 17 | 36964094 | A | G | -0.2724 | 0.0546 | 0.0169 | 6.07E-07 | 24.89023 |
|  |  | rs62654645 | 21 | 34695981 | T | G | -0.0812 | 0.0172 | 0.5221 | 2.35E-06 | 22.28718 |
|  |  | rs147006060 | 21 | 43780807 | A | G | -0.2602 | 0.0566 | 0.0179 | 4.28E-06 | 21.134 |
|  |  | rs6002001 | 22 | 41054967 | A | G | 0.6489 | 0.1417 | 0.0043 | 4.66E-06 | 20.97084 |
| Neurturin levels | MS | rs4360502 | 1 | 214886642 | A | G | -0.0652 | 0.0141 | 0.3445 | 3.76E-06 | 21.38243 |
|  |  | rs62120726 | 2 | 15734798 | A | G | 0.0786 | 0.0172 | 0.8077 | 4.88E-06 | 20.88277 |
|  |  | rs908372 | 2 | 172434373 | C | G | -0.0622 | 0.0136 | 0.3324 | 4.80E-06 | 20.91717 |
|  |  | rs76777800 | 3 | 73067086 | T | C | 0.3387 | 0.0738 | 0.0176 | 4.44E-06 | 21.06288 |
|  |  | rs143131035 | 4 | 43313220 | T | C | -0.4361 | 0.0867 | 0.0118 | 4.91E-07 | 25.30078 |
|  |  | rs7712604 | 5 | 21508959 | C | G | -0.0879 | 0.0176 | 0.6738 | 5.90E-07 | 24.94321 |
|  |  | rs29630 | 5 | 171026588 | T | C | -0.074 | 0.0159 | 0.2493 | 3.25E-06 | 21.66054 |
|  |  | rs192427410 | 8 | 43185781 | T | G | 0.3743 | 0.0786 | 0.0252 | 1.92E-06 | 22.67747 |
|  |  | rs77216559 | 11 | 4475910 | T | C | 0.1667 | 0.034 | 0.958 | 9.44E-07 | 24.03883 |
|  |  | rs182274799 | 11 | 10358788 | A | G | 0.1565 | 0.031 | 0.0646 | 4.46E-07 | 25.48621 |
|  |  | rs4753678 | 11 | 95201232 | A | G | 0.087 | 0.0178 | 0.5434 | 1.02E-06 | 23.88903 |
|  |  | rs8033310 | 15 | 24046105 | T | C | 0.1332 | 0.0286 | 0.9133 | 3.20E-06 | 21.69084 |
|  |  | rs28759830 | 16 | 21786236 | T | C | -0.1778 | 0.037 | 0.9507 | 1.54E-06 | 23.09192 |
|  |  | rs7202385 | 16 | 26080883 | A | T | 0.0746 | 0.0157 | 0.6725 | 2.02E-06 | 22.57763 |
|  |  | rs10412462 | 19 | 14691684 | A | C | -0.1731 | 0.0366 | 0.0363 | 2.25E-06 | 22.36825 |
|  |  | rs113580784 | 20 | 55469957 | A | G | -0.2288 | 0.0495 | 0.0208 | 3.80E-06 | 21.36494 |
| Fibroblast growth factor 21 levels | PD | rs145127946 | 1 | 203746507 | A | G | -0.1747 | 0.036 | 0.037 | 1.22E-06 | 23.54945 |
|  |  | rs10495032 | 1 | 216837226 | T | G | -0.1557 | 0.0318 | 0.0383 | 9.77E-07 | 23.97303 |
|  |  | rs1260326 | 2 | 27730940 | T | C | 0.1323 | 0.0119 | 0.3977 | 1.03E-28 | 123.6021 |
|  |  | rs7610704 | 3 | 11689679 | T | C | -0.0568 | 0.012 | 0.4644 | 2.21E-06 | 22.40444 |
|  |  | rs4700382 | 5 | 55882435 | T | C | -0.063 | 0.0136 | 0.2698 | 3.62E-06 | 21.45869 |
|  |  | rs60277384 | 5 | 84356011 | T | C | -0.0881 | 0.019 | 0.8922 | 3.54E-06 | 21.5003 |
|  |  | rs13229619 | 7 | 73030175 | A | G | -0.1609 | 0.0176 | 0.1297 | 6.13E-20 | 83.577 |
|  |  | rs7012637 | 8 | 9173209 | A | G | -0.0605 | 0.012 | 0.4812 | 4.61E-07 | 25.4184 |
|  |  | rs188758663 | 10 | 127847758 | A | G | -0.3801 | 0.0749 | 0.0104 | 3.88E-07 | 25.75325 |
|  |  | rs12290350 | 11 | 76480255 | T | C | -0.0689 | 0.0145 | 0.2168 | 2.02E-06 | 22.57888 |
|  |  | rs2429473 | 12 | 47198899 | A | C | -0.0812 | 0.0147 | 0.795 | 3.32E-08 | 30.51247 |
|  |  | rs72965996 | 18 | 72826607 | A | G | -0.2215 | 0.0444 | 0.031 | 6.08E-07 | 24.88751 |
|  |  | rs838131 | 19 | 49260677 | A | C | 0.1627 | 0.013 | 0.501 | 6.15E-36 | 156.6349 |
|  |  | rs142603673 | 21 | 33860969 | T | C | -0.2352 | 0.0455 | 0.0232 | 2.35E-07 | 26.72095 |
| Interleukin-12 subunit beta levels | PD | rs6750065 | 2 | 64889908 | T | C | 0.0628 | 0.0134 | 0.7333 | 2.78E-06 | 21.96391 |
|  |  | rs149993822 | 2 | 112046960 | T | C | 0.0984 | 0.0194 | 0.1662 | 3.93E-07 | 25.72686 |
|  |  | rs11130215 | 3 | 5026008 | A | G | -0.1009 | 0.0151 | 0.8154 | 2.36E-11 | 44.65072 |
|  |  | rs148549227 | 3 | 171348710 | T | C | 0.1088 | 0.0228 | 0.0889 | 1.82E-06 | 22.77131 |
|  |  | rs9815073 | 3 | 188115682 | A | C | 0.2861 | 0.013 | 0.3539 | 2.43E-107 | 484.3385 |
|  |  | rs5014495 | 4 | 190527932 | T | C | 0.207 | 0.0442 | 0.0363 | 2.82E-06 | 21.9329 |
|  |  | rs144850787 | 5 | 158693364 | T | G | 0.3161 | 0.056 | 0.9869 | 1.66E-08 | 31.86199 |
|  |  | rs4921487 | 5 | 158796325 | A | G | -0.4972 | 0.0122 | 0.3028 | 0 | 1660.897 |
|  |  | rs17056751 | 5 | 158852200 | A | G | 0.1694 | 0.0325 | 0.9592 | 1.87E-07 | 27.16815 |
|  |  | rs17722688 | 5 | 158907737 | T | C | -0.0857 | 0.0131 | 0.7246 | 6.07E-11 | 42.79756 |
|  |  | rs3130510 | 6 | 31154493 | A | G | 0.1155 | 0.014 | 0.2275 | 1.58E-16 | 68.0625 |
|  |  | rs6968201 | 7 | 6408796 | T | G | -0.0622 | 0.0119 | 0.5425 | 1.72E-07 | 27.32039 |
|  |  | rs117888068 | 8 | 4597842 | T | C | 0.219 | 0.0472 | 0.9787 | 3.49E-06 | 21.52803 |
|  |  | rs780679 | 10 | 73114509 | T | C | -0.0695 | 0.0139 | 0.2389 | 5.73E-07 | 25 |
|  |  | rs12766137 | 10 | 94525783 | C | G | 0.0709 | 0.0149 | 0.8109 | 1.95E-06 | 22.64227 |
|  |  | rs11039216 | 11 | 47406592 | T | C | 0.0588 | 0.0116 | 0.5207 | 4.00E-07 | 25.69441 |
|  |  | rs539598 | 11 | 96026074 | T | C | -0.0672 | 0.0129 | 0.2822 | 1.90E-07 | 27.13683 |
|  |  | rs72993671 | 11 | 115767583 | A | G | -0.204 | 0.0435 | 0.0216 | 2.74E-06 | 21.99287 |
|  |  | rs1398590 | 12 | 17314071 | A | G | -0.197 | 0.0363 | 0.0298 | 5.73E-08 | 29.4523 |
|  |  | rs705705 | 12 | 56435504 | C | G | 0.0785 | 0.0133 | 0.323 | 3.59E-09 | 34.83662 |
|  |  | rs3184504 | 12 | 111884608 | T | C | 0.1332 | 0.0131 | 0.4766 | 2.76E-24 | 103.387 |
|  |  | rs516978 | 12 | 121197553 | C | G | 0.0721 | 0.0122 | 0.3473 | 3.42E-09 | 34.92616 |
|  |  | rs76428106 | 13 | 28604007 | T | C | -0.3406 | 0.0535 | 0.9832 | 1.94E-10 | 40.53048 |
|  |  | rs1950897 | 14 | 68760141 | T | C | 0.0798 | 0.0128 | 0.7151 | 4.54E-10 | 38.86743 |
|  |  | rs12588969 | 14 | 103230758 | C | G | -0.1297 | 0.0127 | 0.6875 | 1.74E-24 | 104.2972 |
|  |  | rs8026767 | 15 | 80260554 | A | G | -0.0743 | 0.0132 | 0.2595 | 1.81E-08 | 31.68325 |
|  |  | rs9965402 | 18 | 26624891 | A | G | 0.0918 | 0.0201 | 0.9081 | 4.94E-06 | 20.85899 |
|  |  | rs73416208 | 22 | 37881211 | A | G | -0.1477 | 0.0246 | 0.0654 | 1.92E-09 | 36.0488 |
| Interleukin-17A levels | PD | rs33998516 | 1 | 96622380 | A | C | -0.0802 | 0.0164 | 0.7781 | 1.01E-06 | 23.91449 |
|  |  | rs75788494 | 2 | 13468626 | T | C | -0.2432 | 0.0525 | 0.021 | 3.61E-06 | 21.45895 |
|  |  | rs115193412 | 3 | 63484616 | T | C | 0.1688 | 0.0349 | 0.0418 | 1.32E-06 | 23.39344 |
|  |  | rs17448036 | 3 | 189579511 | A | G | -0.0585 | 0.0128 | 0.5609 | 4.87E-06 | 20.88776 |
|  |  | rs41270871 | 6 | 32628224 | A | T | 0.0923 | 0.0189 | 0.221 | 1.04E-06 | 23.84953 |
|  |  | rs28852438 | 7 | 6380998 | A | G | -0.068 | 0.0148 | 0.2897 | 4.34E-06 | 21.1103 |
|  |  | rs1462017 | 8 | 70106392 | C | G | -0.0893 | 0.017 | 0.1776 | 1.50E-07 | 27.59339 |
|  |  | rs77529534 | 9 | 27215497 | T | C | -0.1279 | 0.0272 | 0.9323 | 2.57E-06 | 22.11074 |
|  |  | rs79982350 | 9 | 31560030 | C | G | -0.1467 | 0.0295 | 0.0555 | 6.60E-07 | 24.72955 |
|  |  | rs4877075 | 9 | 91976386 | A | G | 0.0908 | 0.0192 | 0.1411 | 2.25E-06 | 22.36502 |
|  |  | rs34557923 | 10 | 67955765 | T | G | 0.2446 | 0.0517 | 0.9819 | 2.23E-06 | 22.3837 |
|  |  | rs77595989 | 12 | 118130693 | T | G | 0.1696 | 0.0371 | 0.9648 | 4.84E-06 | 20.89796 |
|  |  | rs12599620 | 16 | 8011846 | T | C | -0.0726 | 0.0153 | 0.7391 | 2.08E-06 | 22.51596 |
|  |  | rs2429911 | 17 | 2272057 | T | C | 0.2309 | 0.0486 | 0.0251 | 2.02E-06 | 22.57227 |
|  |  | rs2693361 | 17 | 43658721 | T | G | 0.0884 | 0.0185 | 0.517 | 1.77E-06 | 22.8329 |
| Neurturin levels | PD | rs4360502 | 1 | 214886642 | A | G | -0.0652 | 0.0141 | 0.3445 | 3.76E-06 | 21.38243 |
|  |  | rs62120726 | 2 | 15734798 | A | G | 0.0786 | 0.0172 | 0.8077 | 4.88E-06 | 20.88277 |
|  |  | rs908372 | 2 | 172434373 | C | G | -0.0622 | 0.0136 | 0.3324 | 4.80E-06 | 20.91717 |
|  |  | rs76777800 | 3 | 73067086 | T | C | 0.3387 | 0.0738 | 0.0176 | 4.44E-06 | 21.06288 |
|  |  | rs143131035 | 4 | 43313220 | T | C | -0.4361 | 0.0867 | 0.0118 | 4.91E-07 | 25.30078 |
|  |  | rs7712604 | 5 | 21508959 | C | G | -0.0879 | 0.0176 | 0.6738 | 5.90E-07 | 24.94321 |
|  |  | rs29630 | 5 | 171026588 | T | C | -0.074 | 0.0159 | 0.2493 | 3.25E-06 | 21.66054 |
|  |  | rs192427410 | 8 | 43185781 | T | G | 0.3743 | 0.0786 | 0.0252 | 1.92E-06 | 22.67747 |
|  |  | rs77216559 | 11 | 4475910 | T | C | 0.1667 | 0.034 | 0.958 | 9.44E-07 | 24.03883 |
|  |  | rs182274799 | 11 | 10358788 | A | G | 0.1565 | 0.031 | 0.0646 | 4.46E-07 | 25.48621 |
|  |  | rs4753678 | 11 | 95201232 | A | G | 0.087 | 0.0178 | 0.5434 | 1.02E-06 | 23.88903 |
|  |  | rs8033310 | 15 | 24046105 | T | C | 0.1332 | 0.0286 | 0.9133 | 3.20E-06 | 21.69084 |
|  |  | rs28759830 | 16 | 21786236 | T | C | -0.1778 | 0.037 | 0.9507 | 1.54E-06 | 23.09192 |
|  |  | rs7202385 | 16 | 26080883 | A | T | 0.0746 | 0.0157 | 0.6725 | 2.02E-06 | 22.57763 |
|  |  | rs10412462 | 19 | 14691684 | A | C | -0.1731 | 0.0366 | 0.0363 | 2.25E-06 | 22.36825 |
|  |  | rs113580784 | 20 | 55469957 | A | G | -0.2288 | 0.0495 | 0.0208 | 3.80E-06 | 21.36494 |
| Transforming growth factor-alpha levels | PD | rs78912149 | 1 | 166175871 | C | G | -0.2692 | 0.056 | 0.9835 | 1.53E-06 | 23.10862 |
|  |  | rs72912115 | 2 | 70774295 | A | T | 0.137 | 0.0184 | 0.114 | 9.65E-14 | 55.43774 |
|  |  | rs34007466 | 4 | 16165036 | A | G | -0.2684 | 0.05 | 0.0153 | 7.96E-08 | 28.81542 |
|  |  | rs11242125 | 5 | 132025905 | A | C | -0.0761 | 0.0166 | 0.7246 | 4.55E-06 | 21.01615 |
|  |  | rs9262670 | 6 | 31034990 | T | C | 0.0717 | 0.0146 | 0.2343 | 9.06E-07 | 24.11752 |
|  |  | rs1963491 | 8 | 2654153 | C | G | 0.1081 | 0.023 | 0.905 | 2.60E-06 | 22.09 |
|  |  | rs34630685 | 8 | 13724808 | C | G | 0.1678 | 0.0361 | 0.9681 | 3.35E-06 | 21.60576 |
|  |  | rs74130123 | 10 | 30042437 | T | C | -0.2601 | 0.0508 | 0.9817 | 3.05E-07 | 26.21521 |
|  |  | rs4934441 | 10 | 90836825 | A | G | -0.095 | 0.0198 | 0.1322 | 1.60E-06 | 23.02061 |
|  |  | rs12270510 | 11 | 119506018 | T | C | 0.0959 | 0.019 | 0.1107 | 4.48E-07 | 25.47593 |
|  |  | rs653178 | 12 | 112007756 | T | C | -0.0708 | 0.0131 | 0.5228 | 6.50E-08 | 29.20949 |
|  |  | rs3859189 | 17 | 38137033 | A | G | 0.0655 | 0.0116 | 0.5369 | 1.64E-08 | 31.88355 |
|  |  | rs111613293 | 17 | 52902972 | A | T | -0.0581 | 0.0122 | 0.4071 | 1.91E-06 | 22.67945 |
|  |  | rs78464136 | 19 | 15885492 | A | T | 0.143 | 0.0307 | 0.9552 | 3.19E-06 | 21.69678 |
|  |  | rs149146289 | 19 | 58857883 | A | G | 0.2856 | 0.0569 | 0.9866 | 5.19E-07 | 25.1937 |
| Tumor necrosis factor receptor superfamily member 9 levels | PD | rs1776354 | 1 | 7972201 | A | G | -0.1301 | 0.013 | 0.5203 | 1.41E-23 | 100.1539 |
|  |  | rs10178845 | 2 | 8443803 | A | G | -0.0682 | 0.0144 | 0.3043 | 2.18E-06 | 22.43075 |
|  |  | rs2579649 | 2 | 120226781 | A | C | -0.0968 | 0.0199 | 0.1322 | 1.15E-06 | 23.66162 |
|  |  | rs150949259 | 3 | 177868759 | A | G | 0.375 | 0.0809 | 0.0104 | 3.56E-06 | 21.48649 |
|  |  | rs140214797 | 4 | 28155085 | T | G | 0.2317 | 0.0504 | 0.9796 | 4.28E-06 | 21.13445 |
|  |  | rs72964492 | 4 | 153651229 | A | C | 0.3111 | 0.065 | 0.9869 | 1.70E-06 | 22.90727 |
|  |  | rs7737635 | 5 | 124329728 | A | G | 0.0924 | 0.0201 | 0.1947 | 4.29E-06 | 21.13255 |
|  |  | rs75246422 | 5 | 174179760 | T | C | -0.0982 | 0.0203 | 0.1231 | 1.32E-06 | 23.40081 |
|  |  | rs4976685 | 5 | 176766177 | A | G | -0.0693 | 0.0139 | 0.6695 | 6.18E-07 | 24.85632 |
|  |  | rs2854008 | 6 | 31312538 | A | G | 0.0926 | 0.0149 | 0.2629 | 5.14E-10 | 38.62331 |
|  |  | rs146380867 | 8 | 50188685 | A | G | 0.3742 | 0.0814 | 0.0105 | 4.29E-06 | 21.13288 |
|  |  | rs10101219 | 8 | 99918668 | C | G | -0.4015 | 0.0847 | 0.9895 | 2.13E-06 | 22.47006 |
|  |  | rs1846520 | 8 | 118333064 | A | G | -0.1626 | 0.0313 | 0.9533 | 2.05E-07 | 26.98686 |
|  |  | rs7858310 | 9 | 87717237 | A | G | 0.0707 | 0.0152 | 0.7165 | 3.30E-06 | 21.63474 |
|  |  | rs11202989 | 10 | 90880926 | T | C | -0.0995 | 0.0212 | 0.1219 | 2.69E-06 | 22.02797 |
|  |  | rs72850206 | 11 | 2632174 | A | T | -0.1438 | 0.0281 | 0.0736 | 3.10E-07 | 26.18817 |
|  |  | rs9669611 | 12 | 6503786 | T | C | 0.0703 | 0.015 | 0.2853 | 2.78E-06 | 21.96484 |
|  |  | rs12582121 | 12 | 67226362 | T | C | 0.0645 | 0.0135 | 0.6481 | 1.77E-06 | 22.82716 |
|  |  | rs3184504 | 12 | 111884608 | T | C | 0.0681 | 0.013 | 0.476 | 1.62E-07 | 27.44148 |
|  |  | rs10744409 | 12 | 129783438 | A | G | 0.064 | 0.0131 | 0.4447 | 1.03E-06 | 23.86807 |
|  |  | rs76428106 | 13 | 28604007 | T | C | -0.2714 | 0.0593 | 0.9828 | 4.72E-06 | 20.94644 |
|  |  | rs143274881 | 13 | 58754621 | A | T | -0.3325 | 0.0719 | 0.9767 | 3.76E-06 | 21.3858 |
|  |  | rs12874404 | 13 | 108993494 | A | G | -0.1402 | 0.0296 | 0.9406 | 2.17E-06 | 22.4343 |
|  |  | rs8005108 | 14 | 20709993 | C | G | 0.0889 | 0.0182 | 0.2828 | 1.04E-06 | 23.85947 |
|  |  | rs57700441 | 14 | 63713358 | C | G | -0.1155 | 0.0228 | 0.901 | 4.07E-07 | 25.66222 |
|  |  | rs118083884 | 17 | 16522922 | A | G | 0.3284 | 0.0668 | 0.019 | 8.83E-07 | 24.1687 |
|  |  | rs1645331 | 19 | 49126012 | C | G | 0.097 | 0.0206 | 0.8419 | 2.49E-06 | 22.17221 |
|  |  | rs192538756 | 20 | 2629394 | A | T | 0.1189 | 0.0242 | 0.8742 | 8.96E-07 | 24.13976 |

**Table S4.** Druggability of proteins potentially causally associated with Neurodegenerative Diseases.

| **Protein** | **Protein full name** | **Drug or component name** | **Drug groups** | **Indication** |
| --- | --- | --- | --- | --- |
| CXCL1 | C-X-C motif chemokine 1 | [WWL123](https://dgidb.org/drugs/iuphar.ligand:9480) | Not Approved | CXCL1 inhibitor |
|  |  | WWL70 | Not Approved | CXCL1 inhibitor |
|  |  | KT-109 | Not Approved | CXCL1 inhibitor |
|  |  | ABX-1431 | Not Approved | CXCL1 inhibitor |
| FLT3 | Fms-related tyrosine  kinase 3 ligand | PF-04691502 | Not Approved |  |
|  |  | APG-2575 | Not Approved |  |
|  |  | QUIZARTINIB | Approved | Antineoplastic agent |
|  |  | SELINEXOR | Approved |  |
|  |  | GW843682X | Not Approved |  |
|  |  | RECOMBINANT TRANSFORMING  GROWTH FACTOR | Not Approved |  |
|  |  | CHEMBL:CHEMBL536151 | Not Approved |  |
|  |  | SORAFENIB | Approved | Antineoplastic agent |
|  |  | VS-5584 | Not Approved |  |
|  |  | TYRPHOSTIN AG 1296 | Not Approved |  |
|  |  | TOFACITINIB | Approved |  |
|  |  | LUXEPTINIB | Not Approved |  |
|  |  | CRENOLANIB | Not Approved |  |
|  |  | PONATINIB | Approved | Antineoplastic agent |
|  |  | IMATINIB | Approved | Antineoplastic agent |
|  |  | ONO-7475 | Not Approved |  |
|  |  | EVEROLIMUS | Approved | Immunosuppressant |
|  |  | OMACETAXINE  MEPESUCCINATE | Approved | Antineoplastic agent |
|  |  | MELK INHIBITOR  OTS167 | Not Approved |  |
|  |  | PEXIDARTINIB | Approved |  |
|  |  | FLT3 INHIBITOR  BMF-500 | Not Approved |  |
|  |  | DUBERMATINIB | Not Approved |  |
|  |  | KENPAULLONE | Not Approved |  |
|  |  | MIDOSTAURIN | Approved | Antineoplastic agent |
|  |  | MOMELOTINIB | Approved |  |
|  |  | TANDUTINIB | Not Approved | Antineoplastic agent |
|  |  | ALISERTIB | Not Approved | Antineoplastic agent |
|  |  | FF-10101 | Not Approved |  |
|  |  | PF-562271 | Not Approved |  |
|  |  | CLOFARABINE | Approved | Antineoplastic agent |
|  |  | IDARUBICIN | Approved | Antineoplastic agents |
|  |  | TIRBANIBULIN | Approved | Antineoplastic agent |
|  |  | AMUVATINIB | Not Approved | Antineoplastic agent |
|  |  | TUSPETINIB | Not Approved |  |
|  |  | ENASIDENIB | Approved |  |
|  |  | FLT3/CDK4/6  INHIBITOR FLX925 | Not Approved |  |
|  |  | IMC-EB10 | Not Approved |  |
|  |  | DANUSERTIB | Not Approved |  |
|  |  | LAS38096 | Not Approved |  |
|  |  | AZACITIDINE | Approved | Antineoplastic agent |
|  |  | GTP-14564 | Not Approved |  |
|  |  | OLVEREMBATINIB | Not Approved |  |
|  |  | ALTIRATINIB | Not Approved |  |
|  |  | LESTAURTINIB | Not Approved | Antineoplastic agent |
|  |  | IBRUTINIB | Approved | Antineoplastic agent |
|  |  | TALAZOPARIB | Approved |  |
|  |  | AKN-028 | Not Approved |  |
|  |  | VENETOCLAX | Approved |  |
|  |  | 4SC-203 | Not Approved |  |
|  |  | GSK690693 | Not Approved |  |
|  |  | ENMD-981693 | Not Approved |  |
|  |  | PACRITINIB | Approved | Antineoplastic agent |
|  |  | ABIVERTINIB | Not Approved |  |
|  |  | CHEMBL:CHEMBL1997335 | Not Approved |  |
|  |  | ANTISERUM | Not Approved |  |
|  |  | AS-602868 | Not Approved |  |
|  |  | ENTRECTINIB | Approved |  |
|  |  | AVAPRITINIB | Approved |  |
|  |  | NVP-TAE684 | Not Approved |  |
|  |  | PEXIDARTINIB  HYDROCHLORIDE | Approved |  |
|  |  | ANTI-FLT3/CD3 BISPECIFIC  ANTIBODY CLN-049 | Not Approved |  |
|  |  | BRIGATINIB | Approved |  |
|  |  | FLT3 TYROSINE KINASE  INHIBITOR TTT-3002 | Not Approved |  |
|  |  | ITACNOSERTIB | Not Approved |  |
|  |  | AST-487 | Not Approved |  |
|  |  | PD98059 | Not Approved |  |
|  |  | DENFIVONTINIB  HYDROCHLORIDE | Not Approved |  |
|  |  | TRETINOIN | Approved | For treatment of acne |
|  |  | HSP90 INHIBITOR LAM-003 | Not Approved |  |
|  |  | NEMTABRUTINIB | Not Approved |  |
|  |  | SUNITINIB | Approved | Antineoplastic agent |
|  |  | TYROSINE KINASE INHIBITOR | Not Approved |  |
|  |  | E6201 | Not Approved |  |
|  |  | FLT3 INHIBITOR FF-10101  SUCCINATE | Not Approved |  |
|  |  | CYTARABINE | Approved | Antineoplastic agent |
|  |  | K 252A | Not Approved |  |
|  |  | GILTERITINIB | Approved |  |
|  |  | AMG553 | Not Approved |  |
|  |  | CT-1578 | Not Approved |  |
|  |  | TAMATINIB | Not Approved |  |
|  |  | C-1311 | Not Approved | Antineoplastic agent |
|  |  | NILOTINIB | Approved | Antineoplastic agent |
|  |  | PHI-101 | Not Approved |  |
|  |  | GILTERITINIB  FUMARATE | Approved |  |
|  |  | METHOTREXATE | Approved | DMARD |
|  |  | CDX-301 | Not Approved |  |
|  |  | CABOZANTINIB  S-MALATE | Approved | Antineoplastic agent |
|  |  | BORTEZOMIB | Approved | Antineoplastic agent |
|  |  | ENTOSPLETINIB | Not Approved |  |
|  |  | ARSENIC TRIOXIDE | Approved | Antineoplastic agent |
|  |  | TYRPHOSTIN AG 1295 | Not Approved |  |
|  |  | XL999 | Not Approved | Antineoplastic agent |
|  |  | RECOMBINANT  TRANSFORMING GROWTH  FACTOR-BETA 1 | Not Approved |  |
|  |  | ENMD-2076 | Not Approved | Antineoplastic agent |
|  |  | FLYSYN | Not Approved |  |
|  |  | SHP2 INHIBITOR ERAS-601 | Not Approved |  |
|  |  | DECITABINE | Approved | Antineoplastic agent |
|  |  | CYC-116 | Not Approved |  |
|  |  | FEDRATINIB | Approved | Antineoplastic agent |
|  |  | FAMITINIB | Not Approved |  |
|  |  | CENISERTIB | Not Approved | Antineoplastic agent |
|  |  | AGS62P1 | Not Approved |  |
|  |  | 4SC-202 | Not Approved | Antineoplastic agent |
|  |  | FLT-3 INHIBITOR | Not Approved |  |
|  |  | MRX-2843 | Not Approved |  |
|  |  | FLT3/ABL/AURORA KINASE  INHIBITOR KW-2449 | Not Approved |  |
|  |  | FIMEPINOSTAT | Not Approved |  |
|  |  | SONIDEGIB | Approved | Antineoplastic agent |
|  |  | CM-082 | Not Approved |  |
|  |  | ZOTIRACICLIB | Not Approved |  |
|  |  | AKT INHIBITOR MK2206 | Not Approved |  |
|  |  | FN-1501 | Not Approved |  |
|  |  | AT-9283 | Not Approved |  |
|  |  | AZD1480 | Not Approved |  |
|  |  | GELDANAMYCIN | Not Approved |  |
|  |  | SU-014813 | Not Approved |  |
|  |  | BAY 61-3606 | Not Approved |  |
|  |  | TAZEMETOSTAT | Approved |  |
|  |  | BARASERTIB-HQPA | Not Approved |  |
|  |  | REVUMENIB | Not Approved |  |
|  |  | GO-6976 | Not Approved |  |
|  |  | ANTHRACYCLINE  ANTINEOPLASTIC  ANTIBIOTIC | Not Approved |  |
|  |  | REBASTINIB | Not Approved |  |
|  |  | SP-600125 | Not Approved |  |
|  |  | HESPERADIN | Not Approved |  |
|  |  | PATIDEGIB | Not Approved |  |
|  |  | GLASDEGIB | Approved |  |
|  |  | OLAPARIB | Approved |  |
|  |  | ILORASERTIB | Not Approved |  |
|  |  | SGI-1776 | Not Approved | Antineoplastic agent |
|  |  | AGL 2043 | Not Approved |  |
|  |  | RUXOLITINIB | Approved | Antiinflammatory agent, antineoplastic agent |
|  |  | IPATASERTIB | Not Approved | Antineoplastic agent |
|  |  | HERBIMYCIN A | Not Approved |  |
|  |  | GMI-1359 | Not Approved |  |
|  |  | MIRDAMETINIB | Not Approved |  |
|  |  | PACRITINIB CITRATE | Approved |  |
|  |  | CEP-2563 | Not Approved |  |
|  |  | DAUNORUBICIN  LIPOSOMAL | Approved | Antineoplastic agent |
|  |  | AGI-5198 | Not Approved |  |
|  |  | P505-15 | Not Approved | Antiinflammatory agent |
|  |  | FEDRATINIB  HYDROCHLORIDE | Approved |  |
|  |  | XL-999 | Not Approved |  |
|  |  | SITRAVATINIB | Not Approved |  |
|  |  | RG-1530 | Not Approved |  |
|  |  | RUSERONTINIB | Not Approved |  |
|  |  | PD-0166285 | Not Approved |  |
|  |  | KW-2449 | Not Approved |  |
|  |  | NINGETINIB | Not Approved |  |
|  |  | TRAMETINIB DIMETHYL  SULFOXIDE | Approved | Antineoplastic agent |
|  |  | DOXORUBICIN  HYDROCHLORIDE | Approved | Antineoplastic agent |
|  |  | AG 1295 | Not Approved |  |
|  |  | TOZASERTIB | Not Approved | Antineoplastic agent |
|  |  | DOVITINIB | Not Approved | Antineoplastic agent |
|  |  | SEMAXANIB | Not Approved |  |
|  |  | PALBOCICLIB | Approved |  |
|  |  | FORETINIB | Not Approved | Antineoplastic agent |
|  |  | CHEMBL:CHEMBL535331 | Not Approved |  |
|  |  | LINIFANIB | Not Approved | Antineoplastic agent |
|  |  | ASTX029 | Not Approved |  |
|  |  | GSK-269962A | Not Approved |  |
|  |  | MITOXANTRONE | Approved | Antineoplastic agent |
| IL18 | Interleukin-18 | TADEKINIG ALFA | Not Approved |  |
|  |  | GSK-1070806 | Not Approved |  |
|  |  | DEXTRAN SULFATE  SODIUM | Not Approved |  |
|  |  | THERAPEUTIC  CORTICOSTEROID | Not Approved |  |
|  |  | CAMOTESKIMAB | Not Approved |  |
|  |  | INTERFERON ALFA-2B | Approved | Antineoplastic Agents;  Immunomodulatory Agents, for treatment of hepatitis B and C, for treatment of hepatitis, for treatment of hepatitis C |
|  |  | IBOCTADEKIN | Not Approved |  |
|  |  | 4-PHENYLENEDIAMINE | Not Approved |  |
|  |  | RECOMBINANT  INTERFERON | Not Approved |  |
|  |  | PEGINTERFERON  ALFA-2B | Approved |  |
|  |  | COLCHICINE | Approved | For treatment of gout |
|  |  | ANTIVIRAL AGENT | Not Approved |  |
|  |  | PEGINTERFERON  ALFA-2A | Approved | For treatment of hepatitis B and C, Antineoplastic Agents;  Immunomodulatory Agents |
|  |  | CAMOTESKIMAB | Not Approved |  |
|  |  | RIBAVIRIN | Approved |  |
|  |  | ANHYDROUS  TACROLIMUS | Approved | Immunosuppressant |
|  |  | THYROXINE | Not Approved |  |
|  |  | MYCOPHENOLATE | Approved | Immunosuppressant |
|  |  | SYNTHETIC HUMAN  PAPILLOMAVIRUS 16  E6 PEPTIDE | Not Approved |  |
| LIFR | Leukemia inhibitory  factor receptor | EMFILERMIN | Not Approved |  |
| MCP1  /CCL2 | Monocyte  chemoattractant  protein-1 | RISPERIDONE | Approved | Antipsychotic agent, Antipsychotic Agents |
|  |  | CARLUMAB | Not Approved |  |
|  |  | RS-504393 | Not Approved |  |
|  |  | BINDARIT | Not Approved |  |
|  |  | EMAPTICAP PEGOL | Not Approved | For treatment of  kidney disease |
|  |  | RECOMBINANT  MONOCYTE  CHEMOATTRACTANT  PROTEIN-1 | Not Approved |  |
| TNFSF12 | Tumor necrosis factor ligand superfamily member 12 | RO-5458640 | Not Approved |  |
|  |  | BIIB-023 | Not Approved |  |
| CD40L | CD40L receptor | ALDESLEUKIN | Approved | Antineoplastic agent |
|  |  | ROSIGLITAZONE | Approved | For treatment of  Alzheimer's disease, antidiabetic |
|  |  | ANTI-CD40 MONOCLONAL  ANTIBODY CHI LOB 7/4 | Not Approved |  |
|  |  | MUROMONAB-CD3 | Approved | Immunosuppressive Agents |
|  |  | TORALIZUMAB | Not Approved |  |
|  |  | CYCLOSPORINE | Approved | Immunosuppressant, opthalmological agent |
|  |  | BULEVIRTIDE | Not Approved |  |
|  |  | AUTOLOGOUS PROSTATE CANCER ANTIGEN- EXPRESSING  DENDRITIC CELL  VACCINE BPX-101 | Not Approved | Antineoplastic agent |
|  |  | URSODIOL | Approved | For prevention of recurrence  of colorectal polyps |
|  |  | BISPECIFIC ANTIBODY | Not Approved |  |
|  |  | ANTINEOPLASTIC  VACCINE | Not Approved |  |
|  |  | AT-1501 | Not Approved |  |
|  |  | LUCATUMUMAB | Not Approved | Antineoplastic agent |
|  |  | DAPIROLIZUMAB PEGOL | Not Approved |  |
|  |  | DAZODALIBEP | Not Approved |  |
|  |  | PG-102 | Not Approved |  |
|  |  | PROPRANOLOL  HYDROCHLORIDE | Approved | For treatment of  cancer cachexia |
|  |  | ADALIMUMAB-ADBM | Approved | DMARD, antiinflammatory agent |
|  |  | DAZODALIBEP | Not Approved |  |
|  |  | DEXPROPRANOLOL | Not Approved |  |
|  |  | IRBESARTAN | Approved | Antihypertensive agent |
|  |  | IMATINIB | Approved | Antineoplastic agent |
|  |  | FENOFIBRATE  MICRONIZED | Approved | Anticholesterolaemic agent, antidyslipidaemic agent |
|  |  | ALPROSTADIL | Approved | For treatment of sexual dysfunction in women, for treatment of erectile dysfunction |
|  |  | RUPLIZUMAB | Not Approved |  |
|  |  | RAVAGALIMAB | Not Approved |  |
|  |  | ANTI-CD40/ ANTI-MESOTHELIN  BISPECIFIC ANTIBODY  ABBV-428 | Not Approved |  |
|  |  | ATORVASTATIN  CALCIUM TRIHYDRATE | Approved | Anticholesterolaemic agent, antihypecholesterolemic agent |
|  |  | SELICRELUMAB | Not Approved |  |
| IL12β | Interleukin-12  subunit beta | VIRULIZIN | Not Approved | Antineoplastic agent |
|  |  | ISOPROTERENOL | Approved | Bronchodilator Agents;  Cardiotonic Agents |
|  |  | MIRIKIZUMAB | Approved |  |
|  |  | USTEKINUMAB | Approved | DMARD, antiinflammatory agent, Antipsoriatic Agents |
|  |  | EBDAROKIMAB | Not Approved |  |
|  |  | RISANKIZUMAB | Approved |  |
|  |  | BRAZIKUMAB | Not Approved |  |
|  |  | BRIAKINUMAB | Not Approved | Antipsoriatic, for treatment of  multiple sclerosis, antiinflammatory agent |
|  |  | INFLIXIMAB-DYYB | Approved | DMARD, antiinflammatory agent |
|  |  | TILDRAKIZUMAB | Approved |  |
|  |  | ADENOVIRAL TRANSDUCED HIL-12-EXPRESSING AUTOLOGOUS DENDRITIC  CELLS INXN-3001 PLUS ACTIVATOR LIGAND INXN-1001 | Not Approved | Antineoplastic agent |
|  |  | EGEN-001 | Not Approved |  |
|  |  | GUSELKUMAB | Approved |  |
|  |  | HUMANIZED SMART  ANTI-IL-12 ANTIBODY | Not Approved | Antiinflammatory agent |
